# Supplementary material for: Carnelian uncovers hidden functional patterns across diverse study populations from whole metagenome sequencing reads
Source: Genome Biol. 2020 Feb 24;21:47. doi: 10.1186/s13059-020-1933-7 (PMC7038607; doi:10.1186/s13059-020-1933-7)
Supplement: Supplementary file 1 — Additional file 1 Supplementary Notes. Contains Supplementary Notes S1–S9. [file 13059_2020_1933_MOESM1_ESM.pdf]

Carnelian uncovers hidden functional patterns across diverse study populations from whole metagenome sequencing reads

Sumaiya Nazeen[1], Yun William Yu[2], and Bonnie Berger[1,3*]

[1] Computer Science and Artificial Intelligence Laboratory (CSAIL), MIT, Cambridge, MA 02139, USA
[2] Department of Biomedical Informatics, HMS, Boston, MA 02115, USA
[3] Department of Mathematics, MIT, Cambridge, MA 02139, USA
* Corresponding Author: bab@mit.edu

Additional file 1 --- Supplementary Notes S1 – S9

Supplementary Note S1. Comparison of performance between Carnelian, mi-faser, HUMAnN2, and Kraken2 on examples of functionally similar proteins which are not-so-sequence-similar.

Each experiment includes three proteins---A, B, and C, where A and B do not have much similarity at the protein sequence level but have the same enzymatic function (EC label). C has a different enzymatic function and serves as a control. Carnelian, mi-faser, HUMAnN2, and Kraken2 see only A in the reference as a positive example. Carnelian's training data includes shuffled human sequences as negative examples. The test set contains randomly drawn nucleotide reads from back-translated protein sequences of A, B, and C. Reads of length 100 base pairs were drawn from back-translated protein sequences in such a way that every position was covered at least 10 times. Ideally, methods should be able to annotate reads from B with the same function as A and leave the reads from C unannotated. We measured performance in terms of sensitivity, precision, and F1-score.

<u>Example 1:</u>
A: Beta-galactosidase, gene lacZ from Escherichia coli (strain K12). EC label: 3.2.1.23
B: Evolved Beta-galactosidase, gene ebgA from Escherichia coli (strain UTI89 / UPEC). EC label: 3.2.1.23
C: 6-phospho-alpha-glucosidase, gene BET80_00230 from Escherichia coli. EC label: unverified 3.2.1.122 (used as negative here)

Similarity between proteins:
A-B: 34.18% (blastp e-value = $5e^{-177}$), A-C: 28.57% (blastp e-value = 0.018), and B-C: 29.03% (blastp e-value = 2.3)

Method	Sensitivity	Precision	F1-score
Carnelian	0.9857	0.8257	0.8988
mi-faser	0.4857	1.0000	0.6538
HUMAnN2-translated	0.4857	1.0000	0.6538
Kraken2	0.4857	1.0000	0.6538

HUMAnN2-translated, Kraken2 and mi-faser couldn't annotate any reads from B.

<u>Example 2:</u>
A: Acetyl-coenzyme A carboxylase carboxyl transferase subunit alpha, gene accA from Escherichia coli (strain K12). EC label: 6.4.1.2
B: Acetyl-coenzyme A carboxylase carboxyl transferase subunit beta, gene accD from Staphylococcus aureus (strain COL). EC label: 6.4.1.2
C: Pyruvate carboxylase subunit B, gene pycB from Methanocaldococcus jannaschii (strain ATCC 43067 / DSM 2661 / JAL-1 / JCM 10045 / NBRC 100440). EC label: 6.4.1.1 (used as negative here)

Similarity between proteins:
A-B: 58.33% (blastp e-value = 0.13), A-C: 41.18% (blastp e-value = 8.9), and B-C: 30.00% (blastp e-value = 1.7)

Method	Sensitivity	Precision	F1-score
Carnelian	0.7690	0.8162	0.7628
mi-faser	0.5273	1.0000	0.6905
HUMAnN2-translated	0.5273	1.0000	0.6905
Kraken2	0.4552	0.8632	0.5961

HUMAnN2-translated, Kraken2 and mi-faser couldn't annotate any reads from B.

<u>Example 3:</u>
A: Urease subunit beta, gene ureB from Helicobacter felis (strain ATCC 49179 / NCTC 12436 / CS1). EC label: 3.5.1.5

B: Urease subunit alpha, gene ureC from Mycobacterium tuberculosis (strain ATCC 25618 / H37Rv). EC label: 3.5.1.5
C: 4-hydroxyproline 2-epimerase, gene Arad_8151 from Agrobacterium radiobacter (strain K84 / ATCC BAA-868). EC label: 5.1.1.8 (used as negative here)

Similarity between proteins:
A-B: 55.00% (blastp e-value = 0.0), A-C: no significant similarity, and B-C: 40.00% (blastp e-value = 0.11)

Method	Sensitivity	Precision	F1-score
Carnelian	0.9554	0.8198	0.8824
mi-faser	0.6658	1.0000	0.7994
HUMAnN2-translated	0.4961	1.0000	0.6632
Kraken2	0.5879	1.0000	0.7405

Kraken2 and mi-faser were able to annotate some reads from B with correct function, but HUMAnN2-translated was not using the default similarity cut-off.

Supplementary Note S2. Intuition behind the normalization step of Carnelian and supporting experiments.

The input to Carnelian's pipeline is short metagenomic reads from a metagenomic sample and our goal is to determine relative abundances of functional terms (in this case Enzyme Commission (EC) labels) present in the sample. The default assumption is that each coding read represents some gene in part in the microbial sample. We translate these reads to the best possible open reading frames and then bin them into ECs according to some learned representations of proteins from a reference database. All else being equal, the more abundant proteins from an EC bin in the microbial sample is, the more reads from them are likely to be sequenced. Therefore, read counts can be used as a proxy for EC abundance in the sample --- used by common functional annotation tools (e.g. mi-faser). However, in practice "all else" are never equal. These counts need to be made comparable across proteins, samples, and experiments to enable meaningful comparative analysis.

Let's consider the following scenarios.

Scenario 1: Suppose, a microbial sample has only two proteins (from two different ECs) in equal proportion. These protein sequences have different lengths. If we sequence the sample, there is a high possibility that we will see more reads from the longer protein (thus more reads from the corresponding EC). If we take raw read counts as a proxy for relative EC abundance, we will mistakenly assume that the EC with the longer protein is more abundant. This is the reason we need to normalize the read counts in an EC bin by the effective protein length (the positions in the protein sequence to which a read can actually map) of that bin. This value is often known as RPK when the length is measured in kilobases (used by different methods such as HUMAnN2).

Scenario 2: Suppose, we have reads from two experiments with different sequencing depths---one experiment has 10x more reads than the other. If we want to compare the relative abundance of the same EC across experiments, just normalizing by effective protein length in the corresponding EC bin will not change anything. The higher the total number of reads, the higher read count we should expect for any given EC. For relative abundances to be comparable across experiments they need to be on the same scale.

Scenario 3: Suppose we have two microbial samples each with two types of proteins (from two different ECs). Sample 1 has red and yellow proteins and sample 2 has red and green proteins. The lengths of red, yellow, and green proteins are 50, 10, and 250 units respectively. Let's say, we observe 300 reads from both samples and we want to compare the abundance of red proteins across samples. If we observe 50 reads from the red protein in both the sample, the RPK values for red protein will be the same across samples. We observe 250 reads from the yellow protein in sample 1 which means the relative abundance of red protein is much less compared to yellow protein here (RPK for red protein = 1x10^3 vs. RPK for yellow protein = 25 x 10^3). In sample 2, we observe 250 reads from the green protein which means both red and protein have the same relative abundance (RPK for both proteins is 1 x 10^3). This means sample 2 has a higher abundance of red protein which we will not be able to tell if we only compare the RPK values. Clearly, the RPK values of other proteins in the sample have an effect on the relative abundance of a protein in question. If we normalize by the sum of all the RPK values in the sample, then we can see the desired difference (normalized RPK values of red protein in sample 1 and 2 are 1/26 and ½ respectively).

Carnelian's effective count normalization takes the above scenarios into account and normalizes read counts by effective protein length in the EC bin and a per million scaling factor which incorporates the sum of all RPK values in the sample. This normalization ensures that the relative abundances of the EC bins in every sample effectively sums up to the same number making them directly comparable across samples and experiments.

Experiment:

To show how well Carnelian's effective count normalization works in practice, we conducted the following experiment. We randomly selected an individual from our Bostonian cohort. The original read dataset contained ~9M paired-end reads of length 150 base pairs. We created another read dataset by performing 20x down-

sampling such that the new subsampled dataset has ~450k reads. Ideally, the relative abundance of all ECs should be the same in these two samples and we should observe a log fold-change (logFC) of zero (0) for all of them. We used raw read counts (used by mi-faser), RPK measure (used by HUMAnN2), and our effective read counts (TPM measure) as proxies for relative abundance and measured the logFC value for all the ECs in each case. While nearly every EC appears variable between the original and the subsampled dataset in terms of raw read counts and RPKs, only Carnelian's effective counts show the expected behavior.

	Raw count	RPK	Effective Count (Carnelian)
Mean logFC	-1.1251	-1.1251	0.0094
Stddev logFC	0.4659	0.4659	0.1918

Some examples:

EC	Raw count		RPK		Effective Count (Carnelian)	
	Original	Subsampled	Original	Subsampled	Original	Subsampled
3.6.3.19	1090	56	2939.99	151.05	2072.43	2120.91
1.2.1.11	876	45	2442.38	125.46	1721.66	1761.72
5.2.1.8	935	48	3231.71	165.91	2278.07	2329.58
3.6.3.25	2689	138	8280.22	424.94	5836.82	5966.87
4.1.1.87	78	4	187.95	9.64	132.49	135.34
2.7.7.24	3096	153	10927.06	540.00	7702.60	7582.46
4.2.1.24	466	23	1401.50	69.17	987.95	971.30
6.1.1.19	223	11	389.37	19.21	274.47	269.69
4.2.1.8	981	48	2468.74	120.79	1740.24	1696.15
2.5.1.47	3148	154	10092.68	4937.34	7114.44	6932.81

Supplementary Note S3. Test of robustness to biases introduced due to differences in sequencing technologies.

To test for Carnelian's robustness against the biases introduced by different sequencing technologies, we analyzed the sequencing reads generated by Roche 454 FLX Titanium and the Illumina Genome Analyzer (GA) II on the same DNA sample obtained from a complex planktonic community from a temperate freshwater lake (Lake Lanier, Atlanta, GA) from the Luo et al. study [R1]. Raw sequencing reads were downloaded from the JGI Genomic Portal (https://genome.jgi.doe.gov/portal/) with a free account. Carnelian could capture similar functional diversity at both EC and pathway levels (Spearman correlation coefficients 0.87 and 0.89 respectively) despite the differences in sequencing technologies.

Supplementary Note S4. Finding hidden functional patterns in environmental metagenomes.

Since many of the species found in the human microbiome are well annotated and many of the proteins in the reference databases come from human commensal bacteria, Carnelian as well as other functional annotation methods are expected to provide high-quality annotations for metagenomic reads from human body sites. Despite the existence of such bias in the reference dataset, Carnelian can find meaningful biological insights from environmental metagenomic samples which we demonstrated using six aquatic metagenomes from an asbestos mine pit pond in Vermont (VAG-pond data set: [R2] and six beach sand metagenomes from the Deepwater Horizon oil spill site (DWH-spill data set [R3]).

In the VAG-pond data set, we found the functional profiles of the samples from all three layers of the pond to be different from the freshwater samples; they showed high intra-layer correlations and relatively low inter-layer correlation as expected (Table TX1). We identified a number of highly variable ECs which were abundant in the surface layer (epilimnion) where the sunlight, temperature, and amount of dissolved oxygen is higher and less abundant at the middle (hypolimnion) and bottom layers (hypolimnion). For example, EC terms 1.3.15.15, 4.99.1.4, and 2.7.1.177, key players in porphyrin and chlorophyll metabolism, were depleted both in the metalimnion and hypolimnion layers compared to the epilimnion layer (Table TX2). Conversely, EC terms 2.7.4.31, 3.5.4.27, and 4.2.1.147 (implicated in methane metabolism), and 3.1.3.87, 4.1.1.50, and 5.3.1.23 (implicated in sulfur-containing amino-acid metabolism), were found abundant in the hypolimnion layer and depleted in the epilimnion layer (Supplementary Table TX2). Interestingly, several ECs, such as 1.14.11.7 (implicated in sulfur metabolism) were found enriched in the metalimnion layer compared to both epilimnion and hypolimnion layers (Supplementary Table TX2). We also observed high variability in several pathways including the synthesis and degradation of ketone bodies, monobactam biosynthesis, Geraniol degradation, and D-arginine, D-ornithine metabolism between all three layers (Table TX3). Reduced rate of oxidative phosphorylation was observed in hypolimnion compared to epilimnion which was expected in the presence of less dissolved oxygen and sunlight in the bottom layer. Overall, the functional profiles of the samples from the bottom layer showed slightly more functional variability compared to the top two layers as indicated by Shannon-Wiener index (Figure FX1) which agrees with the taxonomic-level findings of the original study [R2].

In the DWH-spill data set, we observed a high intra-phase correlation between the samples (Figure FX2). We also observed much higher functional diversity in the oil and post-oil phases compared to pre-oil phase (Shannon-Wiener Index: pre-oil: 3.43, oil: 5.82 post-oil: 5.78) which suggests a shift in the microbial functionality in the area due to the disastrous event of oil spill. Carnelian-generated functional profiles showed greater abundance of a number of ECs involved in the BTEX (Benzene, Toluene, Ethylbenzene, and Xylenes) degradation pathways in the oil phase compared to the pre-oil phase (Table TX4). Notably, we observed an enrichment of catechol 1,2-dioxygenase (EC 1.13.11.1), catechol-2,3-dioxygenase (1.13.11.2), protocatechuate 3,4-dioxygenase beta chain (1.13.11.3), and muconolactone delta-isomerase (EC 5.3.3.4), key players in the aerobic degradation of aromatic hydrocarbons [R4], in the oil phase samples. Many of the oil-degrading functions were also enriched in the post-oil phase which might suggest that the recovery process may not have finished at the time of sample collection---a finding that agrees with other independent studies of the same data set [R5, R6]. Notably, Carnelian found significant enrichment in all BTEX metabolism pathways in the oil phase (Table TX5).

Table TX1. ***Kendall rank correlation between the functional profiles of VAG-pond samples annotated by Carnelian.***

		Epilimnion			Metalimnion			Hypolimnion		Freshwater	
		1-S	2-S	3-S	1-M	2-M	3-M	2-B	3-B	1-F	2-F
Epilimnion (Surface)	1-S	1.00	-	-	-	-	-	-	-	-	-
	2-S	0.81	1.00	-	-	-	-	-	-	-	-
	3-S	0.83	0.86	1.00	-	-	-	-	-	-	-
Metalimnion (Middle)	1-M	0.79	0.76	0.79	1.00	-	-	-	-	-	-
	2-M	0.73	0.77	0.78	0.74	1.00	-	-	-	-	-
	3-M	0.78	0.80	0.79	0.82	0.78	1.00	-	-	-	-
Hypolimnion (Bottom)	2-B	0.72	0.77	0.75	0.74	0.74	0.78	1.00	-	-	-
	3-B	0.70	0.74	0.73	0.70	0.75	0.74	0.85	1.00	-	-
Freshwater (Control)	1-F	0.57	0.61	0.59	0.59	0.60	0.75	0.65	0.66	1.00	-
	2-F	0.53	0.57	0.56	0.54	0.67	0.60	0.60	0.63	0.60	1.00

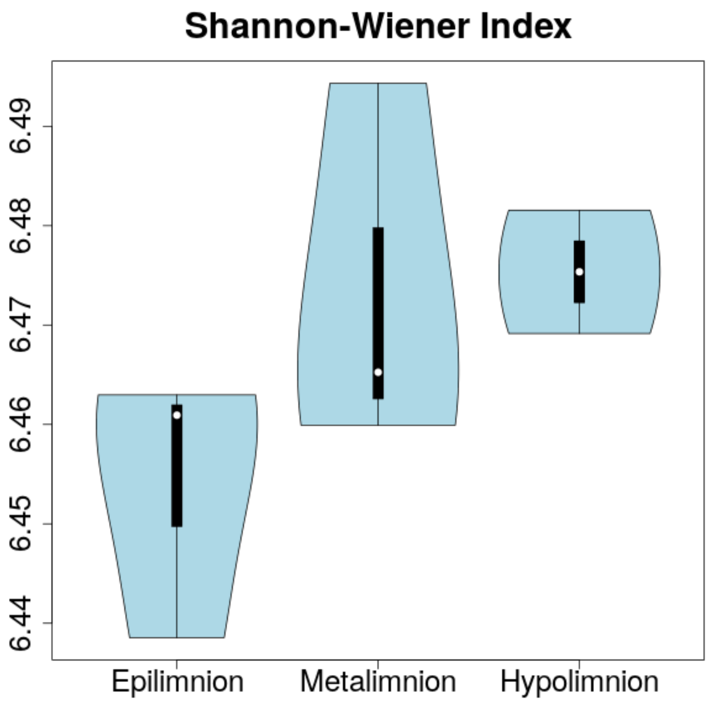

Figure FX1. ***Violin plot showing the functional diversity observed at different layers of the Vermont mine pit pond (VAG-Pond dataset).*** *Samples from the hypolimnion (bottom) layer show higher functional diversity than both epilimnion (surface) and metalimnion (middle) layers as indicated by the Shannon-Wiener indices.*

Table TX2. ***Highly variable ECs between the epilimnion, metalimnion, and hypolimnion layers found by Carnelian in the VAG-pond dataset.***

EC	EpMe_FC	EpHy_FC	MeHy_FC	EC	EpMe_FC	EpHy_FC	Me_Hy_FC
2.7.1.202	6.19	13.82	2.23	4.3.1.7	0.96	0.23	0.22
4.1.1.47	1.02	2.91	2.85	2.7.1.164	0.90	0.25	0.22
2.7.1.177	1.38	2.86	2.07	5.5.1.16	0.86	0.26	0.22
1.3.7.15	1.33	2.86	2.15	2.5.1.97	1.21	0.20	0.24
3.4.17.n1	0.57	2.41	4.24	1.1.1.14	0.78	0.31	0.24
1.1.1.108	0.54	2.09	3.90	1.2.1.80	0.79	0.31	0.24
2.7.7.76	0.35	1.99	5.63	1.4.1.24	0.71	0.35	0.24
5.4.1.4	0.64	1.83	2.85	2.5.1.105	0.71	0.35	0.25
1.14.11.17	0.36	1.06	2.94	3.6.3.12	1.11	0.23	0.25
3.5.4.3	0.25	0.33	0.08	5.3.1.23	1.04	0.24	0.25
4.99.1.4	2.50	3.45	8.33	1.1.1.382	1.52	0.17	0.25
5.1.3.29	0.40	0.31	0.13	3.2.1.37	0.83	0.31	0.26
5.1.99.1	0.53	0.24	0.13	3.6.1.17	0.91	0.28	0.26
1.13.11.48	0.53	0.25	0.13	1.6.1.1	0.76	0.34	0.26
2.1.1.156	0.63	0.23	0.14	4.2.1.82	1.21	0.21	0.26
1.20.4.3	0.56	0.29	0.16	4.2.1.147	1.14	0.23	0.26
2.7.14.1	0.61	0.27	0.17	2.6.1.83	0.89	0.30	0.27
4.1.1.98	0.49	0.34	0.17	2.6.1.59	0.94	0.29	0.27
4.2.1.171	1.13	0.15	0.17	3.5.1.44	1.43	0.19	0.27
1.11.1.6	0.51	0.34	0.18	5.1.3.30	1.00	0.28	0.28
1.4.3.23	0.75	0.25	0.18	4.1.2.27	0.91	0.31	0.28
1.14.99.50	1.21	0.15	0.18	1.12.98.4	0.84	0.34	0.28
1.1.1.412	0.97	0.19	0.19	1.13.11.49	1.10	0.26	0.28
2.7.8.47	0.67	0.28	0.19	4.2.2.22	0.95	0.30	0.28
4.1.1.50	1.00	0.19	0.19	3.4.17.19	0.82	0.35	0.29
2.7.4.31	0.82	0.24	0.19	3.1.3.87	1.04	0.28	0.29
1.1.1.286	0.64	0.31	0.20	3.4.17.14	0.91	0.33	0.30
3.6.1.7	0.75	0.26	0.20	1.1.1.390	0.90	0.33	0.30
3.1.1.17	1.05	0.19	0.20	1.3.1.104	0.86	0.35	0.30
1.21.98.1	1.08	0.19	0.20	3.1.1.61	1.20	0.25	0.30
2.7.1.162	1.09	0.19	0.20	3.2.1.67	0.89	0.34	0.30
4.2.1.83	0.66	0.31	0.20	1.1.1.343	1.25	0.25	0.31
2.4.1.1	0.74	0.29	0.21	4.2.1.5	0.91	0.35	0.32
2.2.1.10	0.67	0.32	0.21	3.5.4.27	1.06	0.31	0.32
1.1.1.374	0.71	0.30	0.21	1.5.3.1	0.96	0.35	0.33
3.6.3.4	0.63	0.34	0.21	4.2.3.156	1.14	0.30	0.34
3.5.1.102	0.69	0.32	0.22	2.4.1.320	1.95	0.18	0.35

Table TX3. ***Highly variable pathways between the epilimnion, metalimnion, and hypolimnion layers found by Carnelian in the VAG-pond dataset.*** *Thresholds used: coverage >= 0.30 and absolute log fold-change > 0.11. Here, coverage is calculated as the ratio of the number of Carnelian-identified ECs mapped to a pathway to the total number of gold standard ECs in the pathway. Here, C = Carbohydrate Metabolism; L = Lipid Metabolism; E = Energy Metabolism; N = Nucleotide Metabolism; AA = Amino Acid Metabolism (includes metabolism of other amino acids as well); SM = Biosynthesis of Secondary Metabolites; G = Glycan Biosynthesis and Metabolism; V = Metabolism of Co-factors and Vitamins; X = Xenobiotics Biodegradation and Metabolism; GI = Genetic Information Processing; T = Metabolism of Terpenoids and Polyketides.*

Category	ID	Pathway	coverage	Epi - Meta logFC	Epi - Hypo logFC	Meta - Hypo logFC
L	00072	Synthesis and degradation of ketone bodies	1.00	0.13	0.37	0.23
SM	00261	Monobactam biosynthesis	0.50	0.17	0.29	0.12
T	00281	Geraniol degradation	0.40	0.09	0.26	0.18
X	00362	Benzoate degradation	0.39	0.07	0.24	0.17
G	00571	Lipoarabinomannan (LAM) biosynthesis	0.50	0.15	0.24	0.10
SM	00332	Carbapenem biosynthesis	0.40	0.09	0.24	0.15
L	01040	Biosynthesis of unsaturated fatty acids	0.32	0.03	0.23	0.20
G	00572	Arabinogalactan biosynthesis - Mycobacterium	0.86	0.13	0.22	0.09
AA	00280	Valine, leucine and isoleucine degradation	0.58	0.06	0.20	0.14
AA	00290	Valine, leucine and isoleucine biosynthesis	0.71	0.01	0.18	0.17
L	00592	alpha-Linolenic acid metabolism	0.33	0.06	0.18	0.12
AA	00310	Lysine degradation	0.39	0.03	0.17	0.14
C	00660	C5-Branched dibasic acid metabolism	0.43	0.01	0.17	0.16
C	00650	Butanoate metabolism	0.58	0.04	0.17	0.13
AA	00440	Phosphonate and phosphinate metabolism	0.38	0.14	0.17	0.03
T	00900	Terpenoid backbone biosynthesis	0.56	0.04	0.17	0.13
L	00071	Fatty acid degradation	0.38	0.03	0.16	0.13
AA	00300	Lysine biosynthesis	0.78	0.08	0.16	0.08
X	00621	Dioxin degradation	0.62	0.07	0.15	0.08
T	00130	Ubiquinone and other terpenoid-quinone biosynthesis	0.49	0.02	0.15	0.13
X	00642	Ethylbenzene degradation	0.67	0.03	0.14	0.11
X	00984	Steroid degradation	0.54	0.11	0.13	0.01
AA	00260	Glycine, serine and threonine metabolism	0.66	0.04	0.12	0.08
V	00785	Lipoic acid metabolism	0.75	-0.03	0.11	0.15
C	00052	Galactose metabolism	0.61	-0.06	-0.11	-0.05
SM	00311	Penicillin and cephalosporin biosynthesis	0.43	-0.01	-0.12	-0.10
E	00190	Oxidative phosphorylation	0.62	0.04	0.13	0.10
C	00500	Starch and sucrose metabolism	0.68	0.00	-0.14	-0.13
G	00511	Other glycan degradation	0.44	-0.01	-0.14	-0.14
G	00515	Mannose type O-glycan biosynthesis	0.38	-0.12	-0.14	-0.02
AA	00472	D-Arginine and D-ornithine metabolism	0.64	-0.07	-0.24	-0.16

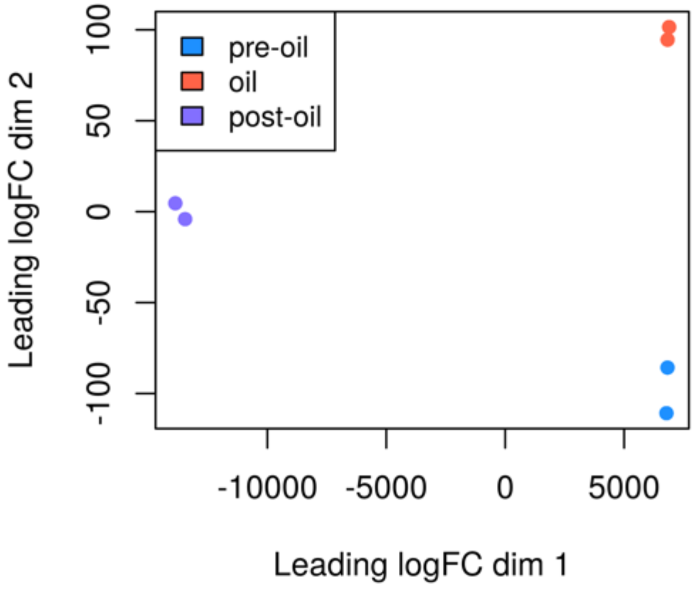

Figure FX2. ***Non-metric multidimensional scaling (NMDS) plot depicting the Carnelian-derived functional profiles of beach sand metagenomes from the DWH-spill dataset.*** *Samples in each phase are functionally similar to each other in both the leading log fold-change dimensions. Samples in the oil-phase are functionally more similar to the samples in the post-oil phase.*

Table TX4. ***Hydrocarbon-degrading ECs involved in BTEX metabolism pathways found enriched in the oil phase compared to the pre-oil phase in DWH-spill dataset by Carnelian.***

EC Number	Name	Oil_by_Pre	Oil_by_Post
1.1.1.35	3-hydroxyacyl-CoA dehydrogenase	2.23	0.97
1.12.98.4	Sulfhydrogenase 1 subunit beta	2.25	0.86
1.13.11.1	Catechol 1,2-dioxygenase	1.97	0.89
1.13.11.2	Catechol-2,3-dioxygenase	2.33	0.97
1.13.11.3	Protocatechuate 3,4-dioxygenase beta chain	1.89	0.90
1.13.11.39	Manganese-dependent 2,3-dihydroxybiphenyl 1,2-dioxygenase	2.58	1.18
1.13.11.57	Gallate dioxygenase	1.99	0.87
1.13.12.16*	Nitronate monooxygenase	2.55	1.89
1.14.11.17	Alpha-ketoglutarate-dependent taurine dioxygenase	2.74	0.99
1.14.12.1*	Anthranilate 1,2-dioxygenase large subunit	3.85	1.49
1.14.13.2	p-hydroxybenzoate hydroxylase (PHBH)	1.78	1.09
1.14.13.24	3-hydroxybenzoate 6-hydroxylase 1	2.26	1.00
1.14.13.7	Phenol 2-monooxygenase	2.13	1.04
1.14.14.1	Cytochrome P450 3A56	2.61	0.94
1.14.14.5	Alkanesulfonate monooxygenase	2.43	0.94
1.14.99.15	Cytochrome p450 CYP199A2	2.29	1.00
1.14.99.39	Ammonia monooxygenase alpha subunit (AMO)	1.74	0.83
1.2.1.10	Acetaldehyde dehydrogenase	2.53	1.50
1.2.1.39	Phenylacetaldehyde dehydrogenase (PAD)	2.19	1.01
1.3.1.32	Maleylacetate reductase 2	1.95	0.85
1.3.8.10*	Cyclohex-1-ene-1-carbonyl-CoA dehydrogenase (Ch1CoA)	3.31	1.58
1.3.8.11*	Cyclohexane-1-carbonyl-CoA dehydrogenase (ChCoA)	2.60	1.47
1.97.1.2	Pyrogallol hydroxytransferase large subunit	1.88	0.88
2.3.1.16	3-ketoacyl-CoA thiolase	2.05	1.18
2.3.1.9	Acetyl-CoA acetyltransferase A	1.68	1.26
2.8.3.12	Glutaconate CoA-transferase subunit A	2.42	0.91
2.8.3.6	3-oxoadipate CoA-transferase subunit A	1.80	0.96
2.8.3.8	Acetate CoA-transferase subunit alpha	2.55	0.95
3.1.1.24	3-oxoadipate enol-lactonase 2	1.89	1.03
3.1.1.45	Putative carboxymethylenebutenolidase	3.37	1.21
3.1.8.1	Aryldialkylphosphatase	2.44	0.84
3.5.1.4	Acetamidase	2.33	0.99
3.5.5.1	Nitrilase 3	2.18	0.95
3.8.1.2	(S)-2-haloacid dehalogenase	3.58	0.94
3.8.1.3	Haloacetate dehalogenase H-1	1.52	0.92
3.8.1.5	Haloalkane dehalogenase	1.52	0.99
3.8.1.7	4-chlorobenzoyl coenzyme A dehalogenase-1	2.19	0.95
4.1.1.61	4-hydroxybenzoate decarboxylase subunit C	2.64	0.93
4.1.1.7	Benzoylformate decarboxylase (BFD)	2.39	0.92
4.1.1.70	Glutaconyl-CoA decarboxylase subunit gamma	1.60	1.03
4.1.3.17	4-carboxy-4-hydroxy-2-oxoadipic acid aldolase	2.25	0.96
4.1.3.39	4-hydroxy-2-oxovalerate aldolase (HOA)	2.44	1.27
4.2.1.17	enoyl-CoA hydratase	2.23	1.22
4.2.1.80	2-keto-4-pentenoate hydratase	2.46	1.19
4.2.1.83	4-oxalmesaconate hydratase (OMA hydratase)	2.38	0.93

Table TX4 (continued). ***Hydrocarbon-degrading ECs involved in BTEX metabolism pathways found enriched in the oil phase compared to the pre-oil phase in the DWH-spill dataset by Carnelian.***

EC Number	Name	Oil_by_Pre	Oil_by_Post
5.1.2.2	Mandelate racemase (MR)	2.25	0.93
5.3.2.8	4-oxalomesaconate tautomerase	2.69	1.12
5.3.3.4	Muconolactone Delta-isomerase (MIase)	3.91	0.92
5.4.4.3	3-hydroxylaminophenol mutase (3HAP mutase)	1.88	1.37
5.5.1.2	3-carboxy-cis,cis-muconate cycloisomerase	2.19	0.94
5.5.1.7	Chloromuconate cycloisomerase	2.04	0.84
6.2.1.32	Anthranilate--CoA ligase	2.47	1.06

Table TX5. ***Highly variable hydrocarbon metabolism pathways found by Carnelian in the DWH-spill dataset.*** *Here, coverage is calculated as the ratio of the number of Carnelian-identified ECs mapped to a pathway to the total number of gold standard ECs in the pathway. Pathways having coverage > 0.30 are reported.*

ID	Pathway	Oil-Pre Fold Change	Oil-Post Fold Change	# ECs mapped	Coverage
00362	Benzoate degradation	15.32	1.23	24	0.85
00627	Aminobenzoate degradation	18.03	1.07	15	0.94
00625	Chloroalkane and chloroalkene degradation	17.15	1.10	11	1.00
00361	Chlorocyclohexane and chlorobenzene degradation	13.48	1.04	9	1.00
00621	Dioxin degradation	21.31	1.31	7	0.88
00623	Toluene degradation	17.50	1.01	6	1.00
00364	Fluorobenzoate degradation	17.49	0.97	5	1.00
00643	Styrene degradation	16.89	1.03	5	0.71
00622	Xylene degradation	19.18	1.29	4	0.80
00642	Ethylbenzene degradation	18.52	1.13	1	0.33
00624	Polycyclic aromatic hydrocarbon degradation	18.73	1.07	1	1.0

Supplementary Note S5. Benchmarking experiments

We first benchmarked Carnelian's gapped k-mer based binning method against the most widely used protein aligners, DIAMOND, PHMMER, and MMSeqs2 using our gold standard database, EC-2010-DB. Then, we benchmarked the full Carnelian pipeline against state-of-the-art alignment-based tools: mi-faser and HUMAnN2, as well as a fast alignment-free tool, Kraken2 using the same gold standard database. Off-the-shelf HUMAnN2 and Kraken2 use taxonomic information in addition to translated searches; to ensure a fair comparison with Carnelian, we used only their "translated-search" modes. All comparisons were based on the estimation of EC terms identified by each method using the same gold standard reference database. The reference databases used by mi-faser and HUMAnN2 and the Kraken2 reference index were created with Carnelian's gold standard reference database for an unbiased comparison.

X5.0 Benchmarks for performance in functional annotation of protein sequences

We compared the performance of Carnelian's gapped k-mer based binning approach against the most widely used protein search and alignment tools: DIAMOND [Buchfink et al., 2014], PHMMER [http://hmmer.org/], and MMSeqs2 [Steinegger et al., 2017]. DIAMOND, PHMMER, and MMSeqs2 rely on sequence alignment for functional annotation, whereas Carnelian's gapped k-mer based binning approach doesn't require sequence alignment.

We compared the performance of these tools in two cases: (i) when query sequences have high sequence similarity to the target sequences in the database (sequence homologs) and (ii) when query sequences are not-so-sequence-similar to the target sequences but have the same enzymatic functions (functional homologs). For the sequence homolog case, we first drew amino acid (AA) fragments, each consisting of 50 residues ensuring every position of the reference proteins is covered at least five times by the fragments. We removed duplicate sequence fragments from the sets. We then divided the fragments into the training and test sets as required for a five-fold cross-validation experiment. All four methods (Carnelian's gapped k-mer based binning, DIAMOND, PHMMER, and MMSeqs2) used the protein fragments contained in the training sets as the reference database and were tested on the protein fragments in the test sets. For the functional homolog case, we mimicked the presence of functionally similar proteins with relatively less sequence similarity by holding out different proportions of proteins from the multi-protein EC bins in our gold standard database and created a test set consisting of amino acid fragments of 50 residues from the held-out proteins. All four methods were trained on the remaining proteins in the multi-protein EC bins and tested on the protein fragments from the held-out proteins.

Note that, DIAMOND, PHMMER, and MMSeqs2 reports all possible alignments for a given query sequence based on some user-defined similarity cutoff. On the other hand, Carnelian's gapped k-mer based binning method reports only the most likely functional label for a query sequence. To make the output of DIAMOND, PHMMER, and MMSeqs2 comparable to Carnelian, we do the following: for DIAMOND, we select the top 1% hits and for PHMMER, we select all the hits with an E-value >= 1e-5. If the hits from a method agree to a single EC label, we consider it as the label for the corresponding query sequence; if the hits contain multiple EC labels, we consider the query to be unannotated. For MMSeqs2, we utilize its "greedy-best-hits" option to report the best possible label chosen by the method. Commands for running the tools are given in Supplementary Nore X9.

We used the macro-averaged sensitivity (ρ), precision (π), and F1-score as evaluation metrics. For each gold-standard functional label (functional bin), *i*, we calculated ρ, π, and F1-score as follows:

$$\pi_i = \frac{TP_i}{TP_i + FP_i},\ \rho_i = \frac{TP_i}{TP_i + FN_i},\ \text{and } F_i = \frac{2\pi_i\rho_i}{\pi_i + \rho_i}$$

Here, TP_i (True Positive) denotes the number of fragments binned correctly under label *i*; FP_i (False Positive) denotes the number of fragments that do not have label *i* but are binned under label *i* by the classifier model; and FN_i (False Negative) is the number of fragments that belong to the bin of label *i* but were incorrectly assigned to some other bin. The overall F1-score of the entire binning problem can be computed by macro averaging, where F1-score for each bin, F_i, is calculated first and then averaged over all bins as:

$$\pi_m = \frac{\sum_{i=1}^{M} \pi_i}{M}, \ \rho_m = \frac{\sum_{i=1}^{M} \rho_i}{M}, \ \text{and} \ F_m = \frac{\sum_{i=1}^{M} F_i}{M}$$

where *M* is the total number of unique functional labels. Macro-averaged measures have advantages over micro-averaged ones because they give equal weight to each functional bin, regardless of how many examples of each label the classifier model has seen in the training set. Thus, the performance of the classifier model is not dominated by common bins; relatively rare categories also get equal importance.

In the sequence homolog case, the performance of Carnelian's gapped k-mer based binning method is comparable to both Diamond and MMSeqs2 (Figure FX3). In the functional homolog case, Carnelian achieves significantly higher sensitivity and F1-score compared to the other methods (Figure FX3).

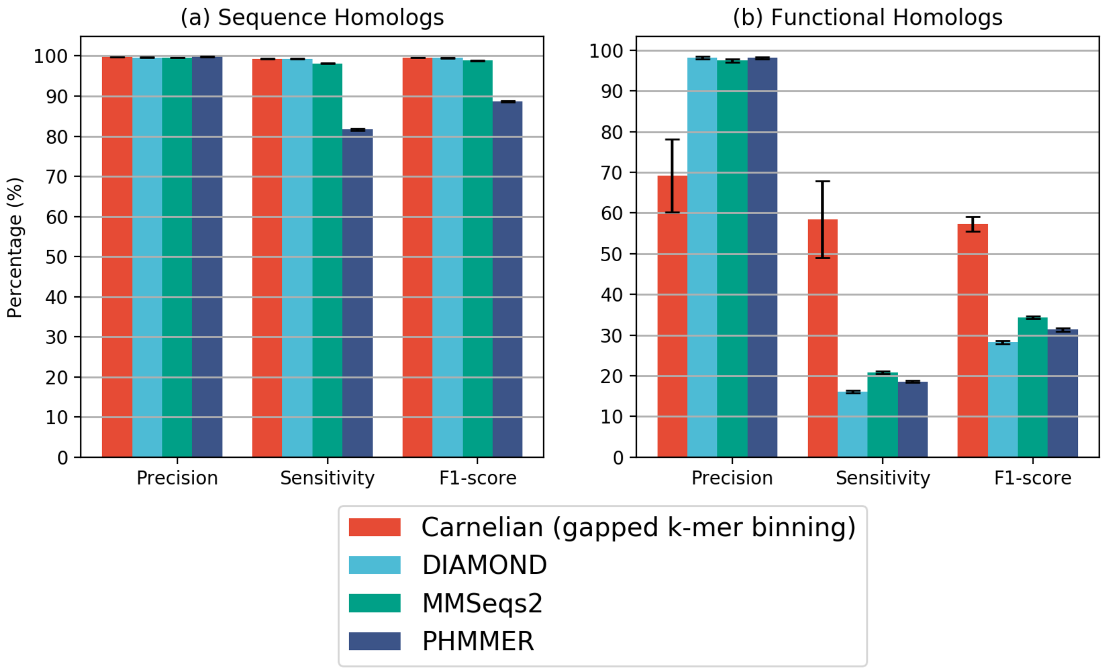

Figure FX3. ***Comparison of the performance of Carnelian's gapped k-mer based binning against DIAMOND, PHMMER, and MMSeqs2. (a) Sequence Homologs.*** *All three methods achieve comparable performance when query protein fragments have high sequence similarity to reference proteins.* ***(b) Functional Homologs.*** *In case of query protein fragments that have only moderate sequence similarity to reference proteins, but have the same functions, Carnelian's gapped k-mer binning achieves significantly higher sensitivity and F1-score. The gapped k-mer based binning technique does achieve lower precision compared to alignment-based methods because, alignment-based methods by design prioritize sensitivity. The error bars indicate standard deviation from the mean.*

X5.1 Benchmarks for performance in functional inference

Testing a tool's capability to infer the functional capacity of a metagenome is very difficult in the absence of true functional labels. To achieve this, we simulated an in-house synthetic human gut metagenome consisting of the 29 common bacterial species that are found in the human gut [R7, R8]. The synthetic metagenome consists of 63% Bacteroidetes (~55% Bacteroides and 8% Alistipes), 6.4% Faecalibacterium, 5.4% Eubacterium, 12.2% Clostridiales, 5% Proteobacteria, 6% Actinobacteria, 1% Fusobacteria, and 1% Verrucomicrobia (Table TX6). This composition closely follows the average proportions of the observed gut microbial genera reported by Louis et al. [R9]. To ensure that our synthetic metagenome has the desired relative abundance distribution of the 29 species, we drew fragments from the pangenomes of each species with probability proportional to the product of the genome's size and corresponding species' target relative abundance. The resultant synthetic metagenome contains 5 million single-ended, 250-nucleotide DNA reads drawn from ChocoPhlAn pangenomes of these 29

species. To create a gold-standard EC profile of the synthetic metagenome, we grouped the Uniref90 gene families present in the synthetic metagenome under ECs using the annotations from UniProt and cross-referenced the EC labels with our gold standard database. The synthetic metagenome contained 9% read with 1067 ECs from our reference database. These ECs were mapped to 127 KEGG metabolic pathways, which we consider as the pathway gold standard.

Table TX6. ***Composition of the in-house synthetic gut metagenome used for functional capacity inference test.*** *Random isolates of the listed species were selected from the ChocoPhlAn database and reads were drawn from their annotated coding sequences ensuring the target coverage in the synthetic metagenome.*

Genera	Species	Abundance	
Bacteroidetes	Bacteroides caccae	0.166	
	Bacteroides cellulosilyticus	0.118	
	Bacteroides dorei	0.084	
	Bacteroides massiliensis	0.058	
	Bacteroides ovatus	0.040	
	Bacteroides stercoris	0.030	
	Bacteroides thetaiotaomicron	0.020	55.00%
	Bacteroides uniformis	0.017	
	Bacteroides vulgatus	0.011	
	Prevotella copri	0.002	
	Parabacteroides merdae	0.002	
	Parabacteroides distasonis	0.001	
	Barnesiella intestinihominis	0.001	
	Alistipes shahii	0.015	
	Alistipes putredinis	0.025	8.00%
	Alistipes onderdonkii	0.040	
Faecalibacterium	Faecalibacterium prausnitzii	0.064	6.40%
Eubacterium	Eubacterium rectale	0.054	5.40%
Clostridiales	Dialister invisus	0.030	
	Roseburia intestinalis	0.030	
	Enterococcus faecium	0.025	12.20%
	Blautia hansenii	0.015	
	Ruminococcus bromii	0.022	
Proteobacteria	Escherichia coli	0.030	5.00%
	Shigella flexnerii	0.020	
Actinobacteria	Bifidobacterium bifidum	0.025	6.00%
	Bifidobacterium longum	0.035	
Fusobacteria	Fusobacterium nucleatum	0.010	1.00%
Verrucomicrobia	Akkermansia muciniphila	0.010	1.00%
Total			100.00%
Uniref_annotated			80.24%
Uniref_unknown			19.76%
EC_annotated			9.01%

Since the generation of synthetic metagenome does not account for the random variations introduced by the fragment sampling and may not give the true magnitude of the EC abundances present in the metagenome, we do not compare against the magnitude of the EC abundances. We calculated sensitivity as the ratio of the number of correct functional terms (EC or pathway) identified by a method to the total number of functional terms (EC or pathway) present in the synthetic metagenome (determined by mapping the Uniref90 gene families present in the sample to gold standard ECs and KEGG pathways). An EC term was considered correctly detected if at least five reads were mapped to it by a method. Precision was calculated as the ratio of the number of correct functional terms identified by a method to the total number of functional terms identified by the method. F1-score was

calculated as the geometric mean of precision and sensitivity. Carnelian achieved **95.88%** sensitivity at the EC level and **98.43%** sensitivity at the pathway level, which were higher than the other three tools (Table TX7).

Table TX7. ***Functional capacity inference on our in-house synthetic gut metagenome.*** *Carnelian achieves higher sensitivity and F1-score at both the EC and pathway levels as compared to mi-faser, HUMAnN2, and Kraken2 searches on a synthetic gut metagenome comprising of the 29 commonly found bacterial species in the human gut. Here, sensitivity is calculated as the ratio of the number of correct functional terms (EC or pathway) identified by a method to the total number of gold standard functional terms (EC or pathway) present in the synthetic metagenome. Precision is calculated as the ratio of the number of correct functional terms identified by a method to the total number of functional terms identified by the method. F1-score is calculated as the geometric mean of precision and sensitivity. Best performances are shown in bold face.*

	Tool	Sensitivity (%)	Precision (%)	F1-score (%)
EC level	Carnelian	**95.88**	**74.23**	**83.68**
	mi-faser	92.03	73.94	82.00
	HUMAnN2	77.69	64.92	70.73
	Kraken2	71.79	66.21	68.88
Pathway level	Carnelian	**98.43**	91.91	**95.06**
	mi-faser	96.06	91.73	93.85
	HUMAnN2	92.91	91.47	92.19
	Kraken2	96.06	**92.42**	94.21

We also compared Carnelian's performance with mi-faser, HUMAnN2, and Kraken2 using the synthetic metagenome described by the HUMAnN2 paper by Franzosa and colleagues [R10]. This metagenome was simulated from the ChocoPhlAn pangenomes of the 20 most abundant bacterial species in Human Microbiome Project (HMP) stool samples [R7]. Species abundances were geometrically staggered from 0.1x to 70x (Table TX8). We ensured that the synthetic metagenome has the desired relative abundance distribution of the 20 species by drawing fragments from the pangenomes of each species with a probability proportional to the product of the genome's size and corresponding species' target relative abundance. The gold-standard EC and pathway profiles of the synthetic metagenome, we grouped the Uniref90 gene families present in the synthetic metagenome under were created using the annotations from UniProt as described above. The synthetic metagenome contained 9% read with 605 ECs from our reference database. These ECs were mapped to 117 KEGG metabolic pathways which we consider as the pathway gold standard. We calculated precision, sensitivity, and F1-score of each method as described earlier. Carnelian achieved **82.15%** sensitivity at the EC level and **90.55%** sensitivity at the pathway level, which were higher than the other three tools (Table TX9).

Like functional capacity inference, performance in functional difference inference is equally difficult to measure. One way to do such benchmark was described by Lindgreen et al. [R11] which we replicated. The data set consisted of different proportions of cyanobacteria (more abundant in set A), *Bradyrhizobium* and *Rhizobium* (more abundant in set A), and known pathogens (more abundant in set B). The shifts in taxa were used as a proxy for the expected pathway shifts between the two sets in the original study. Since the magnitude of the pathway abundances might differ from the differences observed at the taxonomic level, we tested for the direction of the change as suggested by Lindgreen and colleagues. Carnelian identifies the expected direction of change in each of the three categories, where rest of the methods don't (Figure FX4).

Inspired by the above test, we simulated two sets of six complex metagenomes with varying proportions of coding sequences from random isolates of 20 different species of proteobacteria, cyanobacteria, photosynthetic bacteria, nitrogen-fixing bacteria and known pathogens from the ChocoPhlAn database (Table TX10). The gold standard EC and pathway profiles of the metagenomes were created in a similar way as the synthetic gut metagenome described in the main text. Each of the metagenomes contained 5 million single-ended, 250-nucleotide DNA reads, 9% of which had EC annotations. We tested all four methods with the task of detecting the ECs and pathways with the correct direction of the change as demonstrated by the reference profiles.

Table TX8. ***Composition of the HUMAnN2 synthetic gut metagenome used for functional capacity inference test.*** *Random isolates of the listed species were selected from the ChocoPhlAn database and reads were drawn from their annotated coding sequences ensuring the target coverage in the synthetic metagenome.*

Species	# Reads	Proportion
Alistipes onderdonkii	1455410	29.11%
Alistipes putredinis	1031759	20.64%
Alistipes shahii	731372	14.63%
Bacteroides caccae	519394	10.39%
Bacteroides cellulosilyticus	368759	7.38%
Bacteroides dorei	261169	5.22%
Bacteroides massiliensis	185225	3.70%
Bacteroides ovatus	131266	2.63%
Bacteroides stercoris	93525	1.87%
Bacteroides thetaiotaomicron	65776	1.32%
Bacteroides uniformis	46771	0.94%
Bacteroides vulgatus	33147	0.66%
Barnesiella intestinihominis	23559	0.47%
Dialister invisus	16766	0.34%
Eubacterium rectale	11940	0.24%
Faecalibacterium prausnitzii	8770	0.18%
Parabacteroides distasonis	6115	0.12%
Parabacteroides merdae	4226	0.08%
Prevotella copri	2971	0.06%
Ruminococcus bromii	2080	0.04%
Total	5000000	100.00%
Uniref_annotated	4071989	81%
Uniref_unknown	928011	19%
EC_annotated	456107	9%

Table TX9. ***Functional capacity inference on the HUMAnN2 synthetic gut metagenome.*** *Carnelian achieves higher sensitivity and F1-score at both the EC and pathway levels as compared to mi-faser, HUMAnN2, and Kraken2 searches on a synthetic gut metagenome comprising of the 20 most abundant bacterial species in Human Microbiome Project (HMP) stool samples. Here, sensitivity is calculated as the ratio of the number of correct functional terms (EC or pathway) identified by a method to the total number of gold standard functional terms (EC or pathway) present in the synthetic metagenome. Precision is calculated as the ratio of the number of correct functional terms identified by a method to the total number of functional terms identified by the method. F1-score is calculated as the geometric mean of precision and sensitivity. Best performances are shown in bold face.*

	Tool	Sensitivity (%)	Precision (%)	F1-score (%)
EC level	Carnelian	**82.15**	64.21	**72.08**
	mi-faser	74.88	63.45	68.69
	HUMAnN2	30.41	**70.23**	42.45
	Kraken2	59.83	52.24	56.78
Pathway level	Carnelian	**90.55**	86.47	**88.46**
	mi-faser	84.25	91.45	87.70
	HUMAnN2	70.87	**91.84**	80.00
	Kraken2	85.04	84.38	84.71

The reference values for abundances of ECs were calculated by grouping the Uniref90 gene families present in the simulated metagenomes by UniProt annotations and normalizing the summed counts by fragment length and

average gene length per EC label. Similarly, reference values for pathway abundances were determined by mapping the ECs to pathways and summing their abundances. The gold standard directions of the functional changes between two groups were determined by taking the fold-change of the average abundances of each functional term in the two sets. Carnelian achieves slightly higher sensitivity at both EC and pathway level compared to mi-faser and significantly higher sensitivity than HUMAnN2 and Kraken2. Sensitivity is measured as the proportion of functional terms identified by a method with correct direction of shift between two sets (Table TX11).

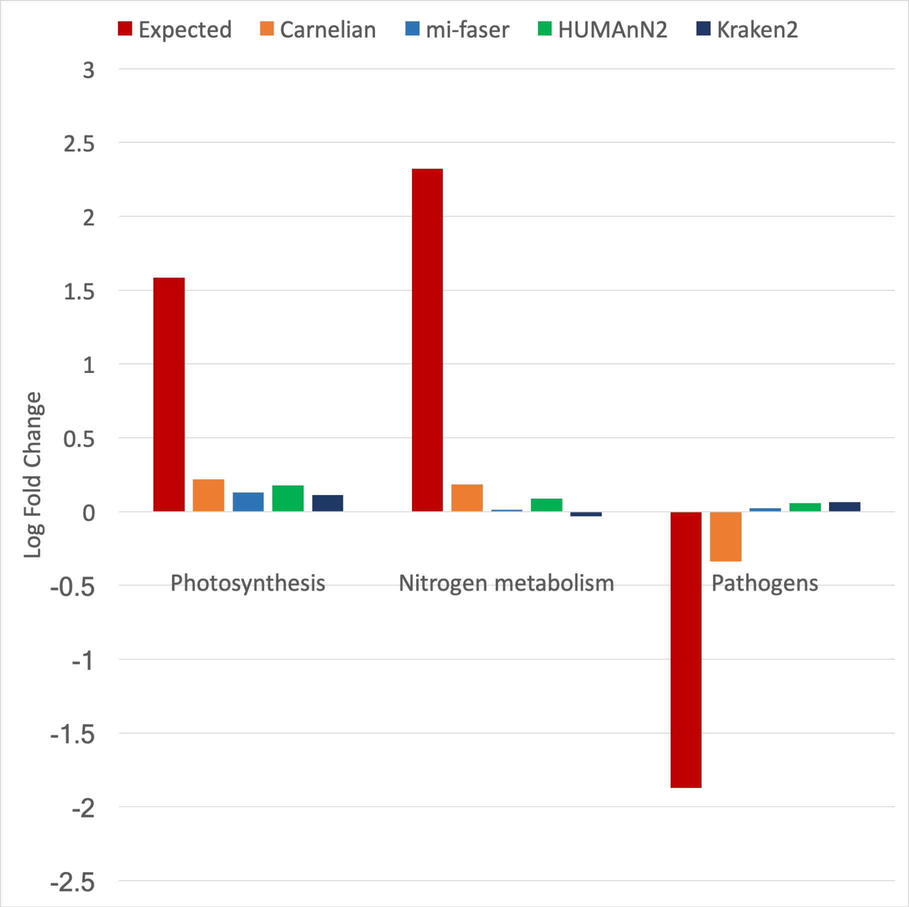

Figure FX4. ***Functional shifts between two sets predicted by Carnelian, mi-faser, HUMAnN2-translated, and Kraken2-translated searches in the functional inference dataset from Lindgreen et al. study*** *[R11]. A positive log fold change means an increase in set A relative to set B and vice versa. The expected fold change amounts were given by the original paper based on the taxonomic differences of the two sets. The test datasets were created with differences in the relative abundance of cyanobacteria (photosynthesis; more abundant in set A), Bradyrhizobium and Rhizobium (nitrogen fixation; more abundant in set A), and known pathogens (more abundant in set B). We profiled the metagenomes from the two sets with all four methods and calculated pathway abundances by mapping the identified ECs to the carbon-fixation, photosynthesis, nitrogen-fixation, two-component systems, bacterial chemotaxis, and cell motility pathways and summing the abundances. Carnelian identifies the expected direction of change in each of the three categories, where rest of the methods don't.*

Table TX10. ***Composition of the dataset used for testing functional change inference.*** *Two sets of six complex metagenomes were created with varying proportions of coding sequences from 20 different species of proteobacteria, cyanobacteria, photosynthetic bacteria, nitrogen-fixing bacteria and known pathogens.*

Species	Set A			Set B			Average Proportions	
	A1	A2	A3	B1	B2	B3	Set A	Set B
Anabaena sp 90	125384	100254	149865	751064	875154	650519	3%	15%
Bradyrhizobium diazoefficiens	249101	224954	274745	49967	50035	40149	5%	1%
Bradyrhizobium elkanii	250393	264246	214801	50136	40075	40236	5%	1%
Campylobacter coli	499756	449839	495120	75506	90185	100080	10%	2%
Chlorobium chlorochromatii	499910	400318	474744	74501	75118	100118	9%	2%
Chloroflexus aurantiacus	499942	600412	525030	99725	99630	75098	11%	2%
Erythrobacter litoralis	500580	549474	505666	74980	109706	75165	10%	2%
Escherichia albertii	50223	74874	24907	250429	274864	299179	1%	5%
Escherichia coli	50138	74624	49633	249811	275877	249701	1%	5%
Helicobacter canadensis	49893	49770	24998	250529	250215	224633	1%	5%
Microcystis aeruginosa	125138	150456	100086	748434	625162	624557	3%	13%
Nodularia spumigena	124423	150483	100215	749842	650422	848959	3%	15%
Nostoc punctiforme	124725	99389	150116	751316	849131	876120	2%	17%
Rhizobium freirei	250230	224940	200277	50064	59833	49784	5%	1%
Rhizobium grahamii	250005	274160	298599	49916	65349	75238	5%	1%
Rhodomicrobium vannielii	500826	525809	400816	99805	49724	60128	10%	1%
Rhodospirillum centenum	499860	475189	600069	75496	75315	90070	11%	2%
Salmonella enterica	50019	24909	74949	249450	224496	199586	1%	4%
Trichodesmium erythraeum	249610	261117	260705	49908	35508	45189	5%	1%
Vibrio campbellii	49844	24783	74659	249121	224201	275491	1%	5%
Total	5000000	5000000	5000000	5000000	5000000	5000000	100%	100%
Uniref annotated	4419647	4421728	4420501	4253105	4240995	4251735	88%	85%
Uniref unknown	580353	578272	579499	746895	759005	748265	12%	15%
EC annotated	456097	401600	446782	421600	456079	465900	9%	9%

Table TX11. ***Inference of functional changes from the two-set complex metagenomes.*** *Carnelian achieves slightly higher sensitivity at both EC and pathway level compared to mi-faser and significantly higher sensitivity than HUMAnN2 and Kraken2. Sensitivity is measured as the proportion of functional terms identified by a method with correct direction of shift between two sets.*

Method	Sensitivity at EC level (%)		Sensitivity at pathway level (%)	
	All ECs	Highly variable ECs (expected abs(logFC) > 1)	All pathways	Highly variable Pathways (expected abs(logFC) > 0.58)
Carnelian	**65.75**	**74.38**	**64.96**	**56.25**
mi-faser	65.13	73.60	63.50	54.17
HUMAnN2	52.77	62.27	60.58	52.08
Kraken2	52.00	59.63	48.18	54.17

X5.2 Accuracy benchmarks on Carnelian's gold standard dataset

To test for accuracy, we benchmarked the tools on synthetic datasets generated from Carnelian's gold-standard reference proteins because, true functional labels for reads in the real world datasets are not available. Three synthetic read datasets with read lengths 150 bp, 200 bp, and 250 bp were constructed each consisting 80% coding and 20% shuffled (non-coding) reads. The coding reads comprised of 30% seen sequences, 15% sequences drawn with 3% mutation rate, 15% sequences drawn with 5% mutation rate from the reference database, and 20% novel reads drawn from prokaryotic proteins with homology based complete EC annotation from the Uniprot/Swissprot database that are not present in our gold standard database.

Carnelian bins more reads than all other methods; especially in the case of novel reads, Carnelian's mappability is significantly better than other methods (Figure FX5 (a)). We achieve significantly higher sensitivity compared to the other methods at comparable accuracy (Figure FX5 (b)).

(a) Mappability of reads **(b) Performance comparison**

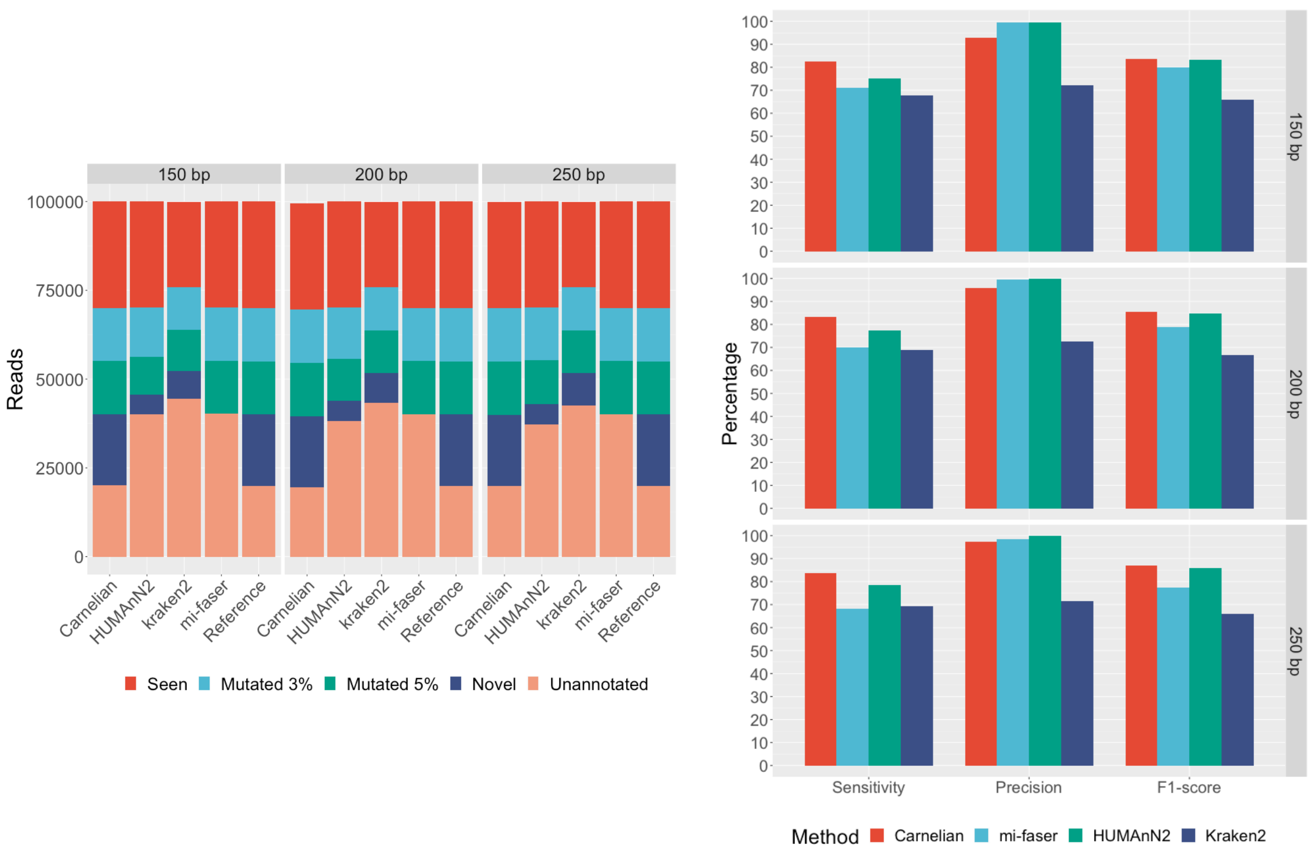

Figure FX5. ***Comparison of Carnelian's performance against mi-faser, HUMAnN2 and Kraken2 on in-house synthetic dataset. (a)*** *Mappability of reads in in-house benchmarking datasets. Three synthetic read datasets were created with read lengths 150, 200, and 250 base pair lengths respectively. Each dataset contained 80% coding reads (Seen: 30%; Mutated 3%: 15%; Mutated 5%: 15\%; Novel: 20%) and 20% shuffled reads. Carnelian maps more coding reads compared to the other three methods.* ***(b)*** *Performance comparison on in-house benchmarking datasets. Carnelian achieves higher sensitivity and F1-score at comparable precision on the benchmarking datasets described in (b) compared to the other three methods.*

We also performed a set of cross-validation experiments: we first drew amino acid (AA) fragments of length, *l = 50 AA, 68AA, 84AA* from the EC-2010-DB sequences ensuring every position of the reference proteins are covered at least five times by the fragments. We removed duplicate fragments from the sets. For each fragment length group, we then divided the fragments into the training and test sets as required for a five-fold cross-validation

experiment. The fragments in the test sets were back-translated using standard codon table to mimic nucleotide reads of lengths 150-bp, 200-bp, and 250-bp. All four methods (Carnelian, mi-faser, HUMAnN2, and Kraken2) used the protein fragments contained in the training sets as the reference database and were tested on the nucleotide fragments in the test sets.

Additionally, we simulated the effect of the presence of a novel protein in metagenomic read data sets by performing two sets of experiments. First, we back-translated reference proteins to nucleotide sequences using standard codon table. We then simulated reads of lengths 150-bp, 200-bp, and 250-bp from those sequences introducing 3% and 5% mutations within the fragments using wgsim. Carnelian's gold standard reference database was used as reference by all four methods and they were tested on the wgsim-generated nucleotide reads with random mutations. To mimic the presence of functionally similar proteins with relatively less sequence similarity, we held out different proportions of proteins from the multi-protein EC bins in our gold standard database and created two test sets by drawing 100-bp and 150-bp fragments from the back-translated held-out proteins. All four methods were trained on the remaining proteins in the multi-protein EC bins and tested on the nucleotide fragments from the held-out proteins.

For longer reads Carnelian achieves higher sensitivity and accuracy compared to all other methods in cross-validation and mutation experiments. Specially in the case of novel proteins, Carnelian demonstrates significant improvement in sensitivity (Figure FX6, Table TX12). We redid the mutation experiments with mi-faser's gold standard database and found similar performance by all the methods (Table TX13). Here, sensitivity, precision, and F1-scores refer to the macro-averaged quantities.

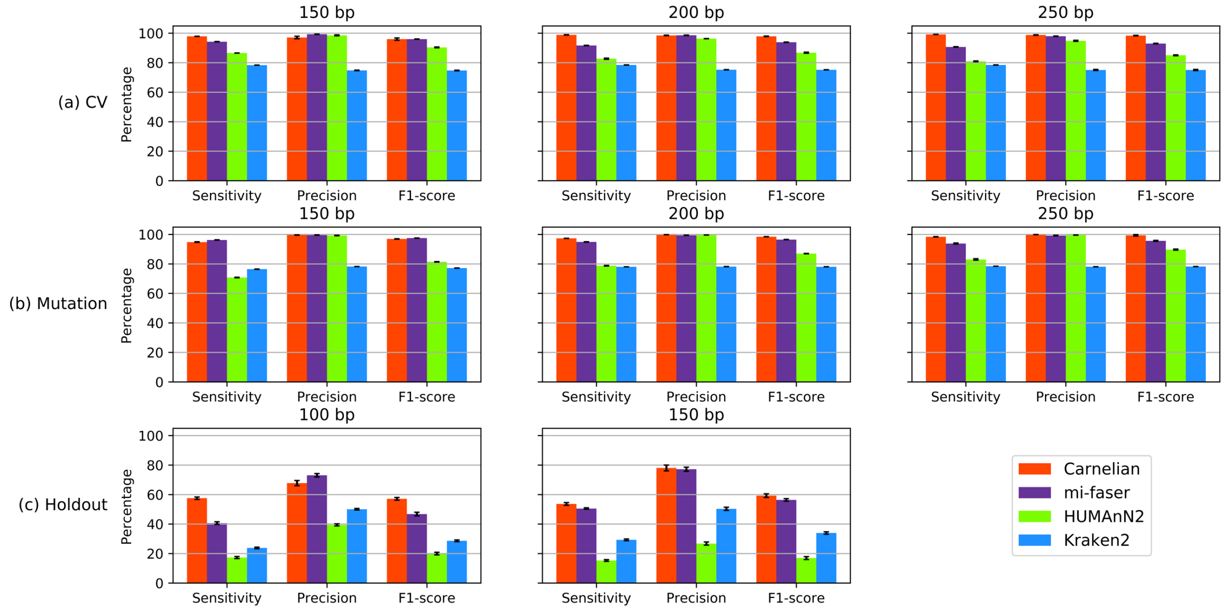

Figure FX6. ***Comparison of Carnelian's performance against mi-faser, HUMAnN2 and Kraken2. (a) 5-fold Cross-validation experiments (testing across ``seen'' proteins).*** *While Carnelian and mi-faser achieve comparable sensitivity and F1-score for 150-bp reads, for longer reads (200-bp, 250-bp), where the field is heading, Carnelian achieves significantly higher sensitivity and F1-score compared to other methods.* ***(b) Mutation (5%) experiments (novel sequences with high similarity).*** *Similar to the cross-validation experiment, Carnelian achieves significantly higher sensitivity and F1-score compared to other methods for longer reads.* ***(c) Hold-out experiments (novel functionally similar proteins with moderate sequence similarity).*** *On short sequences (100-bp and 150-bp -- the longest we could test with available data) from held-out proteins, Carnelian achieves significantly higher sensitivity and F1-score. In all experiments, all the methods demonstrate comparable precision, even though Carnelian alone doesn't perform exact alignment or exact k-mer matching. The error bars indicate standard deviation from the mean.*

Table TX12. ***Performance comparison of Carnelian, mi-faser, HUMAnN2, and Kraken2 on fragments of different lengths with 3% mutations using Carnelian's gold standard dataset.*** *Carnelian achieves higher sensitivity and F1-score than all other methods with comparable precision. Best performances are marked in read.*

Read Length	Method	Mean			Stddev		
		Sensitivity	Precision	F1-score	Sensitivity	Precision	F1-score
150-bp	Carnelian	96.19	99.71	97.73	0.08	0.02	0.08
	mi-faser	95.44	99.48	96.93	0.05	0.06	0.06
	Humann2	92.82	99.90	95.91	0.05	0.03	0.03
	Kraken2	78.15	78.16	78.12	0.04	0.01	0.03
200-bp	Carnelian	98.10	99.84	98.85	0.11	0.05	0.07
	mi-faser	94.32	99.33	96.05	0.14	0.06	0.14
	Humann2	96.46	99.91	97.99	0.26	0.01	0.16
	Kraken2	78.45	78.06	78.21	0.02	0.02	0.02
250-bp	Carnelian	98.87	99.90	99.30	0.04	0.00	0.02
	mi-faser	92.86	98.85	94.78	0.05	0.18	0.08
	Humann2	98.14	99.91	98.74	0.54	0.00	0.03
	Kraken2	78.47	78.00	78.19	0.00	0.02	0.02

Table TX13. ***Performance comparison of Carnelian, mi-faser, HUMAnN2-translated, and Kraken2-translated searches on fragments of different lengths with 3% and 5% mutations using mi-faser's gold standard dataset.*** *For shorted reads mi-faser's performance is slightly better than Carnelian and HUMAnN2. However, for longer reads, Carnelian achieves better sensitivity and recall. Best performances are marked in red.*

Mutation Rate	Read Length	Method	Mean			Stddev		
			Sensitivity	Precision	F1-score	Sensitivity	Precision	F1-score
3%	150-bp	Carnelian	96.46	99.80	98.01	0.10	0.01	0.05
		mi-faser	97.72	99.94	98.58	0.01	0.04	0.02
		Humann2	96.82	99.99	98.34	0.01	0.00	0.01
		Kraken2	76.47	76.58	76.51	0.01	0.01	0.00
	200-bp	Carnelian	98.11	99.88	98.92	0.04	0.01	0.02
		mi-faser	96.69	99.92	97.80	0.03	0.00	0.02
		Humann2	96.54	99.99	98.19	0.08	0.00	0.05
		Kraken2	76.72	76.47	76.57	0.01	0.00	0.01
	250-bp	Carnelian	98.95	99.91	99.37	0.03	0.01	0.02
		mi-faser	95.84	99.92	97.09	0.05	0.00	0.04
		Humann2	97.97	99.99	98.93	0.04	0.00	0.02
		Kraken2	76.73	76.43	76.55	0.00	0.01	0.00
	300-bp	Carnelian	99.41	99.92	99.63	0.02	0.01	0.01
		mi-faser	95.16	99.44	96.44	0.04	0.00	0.04
		Humann2	98.36	99.99	99.18	0.02	0.00	0.01
		Kraken2	76.77	76.42	76.56	0.00	0.00	0.00
5%	150-bp	Carnelian	95.94	98.58	97.03	0.11	0.01	0.06
		mi-faser	98.01	99.97	98.79	0.03	0.04	0.02
		Humann2	82.60	99.99	90.34	0.09	0.00	0.06
		Kraken2	74.88	76.59	75.70	0.02	0.02	0.02
	200-bp	Carnelian	96.80	98.78	97.83	0.01	0.01	0.01
		mi-faser	97.16	99.94	98.16	0.04	0.04	0.03
		Humann2	78.65	99.99	87.84	0.10	0.00	0.07
		Kraken2	76.32	76.51	76.39	0.02	0.01	0.01
	250-bp	Carnelian	97.32	98.77	97.95	0.04	0.01	0.02
		mi-faser	96.40	99.92	97.54	0.06	0.00	0.02
		Humann2	83.65	99.99	90.93	0.18	0.00	0.09
		Kraken2	76.67	76.46	76.54	0.01	0.02	0.01
	300-bp	Carnelian	97.95	98.88	98.31	0.02	0.01	0.02
		mi-faser	95.79	99.73	96.95	0.05	0.05	0.03
		Humann2	85.68	99.99	92.08	0.14	0.00	0.09
		Kraken2	76.75	76.45	76.53	0.01	0.02	0.06

X5.3. Benchmarks for runtime and memory requirement

Carnelian is practical. In terms of running time, our performance closely matches that of HUMAnN2 and is better than the standalone binary of mi-faser (Tables TX14 and TX15). On a synthetic human gut metagenomic dataset of 5 million reads (150 bp, single-ended), Carnelian requires approximately 16 minutes using 16 CPU cores---this is roughly 2x faster than mi-faser (29 minutes) and similar to HUMAnN2 (18 minutes) on the same number of CPUs in the same machine (a 40-core machine with 320 GB RAM, each core was Intel Xeon CPU E5-2695 v2 @ 2.40GHz). Although Kraken2's translated search is the fastest among all four methods, its performance is significantly worse than the other methods in terms of sensitivity and accuracy. Although the memory requirement is larger than mi-faser, HUMAnN2, and Kraken2, this is so because mi-faser and HUMAnN2 use a reduced amino acid alphabet to represent the translated reads. We can get similar memory gains by using reduced size amino acid alphabets without significant loss of precision (Table TX16).

Table TX14. ***Run-time and memory requirement of Carnelian vs mi-faser, HUMAnN2, and Kraken2 when read length varies but database size remains fixed.*** *Five million single-ended reads were generated for each dataset from the synthetic gut metagenome with 20 species. Carnelian's runtime closely matches with HUMAnN2 and is better than mi-faser's standalone binary. Kraken2 is the fastest among all but significantly limited in terms of performance (Main Text). Memory requirements of other three methods are smaller than Carnelian because they all use reduced size amino acid alphabets trading off sensitivity.*

Read length	Method	Description	Elapsed clock time (min)	Max Resident Set Size (GB)
100 bp	Carnelian	Probabilistic gene finding + Composition binning with Opal-Gallager hashing	12.43	13.4
	mi-faser	Custom gene finding + Functional annotation with DIAMOND	22.01	0.17
	Humann2-translated	All six ORF translation + Functional annotation with DIAMOND	12.08	3.33
	Kraken2-translated	All six ORF translation + Functional binning by exact k-mer matching	1.34	0.47
150 bp	Carnelian	Probabilistic gene finding + Composition binning with Opal-Gallager hashing	15.67	13.46
	mi-faser	Custom gene finding + Functional annotation with DIAMOND	29.38	0.20
	Humann2-translated	All six ORF translation + Functional annotation with DIAMOND	17.65	4.94
	Kraken2-translated	All six ORF translation + Functional binning by exact k-mer matching	2.15	0.47
200 bp	Carnelian	Probabilistic gene finding + Composition binning with Opal-Gallager hashing	20.77	13.7
	mi-faser	Custom gene finding + Functional annotation with DIAMOND	37.63	0.28
	Humann2-translated	All six ORF translation + Functional annotation with DIAMOND	24.35	6.49
	Kraken2-translated	All six ORF translation + Functional binning by exact k-mer matching	2.96	0.46
250 bp	Carnelian	Probabilistic gene finding + Composition binning with Opal-Gallager hashing	33.17	13.7
	mi-faser	Custom gene finding + Functional annotation with DIAMOND	44.65	0.34
	Humann2-translated	All six ORF translation + Functional annotation with DIAMOND	30.15	6.53
	Kraken2-translated	All six ORF translation + Functional binning by exact k-mer matching	3.75	0.46

Table TX15. ***Run-time and memory requirement of Carnelian vs mi-faser, HUMAnN2, and Kraken2 when database size varies but read length remains fixed.*** *One million, five million, and 10 million single-ended 250 base pair reads were generated from the synthetic gut metagenome we created using the 20 most abundant species from HMP project. Carnelian's runtime closely matches with HUMAnN2 and is better than mi-faser's standalone binary. Kraken2 is the fastest among all but significantly limited in terms of performance (Main Text). HUMAnN2 and mi-faser are optimized for large compute clusters. As read length increases, the running time of all methods tend to increase. Memory requirement of other three methods are smaller than Carnelian because they all use reduced size amino acid alphabets trading off sensitivity.*

# Reads	Method	Description	Elapsed clock time (min)	Max Resident Set Size (GB)
1 Million	Carnelian	Probabilistic gene finding + Composition binning with Opal-Gallager hashing	9.3	13.7
	mi-faser	Custom gene finding + Functional annotation with DIAMOND	9.0	0.32
	Humann2-translated	All six ORF translation + Functional annotation with DIAMOND	6.5	2.15
	Kraken2-translated	All six ORF translation + Functional binning by exact k-mer matching	0.77	0.43
5 Million	Carnelian	Probabilistic gene finding + Composition binning with Opal-Gallager hashing	33.17	13.7
	mi-faser	Custom gene finding + Functional annotation with DIAMOND	44.65	0.34
	Humann2-translated	All six ORF translation + Functional annotation with DIAMOND	30.15	6.53
	Kraken2-translated	All six ORF translation + Functional binning by exact k-mer matching	3.75	0.45
10 Million	Carnelian	Probabilistic gene finding + Composition binning with Opal-Gallager hashing	54.85	13.7
	mi-faser	Custom gene finding + Functional annotation with DIAMOND	92.48	0.35
	Humann2-translated	All six ORF translation + Functional annotation with DIAMOND	58.9	6.54
	Kraken2-translated	All six ORF translation + Functional binning by exact k-mer matching	7.43	0.46

Table TX16. ***Performance of Carnelian with reduced-size amino acid alphabets on our cross-validation test set containing ~3M 100 bp fragments.***

**Model**	**Alphabet Size (AA)**	**Sensitivity (%)**	**Precision (%)**	**F1-score (%)**	**Peak Memory (GB)**
Full Alphabet	20	98.86	97.86	98.26	7.57
MWL2000[1]	15	86.77	99.52	92.12	0.76
MWL2000[1]	10	86.27	99.59	91.83	0.76
MWL2000[1]	8	78.37	99.18	86.49	0.76
Physico-Chemical[2]	5	86.65	99.53	92.03	0.76
HP Model[3]	2	75.22	98.98	84.22	0.76
mi-faser, HUMAnN2	11[4]	96.78	99.95	98.16	1.31

[1] MWL2000: Murphy, L. R., Wallqvist, A., & Levy, R. M. (2000). Simplified amino acid alphabets for protein fold recognition and implications for folding. *Protein Engineering*, 13(3), 149-152.

[2] Physio-Chemical: Amino acids grouped according to 5 physico-chemical properties — A (Aliphatic): IVL, R (aRomatic): FYWH, C (Charged): KRDE, T (Tiny): GACS, D (Diverse): TMQNP

[3] HP Model: Groups amino acids as polar (hydrophilic) or hydrophobic — P: AGTSNQDEHRKP, H: CMFILVWY

[4] DIAMOND aligner, used by mi-faser and HUMAnN2-translated, inherently represents the proteins in its database with a reduced amino acid alphabet of size 11.

Supplementary Note S6. Performance of out-of-the-box HUMAnN2 on the fecal microbiomes of non-industrialized Baka individuals from Cameroon.

We ran the full pipeline of out-of-the-box HUMAnN2 with ChocoPhlAn, Uniref, and MetaCyc databases on the microbiomes of all the non-industrialized Baka individuals (35 unpublished samples from Alm lab). Each sample has ~7M paired end reads of 150 bp length. It took HUMAnN2 ~ 3 days 6 hours (4676.85 minutes) to annotate the samples using 16 threads on a server with Intel Xeon E5-2695 v2 x86_64 2.40 GHz processor and 320 GB RAM. On the same machine, it would take Carnelian a little over a day (1617 minutes) to bin the reads from this dataset using 16 cpus on the same machine when run in a sequential manner.

On average HUMAnN2 could annotate only 10% reads per Baka sample despite using the entire ChocoPhlAn and Uniref database. On average, HUMAnN2 detected less than 30 species and 996 Enzyme Commission (EC) terms per sample and the average Shannon diversity index per sample was 5.58. For comparison, we also ran out-of-the-box HUMAnN2 on the microbiomes of 20 industrialized Bostonian individuals (unpublished dataset from Alm lab). Each sample had roughly 36M reads on average. Since industrialized microbiomes are well characterized, here HUMAnN2 can annotate more reads (~40-50% per sample). On average it detects 1061 ECs per sample on average (Shannon diversity index 5.95). Uniref IDs were mapped to level 4 EC numbers using the mapping provided by HUMAnN2. Following is a table summarizing the results of full HUMAnN2 pipeline on Baka individuals. For comparison, we include the results from Boston dataset as well as the results of HUMAnN2 translated search and Carnelian (both were run with our curated EC database).

	Out-of-the-box HUMAnN2 (ChocoPhlAn + Uniref + MetaCyc)		HUMAnN2 (translated) (curated EC database)		Carnelian (curated EC database)	
	Baka	Bostonian	Baka	Bostonian	Baka	Bostonian
# reads annotated per sample	722,354	24,527,448	21,383	83,131	269,720	1,430,026
# Species identified per sample	29.6	53.6	N/A	N/A	N/A	N/A
# ECs identified per sample	996	1061	827	791	2003	1981
Shannon diversity index	5.58	5.95	5.79	4.76	6.50	6.49

Note that, full HUMAnN2 pipeline finds less diversity in the microbiomes of non-industrialized Baka individuals compared to the industrialized Bostonian individuals which is counterintuitive [R12]. Using our curated Enzyme Commission database, both HUMAnN2 (translated) and Carnelian can detect more enzymatic diversity in the Baka population.

Supplementary Note S7. Vowpal-Wabbit classifier model used in Carnelian.

Carnelian's ensemble classifier model was implemented using Vowpal-Wabbit (v8.1.1). Vowpal-wabbit implementations perform better than conventional classifiers for large-scale sequence classification tasks. Some of its advantages are as follows: (i) it provides a dedicated of stochastic gradient descent (SGD) which makes the task of learning faster and more scalable compared to standard gradient descent; (ii) the learning can be done in an online fashion which makes retraining the model easier as new annotations become available; and (iii) the keys of the feature hash table can be stored as an integer using MurmurHash3 which saves space.

In ``default'' mode in Carnelian uses Vowpal-Wabbit's one-against-all SVM classifiers. If users want probability scores for the predicted labels, they can use the ``precise'' mode of Carnelian in which we use one-against all logistic regression models from Vowpal-Wabbit. The default parameters used to run Vowpal-Wabbit are as follows:

- oaa: to select the one-against-all classifiers
- passes (Number of Training Passes): 1
- cache: Use a cache
- save_resume: save extra state so learning can be resumed later with new data
- bit precision: 31
- regularization parameters: l1=0, l2=0 for faster training
- To enable "precise" mode:
    - Loss function: logistic
    - probabilities: to get the probabilities for the predictions

Note that, Carnelian gives the user the choice to play with the vowpal-wabbit training parameters, such as the number of passes, regularization parameters *l1* and *l2*, bit precision etc. The values that worked best for our analyses are included as defaults in the pipeline.

Supplementary Note S8. Choice of fragment length and *k*-mer length.

In the training phase, Carnelian trains an ensemble of classifiers in multiple batches. Since our current gold standard is small in size, to ensure that the classifier ensemble sees enough examples per batch, we draw random fragments from the gold standard proteins by making sure that all the reference proteins have sufficient representation in the training batches. While choosing the fragment length (l), we needed to ensure that the fragments we drew were smaller than the smallest protein sequence in our datasets. Lengths of 7,884 protein sequences in EC-2010-DB ranged from 34 to 7,073 amino acids with a median length of 342 amino acids. That's why for training batches we used *l*=30.

Our choice of the value of *k* needs to be such that the chance of *k*-mers being shared by any two protein sequences in our gold standard datasets is minimized. In a study of 1,121 bacterial genomes, Greenfield et al. [R13] showed that for a *k*-mer length of >20 nucleotides (>= 7 amino acids), over 96% of the nucleotide *k*-mers within an organism are unique and only less than 0.2% of the *k*-mers of length 25 nucleotides (>= 8 amino acids) are shared by any two organisms; the 25-mers have the same gene annotation in both genomes. Inspired by these results, we chose *k*=8 for our experiments. For *k*=8, we can calculate the probability, *p* of a random *k*-mer match within a dataset as follows [R14]:

$$p = \frac{1}{\frac{|\Sigma|^k}{g} + 1}$$

where, $|\Sigma|$ is the alphabet size and g is the size of the dataset in total number of amino acid fragments. For a gold standard database containing 32,111,182 randomly sampled amino acid fragments from the EC-2010-DB dataset, this probability is 0.12%, which is sufficiently small. For flexibility, Carnelian takes fragment length as input from the user.

Supplementary Note S9. Commands used for running mi-faser, HUMAnN2, Kraken2, DIAMOND, PHMMER, and MMSeqs2.

mi-faser:
```
# Database construction:
DIAMOND makedb --in <gold_standard_protein_fasta> -d <database>
# Running annotation:
# Single-ended reads
python3.6 mifaser.py -f <input_fasta> -d <path_to_database> -o
<path_to_output> -t 1 -c 1
# Paired-end reads:
python3.6 mifaser.py -l <forward_fq> <reverse_fq> -d <path_to_database> -o
<path_to_output> -t 1 -c 1
```

HUMAnN2: (translated search only)
```
# Database construction and configuration:
DIAMOND makedb --in <gold_standard_protein_fasta> -d <database>
humann2_config --update database_folders protein $DIR
# Running translated search:
humann2 --input <input_file> --output <out_dir> --id-mapping
<ec_mapping_file> --protein-database <database> --bypass-nucleotide-search
# Paired-end reads were put in a single file before running HUMAnN2-
translated search on it as instructed on their website
```

Kraken2-translated: (translated search only)
```
# Index construction:
./kraken2-build --download-taxonomy --db $DBNAME --skip-maps
./kraken2-build --add-to-library $FASTAFILE --db $DBNAME --protein
./kraken2-build --build --db $DBNAME --protein
#Running translation and annotation:
./kraken2 --db $DBNAME --threads <num_threads> --output <out_file>
--use-names <input_file>;
```

DIAMOND:
```
# Database construction and configuration:
DIAMOND makedb --in <gold_standard_protein_fasta> -d <database>
humann2_config --update database_folders protein $DIR
# Running protein search: (report top 1% hits for each query sequence)
DIAMOND blastp –d <dattabase> -q <query_sequence_fasta> -o <output_file>
--top 1
```

PHMMER:
```
# Running protein search: (report all hits with E-value >= 1e-5 for each
query sequence)
phmmer --tblout <output_file> -E 1e-5 -o <temp_directory> --cpu <num_cpus>
<query_sequence_fasta> <reference_database_fasta>
```

MMSeqs2:
```
# protein search reporting greedy best hit for each query sequence
mmseqs easy-search --greedy-best-hits 1 <query_sequence_fasta>
<reference_sequence_fasta> <output_file> <temp_dir>
```

References:

[R1] Luo, Chengwei, et al. "Direct comparisons of Illumina vs. Roche 454 sequencing technologies on the same microbial community DNA sample." *PloS One* 7.2 (2012): e30087

[R2] Driscoll, Heather E., et al. "Metagenomic investigation of the microbial diversity in a chrysotile asbestos mine pit pond, Lowell, Vermont, USA." *Genomics Data* 10 (2016): 158-164.

[R3] Rodriguez-r, Luis M., et al. "Microbial community successional patterns in beach sands impacted by the Deepwater Horizon oil spill." *The ISME Journal* 9.9 (2015): 1928.

[R4] Cao, Bin, Karthiga Nagarajan, and Kai-Chee Loh. "Biodegradation of aromatic compounds: current status and opportunities for biomolecular approaches." *Applied Microbiology and Biotechnology* 85.2 (2009): 207-228.

[R5] Dombrowski, Nina, et al. "Reconstructing metabolic pathways of hydrocarbon-degrading bacteria from the Deepwater Horizon oil spill." *Nature Microbiology* 1.7 (2016): 16057.

[R6] Zhu, Chengsheng, et al. "Functional sequencing read annotation for high precision microbiome analysis." *Nucleic Acids Research* 46.4 (2017): e23-e23.

[R7] Huttenhower, Curtis, et al. "Structure, function and diversity of the healthy human microbiome." *Nature* 486.7402 (2012): 207.

[R8] Rinninella, Emanuele, et al. "What is the healthy gut microbiota composition? a changing ecosystem across age, environment, diet, and diseases." *Microorganisms* 7.1 (2019): 14.

[R9] Louis, Sandrine, et al. "Characterization of the gut microbial community of obese patients following a weight-loss intervention using whole metagenome shotgun sequencing." *PLoS One* 11.2 (2016): e0149564.

[R10] Franzosa, Eric A., et al. "Species-level functional profiling of metagenomes and metatranscriptomes." *Nature Methods* 15.11 (2018): 962.

[R11] Lindgreen, Stinus, Karen L. Adair, and Paul P. Gardner. "An evaluation of the accuracy and speed of metagenome analysis tools." *Scientific Reports* 6 (2016): 19233.

[R12] Segata, Nicola. "Gut microbiome: westernization and the disappearance of intestinal diversity." *Current Biology* 25.14 (2015): R611-R613.

[R13] Greenfield, Paul, and Uwe Roehm. "Answering biological questions by querying k-mer databases." *Concurrency and Computation: Practice and Experience* 25.4 (2013): 497-509.

[R14] Ondov, Brian D., et al. "Mash: fast genome and metagenome distance estimation using MinHash." *Genome Biology* 17.1 (2016): 132.
