# Supplementary material for: Carnelian uncovers hidden functional patterns across diverse study populations from whole metagenome sequencing reads
Source: Genome Biol. 2020 Feb 24;21:47. doi: 10.1186/s13059-020-1933-7 (PMC7038607; doi:10.1186/s13059-020-1933-7)
Supplement: Supplementary file 2 — Additional file 2 Results from Type-2 Diabetes Cohorts. Contains Supplementary Tables S1–S16. [file 13059_2020_1933_MOESM2_ESM.pdf]

Carnelian uncovers hidden functional patterns across diverse study populations from whole metagenome sequencing reads

Sumaiya Nazeen[1], Yun William Yu[2], and Bonnie Berger[1,3*]

[1] Computer Science and Artificial Intelligence Laboratory (CSAIL), MIT, Cambridge, MA 02139, USA
[2] Department of Biomedical Informatics, HMS, Boston, MA 02115, USA
[3] Department of Mathematics, MIT, Cambridge, MA 02139, USA
* Corresponding Author: bab@mit.edu

Additional file 2 --- Results from Type-2 Diabetes Cohorts
Supplementary Tables S1-S16

Supplementary Table S1. Significantly differentially abundant ECs identified by Carnelian in the T2D-Qin data set. Significance thresholds used: BH corrected Wilcoxon rank-sum test *p*-value < 0.05 and abs (log fold change) > 0.33.

EC	t2d-mean	ctrl-mean	logFC	adj pval
5.4.99.62	909.72	707.04	0.36	0.0426
3.6.1.23	2078.62	3926.73	-0.92	0.0457
2.4.2.2	771.45	974.64	-0.34	0.0348
2.4.2.6	124.34	96.80	0.36	0.0168
1.1.1.100	4678.94	5883.54	-0.33	0.0021
2.7.8.35	349.36	261.58	0.42	0.0170
3.7.1.8	84.24	65.11	0.37	0.0044
2.7.2.4	835.20	639.43	0.39	0.0375
1.13.11.27	153.69	120.41	0.35	0.0117
4.1.1.33	226.60	178.72	0.34	0.0015
4.2.1.20	2574.69	2020.65	0.35	0.0036
2.7.1.220	88.93	69.17	0.36	0.0051
1.1.1.408	575.63	451.23	0.35	0.0155
1.12.2.1	208.37	164.88	0.34	0.0310
1.8.4.14	275.14	216.62	0.35	0.0432
1.17.7.4	653.33	893.14	-0.45	0.0149
1.17.7.3	1144.69	1444.67	-0.34	0.0059
1.8.2.3	98.97	78.47	0.33	0.0320
1.1.1.28	543.34	694.93	-0.35	0.0088
3.2.1.52	271.76	342.66	-0.33	0.0242
2.4.1.1	424.73	573.78	-0.43	0.0165
1.4.1.24	216.48	170.00	0.35	0.0179
1.4.1.4	815.62	1051.09	-0.37	0.0004
2.6.1.84	100.26	79.50	0.33	0.0065
2.7.7.61	205.62	162.81	0.34	0.0033
4.2.1.120	166.37	127.05	0.39	0.0050
5.4.2.11	1987.88	2595.24	-0.38	0.0031
4.3.1.15	324.21	256.75	0.34	0.0493
1.3.1.70	119.66	94.68	0.34	0.0003
1.13.11.6	134.86	95.17	0.50	0.0002
4.2.1.147	255.35	199.87	0.35	0.0053
2.4.1.7	281.74	399.26	-0.50	0.0067
5.4.3.2	404.21	304.59	0.41	0.0068
3.1.3.85	50.44	38.46	0.39	0.0424

Supplementary Table S2. Significantly differentially abundant ECs identified by Carnelian in the T2D-Karlsson data set. Significance thresholds used: BH corrected Wilcoxon rank-sum test p-value < 0.05 and abs (log fold change) > 0.33. ECs marked with "*" are significantly variable between impaired glucose tolerance (IGT) and normal glucose tolerance (NGT) individuals as well.

EC	t2d-mean	igt-mean	ngt-mean	t2d-ngt logFC	t2d-ngt adj pval	igt-ngt logFC	igt-ngt adj pval
2.4.1.329	64.76	76.08	82.42	-0.35	0.0130	-0.12	0.4543
4.1.1.101*	176.80	172.82	138.53	0.35	0.0059	0.32	0.0047
4.2.99.20	28.99	29.46	44.30	-0.61	0.0136	-0.59	0.0180
3.1.3.8	49.45	61.84	64.14	-0.38	0.0136	-0.05	0.8640
2.6.1.113*	160.27	143.83	120.42	0.41	0.0309	0.26	0.0188
3.2.2.23	78.82	79.78	116.82	-0.57	0.0211	-0.55	0.2546
4.2.2.n2	66.53	97.47	110.19	-0.73	0.0080	-0.18	0.2417
4.1.1.79	221.75	264.31	281.02	-0.34	0.0083	-0.09	0.4543
1.14.13.127	32.22	33.01	46.41	-0.53	0.0069	-0.49	0.0483
3.5.1.5	1777.62	1597.67	1340.91	0.41	0.0026	0.25	0.1976
3.1.3.12*	149.89	136.66	205.16	-0.45	0.0326	-0.59	0.0200
1.12.98.1	279.91	337.67	369.06	-0.40	0.0072	-0.13	0.3454
6.2.1.44	44.83	51.85	57.72	-0.36	0.0082	-0.15	0.2417
2.3.2.21	81.85	86.17	117.48	-0.52	0.0266	-0.45	0.2546
5.4.99.20	64.57	63.11	90.69	-0.49	0.0398	-0.52	0.1142
2.1.1.10	99.05	80.88	68.53	0.53	0.0040	0.24	0.0837
5.3.1.22	113.81	98.90	186.45	-0.71	0.0091	-0.91	0.0809
1.5.1.36	307.40	374.23	393.30	-0.36	0.0326	-0.07	0.9132
4.2.1.42	100.54	104.18	129.31	-0.36	0.0483	-0.31	0.3868
1.1.1.251	195.67	205.32	254.83	-0.38	0.0007	-0.31	0.0242
4.1.2.48*	54.87	51.91	74.92	-0.45	0.0101	-0.53	0.0020
6.3.2.33	70.08	83.96	98.60	-0.49	0.0013	-0.23	0.1179
1.8.98.1	307.62	357.96	394.34	-0.36	0.0428	-0.14	0.3575
6.3.2.36	93.80	115.64	121.82	-0.38	0.0091	-0.08	0.6347
2.3.1.5	126.23	103.24	97.45	0.37	0.0233	0.08	0.5279
2.8.4.1	480.83	564.56	609.28	-0.34	0.0144	-0.11	0.5028
1.2.99.7	120.53	111.79	95.33	0.34	0.0089	0.23	0.0070
2.4.1.15*	67.74	66.46	97.52	-0.53	0.0184	-0.55	0.0242
6.2.1.3	280.50	304.98	355.19	-0.34	0.0009	-0.22	0.0258
4.2.1.119*	39.05	45.27	29.51	0.40	0.0015	0.62	0.0006
3.4.13.9*	542.93	591.57	883.28	-0.70	0.0016	-0.58	0.0316
3.1.4.16	297.50	348.07	379.14	-0.35	0.0039	-0.12	0.1949
3.1.4.12	36.11	31.03	28.51	0.34	0.0266	0.12	0.5485
2.1.1.90	67.05	79.27	89.75	-0.42	0.0266	-0.18	0.2386
3.2.1.80	289.47	279.54	222.93	0.38	0.0018	0.33	0.0069
2.4.1.182	72.37	76.15	91.88	-0.34	0.0059	-0.27	0.0530
1.13.11.3	148.45	130.25	114.34	0.38	0.0002	0.19	0.0138

Supplementary Table S3. Pathways identified as significantly variable between T2D patients and healthy controls in the T2D-Qin data set using Carnelian-generated functional profiles. Significance thresholds used: BH corrected Wilcoxon rank-sum test p-value < 0.05 and abs (log fold change) > 0.11. Here, C = Carbohydrate Metabolism; L = Lipid Metabolism; E = Energy Metabolism; N = Nucleotide Metabolism; AA = Amino Acid Metabolism (includes metabolism of other amino acids as well); SM = Biosynthesis of Secondary Metabolites; G = Glycan Biosynthesis and Metabolism; V = Metabolism of Co-factors and Vitamins; X = Xenobiotics Biodegradation and Metabolism.

Category	ID	Name	fold-change	logFC	adj pval
C	00010	Glycolysis / Gluconeogenesis	0.87	-0.21	0.0333
C	00020	Citrate cycle (TCA cycle)	0.87	-0.20	0.0478
C	00030	Pentose phosphate pathway	0.86	-0.22	0.0393
C	00040	Pentose and glucuronate interconversions	0.85	-0.23	0.0361
C	00051	Fructose and mannose metabolism	0.86	-0.21	0.0465
C	00052	Galactose metabolism	0.86	-0.22	0.0376
C	00053	Ascorbate and aldarate metabolism	0.84	-0.25	0.0165
L	00061	Fatty acid biosynthesis	1.16	0.21	0.0262
L	00062	Fatty acid elongation	1.17	0.22	0.0372
L	00100	Steroid biosynthesis	1.27	0.34	0.0019
E	00190	Oxidative phosphorylation	1.27	0.35	0.0035
E	00195	Photosynthesis	1.13	0.18	0.0448
N	00240	Pyrimidine metabolism	1.13	0.18	0.0432
AA	00250	Alanine, aspartate and glutamate metabolism	1.14	0.18	0.0018
SM	00254	Aflatoxin biosynthesis	0.88	-0.19	0.0197
AA	00290	Valine, leucine and isoleucine biosynthesis	1.19	0.25	0.0007
AA	00330	Arginine and proline metabolism	1.18	0.23	0.0034
AA	00350	Tyrosine metabolism	1.14	0.19	0.0325
AA	00360	Phenylalanine metabolism	1.15	0.20	0.0366
AA	00480	Glutathione metabolism	1.22	0.28	0.0068
C	00520	Amino sugar and nucleotide sugar metabolism	0.85	-0.23	0.0217
L	00590	Arachidonic acid metabolism	1.21	0.27	0.0071
L	00600	Sphingolipid metabolism	1.19	0.25	0.0221
G	00603	Glycosphingolipid biosynthesis - globo and isoglobo series	1.22	0.28	0.0144
C	00620	Pyruvate metabolism	0.87	-0.19	0.0083
V	00730	Thiamine metabolism	0.88	-0.18	0.0002
V	00750	Vitamin B6 metabolism	0.91	-0.14	0.0054
V	00760	Nicotinate and nicotinamide metabolism	0.85	-0.23	0.0034
V	00780	Biotin metabolism	0.89	-0.17	0.0002
X	00983	Drug metabolism - other enzymes	1.15	0.20	0.0350

Supplementary Table S4. Pathways identified as significantly variable between T2D patients and normal glucose tolerance (NGT) individuals in the T2D-Karlsson data set using Carnelian-generated functional profiles. Significance thresholds used: BH corrected Wilcoxon rank-sum test p-value < 0.05 and abs (log fold change) > 0.11. Here, C = Carbohydrate Metabolism; L = Lipid Metabolism; E = Energy Metabolism; N = Nucleotide Metabolism; AA = Amino Acid Metabolism (includes metabolism of other amino acids as well); SM = Biosynthesis of Secondary Metabolites; G = Glycan Biosynthesis and Metabolism; V = Metabolism of Co-factors and Vitamins; X = Xenobiotics Biodegradation and Metabolism; GI = Genetic Information Processing; T = Metabolism of Terpenoids and Polyketides.

Category	ID	Name	t2d-ngt FC	t2d-ngt logFC	t2d-ngt adj pval	igt-ngt FC	igt-ngt logFC	igt-ngt adj-pval
C	00030	Pentose phosphate pathway	0.87	-0.20	0.0219	0.95	-0.08	0.0268
C	00040	Pentose and glucuronate interconversions	0.86	-0.21	0.0357	0.90	-0.15	0.8701
C	00051	Fructose and mannose metabolism	0.85	-0.24	0.0010	0.91	-0.13	0.1573
C	00052	Galactose metabolism	0.86	-0.22	0.0147	0.96	-0.06	0.0094
L	00061	Fatty acid biosynthesis	1.18	0.24	0.0309	1.08	0.11	0.5331
E	00190	Oxidative phosphorylation	1.21	0.28	0.0128	1.09	0.12	0.8274
E	00195	Photosynthesis	1.27	0.35	0.0067	1.11	0.15	0.6797
AA	00220	Arginine biosynthesis	1.20	0.26	0.0069	1.10	0.13	0.2546
AA	00250	Alanine, aspartate and glutamate metabolism	1.18	0.24	0.0069	1.08	0.11	0.6126
AA	00260	Glycine, serine and threonine metabolism	1.16	0.21	0.0207	1.04	0.06	0.4638
AA	00290	Valine, leucine and isoleucine biosynthesis	1.21	0.27	0.0016	1.10	0.14	0.2481
AA	00300	Lysine biosynthesis	1.18	0.24	0.0147	1.09	0.12	0.1766
SM	00332	Carbapenem biosynthesis	1.21	0.28	0.0117	1.10	0.14	0.3181
AA	00340	Histidine metabolism	1.16	0.21	0.0345	1.05	0.07	0.0456
AA	00400	Phenylalanine, tyrosine and tryptophan biosynthesis	1.20	0.26	0.0047	1.12	0.16	0.0492
SM	00405	Phenazine biosynthesis	1.22	0.28	0.0191	1.13	0.18	0.0492
AA	00450	Selenocompound metabolism	1.16	0.21	0.0017	1.08	0.11	0.0539
AA	00460	Cyanoamino acid metabolism	1.24	0.31	0.0091	1.06	0.08	0.4831
AA	00472	D-Arginine and D-ornithine metabolism	1.10	0.14	0.0492	1.06	0.09	0.7318
G	00511	Other glycan degradation	1.17	0.22	0.0215	1.06	0.08	0.4879
L	00561	Glycerolipid metabolism	1.19	0.25	0.0002	1.12	0.16	0.0316
L	00590	Arachidonic acid metabolism	1.20	0.26	0.0314	1.17	0.22	0.0144
L	00600	Sphingolipid metabolism	1.20	0.26	0.0332	1.06	0.09	0.6402
L	00604	Glycosphingolipid biosynthesis - ganglio series	1.16	0.21	0.0130	1.03	0.05	0.7912
V	00730	Thiamine metabolism	0.86	-0.22	0.0377	0.90	-0.15	0.7673
GI	00970	Aminoacyl-tRNA biosynthesis	1.11	0.15	0.0428	1.07	0.10	0.7792
X	00983	Drug metabolism - other enzymes	1.18	0.24	0.0082	1.09	0.12	0.5695

Supplementary Table S4 (continued). Pathways identified as significantly variable between T2D patients and normal glucose tolerance (NGT) individuals in the T2D-Karlsson data set using Carnelian-generated functional profiles. Significance thresholds used: BH corrected Wilcoxon rank-sum test p-value < 0.05 and abs (log fold change) > 0.11. Here, C = Carbohydrate Metabolism; L = Lipid Metabolism; E = Energy Metabolism; N = Nucleotide Metabolism; AA = Amino Acid Metabolism (includes metabolism of other amino acids as well); SM = Biosynthesis of Secondary Metabolites; G = Glycan Biosynthesis and Metabolism; V = Metabolism of Co-factors and Vitamins; X = Xenobiotics Biodegradation and Metabolism; GI = Genetic Information Processing; T = Metabolism of Terpenoids and Polyketides.

Category	ID	Name	t2d-ngt FC	t2d-ngt logFC	t2d-ngt adj pval	igt-ngt FC	igt-ngt logFC	igt-ngt adj-pval
X	00791	Atrazine degradation	1.38	0.47	0.0031	1.31	0.39	0.1843
V	00830	Retinol metabolism	1.22	0.28	0.0297	1.13	0.17	0.3414
C	00660	C5-Branched dibasic acid metabolism	1.21	0.27	0.0017	1.09	0.13	0.1161
T	00900	Terpenoid backbone biosynthesis	1.18	0.24	0.0083	1.05	0.08	0.8517
E	00910	Nitrogen metabolism	1.19	0.25	0.0063	1.07	0.10	0.3534
E	00920	Sulfur metabolism	1.17	0.22	0.0037	1.07	0.10	0.1505
X	00624	Polycyclic aromatic hydrocarbon degradation	1.49	0.57	0.0002	1.30	0.38	0.0138
X	00625	Chloroalkane and chloroalkene degradation	1.18	0.24	0.0276	1.07	0.10	0.7201
X	00626	Naphthalene degradation	1.23	0.30	0.0371	1.12	0.16	0.2031

Supplementary Table S5. Significantly differentially abundant ECs identified by mi-faser in the T2D-Qin data set. Significance thresholds used: BH corrected Wilcoxon rank-sum test p-value < 0.05 and abs (log fold change) > 0.33.

EC	fold-change	logFC	adj pval	EC	fold-change	logFC	adj pval
2.7.1.58	1.85	0.89	0.0379	2.1.1.289	0.77	-0.37	0.0174
6.2.1.13	1.26	0.34	0.0188	2.7.1.220	0.61	-0.72	0.0406
1.2.3.3	1.70	0.77	0.0176	3.5.3.8	0.10	-3.34	0.0082
2.7.1.113	1.80	0.85	0.0293	3.5.3.1	1.31	0.39	0.0351
4.1.1.48	1.31	0.39	0.0444	2.4.1.288	5.62	2.49	0.0095
6.1.2.1	1.30	0.38	0.0400	5.1.99.1	1.37	0.45	0.0027
4.2.1.162	0.76	-0.39	0.0120	2.7.1.162	1.48	0.57	0.0452
2.5.1.88	1.59	0.67	0.0012	5.4.2.8	0.70	-0.52	0.0484
1.1.3.48	2.01	1.01	0.0213	5.1.3.23	1.89	0.92	0.0480
5.4.99.61	0.63	-0.67	0.0256	3.2.1.136	1.41	0.49	0.0470
2.4.2.6	1.78	0.83	0.0059	1.1.1.28	1.28	0.36	0.0023
2.6.1.34	1.28	0.35	0.0103	1.3.1.101	0.70	-0.52	0.0327
2.6.1.39	1.48	0.56	0.0023	2.4.1.8	1.36	0.44	0.0102
1.1.1.215	1.96	0.97	0.0349	6.3.1.12	2.14	1.10	0.0004
4.1.99.1	1.28	0.35	0.0221	1.1.1.377	1.55	0.63	0.0029
2.7.8.36	1.29	0.37	0.0077	1.1.1.371	1.46	0.54	0.0002
2.7.8.38	1.76	0.82	0.0101	4.2.1.120	1.62	0.69	0.0012
2.4.2.45	0.39	-1.37	0.0022	3.2.1.89	0.70	-0.51	0.0279
2.1.1.264	0.55	-0.86	0.0052	2.3.1.245	1.38	0.47	0.0047
5.3.99.11	0.14	-2.83	0.0160	4.3.1.14	1.28	0.35	0.0386
5.1.1.13	2.00	1.00	0.0053	2.7.1.76	2.06	1.05	0.0108
1.1.1.310	1.62	0.70	0.0075	3.2.1.99	0.57	-0.82	0.0274
2.6.1.17	1.55	0.63	0.0439	3.1.3.90	46.58	5.54	0.0219
2.7.1.95	3.69	1.88	0.0211	2.4.1.345	1.64	0.72	0.0041
1.3.8.2	2.41	1.27	0.0003	5.1.99.1	1.37	0.45	0.0027
1.13.11.27	0.03	-4.84	0.0326	2.7.1.162	1.48	0.57	0.0452
3.2.1.11	0.34	-1.58	0.0243	5.4.2.8	0.70	-0.52	0.0484
4.1.1.86	0.74	-0.44	0.0127	5.1.3.23	1.89	0.92	0.0480
4.1.1.33	62.63	5.97	0.0043	3.2.1.136	1.41	0.49	0.0470
4.2.1.5	2.88	1.53	0.0282	1.1.1.28	1.28	0.36	0.0023
1.3.1.12	0.45	-1.16	0.0295				

Supplementary Table S6. Significantly differentially abundant ECs identified by HUMAnN2 in the T2D-Qin data set. Significance thresholds used: BH corrected Wilcoxon rank-sum test p-value < 0.05 and abs (log fold change) > 0.33.

EC	fold-change	logFC	adj pval
4.6.1.12	0.76	-0.39	0.0054
4.4.1.25	3.33	1.74	0.0307
4.1.1.48	1.67	0.74	0.0096
6.1.2.1	3.83	1.94	0.0270
2.4.1.329	0.76	-0.40	0.0038
1.6.5.8	0.58	-0.78	0.0187
3.1.3.3	0.77	-0.39	0.0065
4.1.99.2	0.62	-0.68	0.0130
1.1.1.310	7.55	2.92	0.0356
1.1.1.304	0.55	-0.85	0.0331
2.7.1.95	3.68	1.88	0.0423
2.7.1.205	0.56	-0.85	0.0026
3.2.1.18	0.64	-0.64	0.0288
4.1.1.86	0.44	-1.19	0.0219
1.12.5.1	1.33	0.42	0.0070
2.3.1.30	0.78	-0.35	0.0119
3.1.3.73	0.65	-0.62	0.0277
2.4.1.282	0.35	-1.51	0.0189
3.4.24.3	5.11	2.35	0.0102
1.1.1.39	1.31	0.39	0.0365
1.2.1.92	11.50	3.52	0.0120
5.4.2.8	0.60	-0.75	0.0295
2.3.3.3	1.58	0.66	0.0150
5.1.3.23	2.76	1.47	0.0176
3.2.1.135	1.94	0.96	0.0095
3.3.1.1	1.26	0.34	0.0291
1.97.1.2	2.20	1.14	0.0340
4.2.1.119	1.35	1.14	0.0152
6.3.1.12	6.05	2.60	0.0069
2.1.1.228	0.78	-0.36	0.0430
2.4.99.16	3.05	1.61	0.0414
4.3.3.6	0.79	-0.33	0.0261
2.7.1.76	1.82	0.86	0.0197
3.6.3.42	2.12	1.09	0.0367
1.3.7.5	0.35	-1.51	0.0072
3.1.3.83	1.36	0.44	0.0144

Supplementary Table S7. Significantly differentially abundant ECs identified by Kraken2 in the T2D-Qin data set. Significance thresholds used: BH corrected Wilcoxon rank-sum test p-value < 0.05 and abs (log fold change) > 0.33.

EC	fold-change	logFC	adj pval	EC	fold-change	logFC	adj pval
2.7.7.13	1.52	0.60	0.0433	3.4.24.75	0.32	-1.66	0.0205
1.7.7.2	1.70	0.77	0.0079	1.1.1.336	1.42	0.50	0.0432
1.13.11.73	0.75	-0.41	0.0120	1.1.1.338	0.02	-5.93	0.0453
2.4.1.329	0.77	-0.37	0.0055	1.14.13.92	0.07	-3.91	0.0441
6.3.2.49	2.32	1.21	0.0482	2.3.3.10	0.37	-1.43	0.0435
2.4.2.2	0.75	-0.41	0.0371	2.7.1.39	0.66	-0.60	0.0334
2.4.2.6	68.89	6.11	0.0424	3.4.11.15	2.49	1.32	0.0167
3.5.1.93	0.67	-0.58	0.0356	1.13.11.2	3.29	1.72	0.0399
1.7.2.1	1.27	0.34	0.0092	1.15.1.2	0.67	-0.57	0.0462
2.6.1.39	1.74	0.80	0.0280	1.1.1.28	1.92	0.94	0.0320
2.5.1.96	3.26	1.71	0.0438	1.97.1.2	2.20	1.14	0.0137
6.3.2.n2	1.92	0.94	0.0212	3.4.23.42	0.42	-1.26	0.0172
2.4.1.250	0.75	-0.41	0.0285	3.2.1.1	1.29	0.36	0.0189
2.4.1.332	0.41	-1.27	0.0032	4.99.1.3	0.50	-1.01	0.0149
2.4.1.247	0.75	-0.41	0.0380	2.1.1.80	0.67	-0.57	0.0257
1.17.8.1	0.75	-0.42	0.0124	1.3.1.101	0.79	-0.35	0.0075
5.1.1.13	1.48	0.57	0.0488	1.3.1.54	0.34	-1.56	0.0111
2.7.4.2	0.52	-0.93	0.0089	1.5.99.13	0.69	-0.53	0.0274
1.14.16.1	6.66	2.74	0.0083	4.2.1.120	1.95	0.96	0.0210
3.6.4.9	3.11	1.64	0.0080	3.2.1.89	0.66	-0.60	0.0247
4.2.2.22	11.79	3.56	0.0225	3.4.19.1	0.08	-3.72	0.0343
3.2.2.3	1.53	0.61	0.0229	1.13.11.9	4.00	2.00	0.0491
1.3.3.11	0.54	-0.90	0.0478	2.5.1.68	0.65	-0.63	0.0205
2.6.1.77	1.86	0.90	0.0470	4.1.1.39	1.55	0.64	0.0256
3.5.1.44	0.50	-0.99	0.0162				

Supplementary Table S8. Significantly differentially abundant ECs identified by mi-faser in the T2D-Karlsson data set. Significance thresholds used: BH corrected Wilcoxon rank-sum test p-value < 0.05 and abs (log fold change) > 0.33. Here, igt = impaired glucose tolerance; ngt = normal glucose tolerance.

EC	t2d-ngt-FC	t2d-ngt-logFC	t2d-ngt-adj-p	igt-ngt-FC	igt-ngt-logFC	igt-ngt-adj-p
3.1.22.4	0.55	-0.86	0.0159	0.47	-1.09	0.0037
4.6.1.16	0.37	-1.45	0.0188	0.86	-0.22	0.6484
2.5.1.77	0.46	-1.11	0.0494	0.77	-0.38	0.2929
2.3.1.169	1.80	0.84	0.0099	1.34	0.42	0.5426
1.2.7.4	1.28	0.36	0.0443	1.15	0.20	0.5416
1.2.7.8	0.77	-0.38	0.0398	0.86	-0.22	0.1310
2.1.1.217	2.95	1.56	0.0451	2.38	1.25	0.4678
3.5.4.10	0.58	-0.79	0.0098	0.97	-0.04	0.2118
1.1.1.136	0.65	-0.61	0.0488	0.74	-0.43	0.0458
1.1.1.302	0.78	-0.35	0.0234	0.89	-0.17	0.2101
2.7.7.23	0.79	-0.34	0.0475	0.73	-0.45	0.0895
4.4.1.19	0.47	-1.08	0.0295	0.84	-0.26	0.2198
4.2.2.6	0.17	-2.56	0.0386	0.00	-8.00	0.0149
4.1.1.15	0.76	-0.40	0.0323	0.62	-0.68	0.0015
5.4.99.60	1.37	0.45	0.0124	0.97	-0.05	0.8942
2.4.1.11	0.42	-1.27	0.0068	0.39	-1.35	0.0434
2.4.1.19	0.53	-0.92	0.0276	0.38	-1.40	0.0035
2.4.1.329	0.48	-1.07	0.0031	0.89	-0.16	0.6834
5.1.2.1	1.36	0.44	0.0420	1.45	0.54	0.0659
3.2.1.185	1.55	0.63	0.0026	1.40	0.48	0.2105
4.1.1.101	1.94	0.96	0.0127	1.65	0.72	0.3697
3.5.4.27	0.46	-1.11	0.0184	0.72	-0.47	0.1579
2.6.1.109	0.41	-1.27	0.0042	0.70	-0.51	0.0805
1.1.1.107	2.43	1.28	0.0002	1.44	0.53	0.0424
2.6.1.34	0.54	-0.88	0.0057	0.88	-0.18	0.0971
4.2.1.36	1.41	0.50	0.0051	1.60	0.68	0.0591
2.7.8.35	3.14	1.65	0.0063	2.15	1.11	0.0363
3.5.4.39	0.47	-1.10	0.0098	0.75	-0.41	0.1587
2.4.2.48	0.59	-0.76	0.0331	0.98	-0.03	0.2412
3.2.2.20	0.78	-0.35	0.0357	0.86	-0.21	0.1055
1.2.1.22	0.59	-0.76	0.0315	0.79	-0.35	0.1693
4.1.99.14	0.45	-1.15	0.0258	0.78	-0.35	0.4157
2.7.1.85	1.43	0.52	0.0359	0.38	-1.39	0.8280
4.1.1.79	0.52	-0.95	0.0071	0.76	-0.40	0.0967
4.1.1.75	1.59	0.67	0.0230	0.91	-0.13	0.7452
2.3.1.136	6.27	2.65	0.0386	0.92	-0.12	0.6655
2.1.1.74	0.79	-0.34	0.0405	0.96	-0.05	0.4831

Supplementary Table S8 (continued). Significantly differentially abundant ECs identified by mi-faser in the T2D-Karlsson data set. Significance thresholds used: BH corrected Wilcoxon rank-sum test p-value < 0.05 and abs (log fold change) > 0.33. Here, igt = impaired glucose tolerance; ngt = normal glucose tolerance.

EC	t2d-ngt-FC	t2d-ngt-logFC	t2d-ngt-adj-p	igt-ngt-FC	igt-ngt-logFC	igt-ngt-adj-p
2.4.2.4	0.5	-0.99	0.0041	0.39	-1.35	0.0009
2.4.2.54	0.52	-0.95	0.0122	0.86	-0.22	0.1337
1.3.4.1	0.57	-0.8	0.0108	0.81	-0.3	0.0657
3.4.16.4	0.79	-0.34	0.0371	0.74	-0.44	0.052
3.5.1.2	0.69	-0.55	0.016	0.56	-0.84	0.0012
3.5.1.5	1.42	0.51	0.0047	1.29	0.37	0.052
1.5.1.49	0.5	-0.99	0.0245	0.97	-0.04	0.2489
1.5.1.40	0.51	-0.98	0.041	0.64	-0.64	0.1291
3.6.4.9	0.56	-0.83	0.0159	0.86	-0.21	0.1579
2.4.2.29	0.79	-0.34	0.0015	0.96	-0.06	0.2031
1.1.1.261	0.58	-0.79	0.0286	0.79	-0.35	0.0895
1.1.1.311	1.71	0.77	0.0001	1.7	0.76	0.0002
1.12.98.2	0.53	-0.92	0.0273	0.78	-0.35	0.1814
1.1.1.87	0.77	-0.39	0.0391	0.97	-0.05	0.907
2.8.3.12	1.47	0.56	0.0133	0.92	-0.12	0.9438
2.1.1.10	2.51	1.33	0.0044	1.68	0.75	0.2677
4.2.1.40	0.69	-0.53	0.0364	0.71	-0.5	0.0744
2.4.1.54	3.03	1.6	0.0061	1.81	0.86	0.2544
2.2.1.10	0.5	-1.01	0.0498	0.66	-0.59	0.0523
1.12.7.2	0.71	-0.49	0.0027	0.87	-0.19	0.1095
2.8.1.10	1.43	0.52	0.0001	1.09	0.12	0.2059
2.7.4.26	0.43	-1.21	0.0051	0.79	-0.34	0.0689
2.1.1.246	0.35	-1.52	0.0404	0.84	-0.25	0.3333
1.1.1.405	0.74	-0.44	0.0246	0.87	-0.21	0.511
2.1.1.171	0.7	-0.52	0.0433	0.5	-1	0.0018
2.5.1.114	0.49	-1.03	0.0099	0.8	-0.33	0.2767
3.2.1.70	1.38	0.47	0.0451	2.42	1.28	0.0035
1.1.1.39	1.27	0.34	0.0163	0.98	-0.03	0.7722
3.4.13.22	0.27	-1.88	0.0478	0.36	-1.46	0.3035
1.2.99.7	1.55	0.63	0.0014	1.26	0.34	0.0636
3.1.21.3	0.76	-0.4	0.0017	0.83	-0.27	0.0451
5.1.3.21	3.39	1.76	0.0448	2.53	1.34	0.2146
2.7.7.1	0.51	-0.97	0.006	0.89	-0.17	0.1514
1.5.98.1	0.53	-0.92	0.0124	0.85	-0.24	0.1579
1.5.98.2	0.61	-0.7	0.0298	0.91	-0.13	0.1898
4.2.3.153	0.51	-0.97	0.037	0.8	-0.32	0.1561
3.1.3.45	0.52	-0.93	0.0487	0.22	-2.17	0.0001

Supplementary Table S8 (continued). Significantly differentially abundant ECs identified by mi-faser in the T2D-Karlsson data set. Significance thresholds used: BH corrected Wilcoxon rank-sum test p-value < 0.05 and abs (log fold change) > 0.33. Here, igt = impaired glucose tolerance; ngt = normal glucose tolerance.

EC	t2d-ngt-FC	t2d-ngt-logFC	t2d-ngt-adj-p	igt-ngt-FC	igt-ngt-logFC	igt-ngt-adj-p
2.1.1.86	0.55	-0.87	0.043	0.79	-0.34	0.1944
2.4.1.8	1.52	0.6	0.0147	1.2	0.26	0.5785
2.4.1.5	5.43	2.44	0.0418	3.68	1.88	0.7413
1.4.1.24	0.57	-0.8	0.0131	0.83	-0.26	0.1445
1.4.1.1	1.35	0.43	0.0177	1.22	0.28	0.0895
3.1.4.16	0.79	-0.34	0.0133	0.77	-0.37	0.0268
2.5.1.41	0.46	-1.12	0.0481	0.74	-0.44	0.2051
1.5.99.15	0.76	-0.4	0.0494	0.86	-0.21	0.2767
3.4.21.62	1.37	0.45	0.0382	1.14	0.19	0.3887
2.1.1.90	0.52	-0.95	0.0202	0.93	-0.11	0.3653
2.1.1.98	0.47	-1.09	0.0104	0.56	-0.84	0.0409
3.2.1.80	1.8	0.85	0.0008	1.74	0.8	0.0008
2.5.1.120	6.25	2.64	0.0149	1.48	0.56	0.6458
1.1.1.385	1.88	0.91	0.0145	0.56	-0.83	0.5544
2.7.7.73	1.45	0.53	0.0003	0.96	-0.06	0.6726
2.7.7.72	0.48	-1.07	0.0428	0.38	-1.41	0.0059
5.3.2.8	1.56	0.64	0.0225	0.79	-0.35	0.8501
2.4.1.7	1.38	0.47	0.0125	1.26	0.34	0.1477
4.1.1.31	0.57	-0.82	0.0205	0.55	-0.88	0.0188
2.1.1.206	0.51	-0.98	0.0108	0.84	-0.25	0.2505
6.3.4.19	0.62	-0.69	0.0389	0.44	-1.19	0.0035

Supplementary Table S9. Significantly differentially abundant ECs identified by HUMAnN2 in the T2D-Karlsson data set. Significance thresholds used: BH corrected Wilcoxon rank-sum test p-value < 0.05 and abs (log fold change) > 0.33. Here, igt = impaired glucose tolerance; ngt = normal glucose tolerance.

EC	t2d-ngt FC	t2d-ngt logFC	t2d-ngt adj-p	igt-ngt FC	igt-ngt logFC	igt-ngt adj-p
1.2.7.8	0.59	-0.77	0.0294	0.74	-0.43	0.1691
2.5.1.86	0.29	-1.80	0.0186	0.28	-1.82	0.0318
5.1.2.1	1.38	0.46	0.0459	2.03	1.02	0.0210
4.1.1.101	5.34	2.42	0.0135	2.57	1.36	0.1014
3.5.4.27	0.60	-0.74	0.0082	1.02	0.03	0.2460
2.3.1.n4	2.54	1.34	0.0055	3.07	1.62	0.1079
2.7.14.1	0.31	-1.71	0.0319	0.81	-0.31	0.1862
2.3.1.136	3.69	1.88	0.0386	0.95	-0.08	0.9506
4.2.1.28	1.56	0.64	0.0075	0.89	-0.17	0.3897
5.1.1.13	0.69	-0.54	0.0317	0.30	-1.74	0.0003
2.7.1.121	0.35	-1.50	0.0142	0.70	-0.51	0.0028
2.7.4.8	0.69	-0.54	0.0298	0.51	-0.98	0.0011
3.5.1.5	1.55	0.63	0.0122	1.56	0.65	0.0705
3.1.3.15	0.09	-3.55	0.0402	0.76	-0.40	0.8988
2.7.1.205	0.52	-0.93	0.0025	0.45	-1.16	0.0019
4.1.2.57	0.37	-1.44	0.0330	0.37	-1.44	0.0661
1.12.98.2	0.71	-0.49	0.0232	1.28	0.36	0.4290
1.12.98.1	0.73	-0.46	0.0391	1.17	0.23	0.3172
1.1.1.87	0.34	-1.55	0.0076	0.93	-0.11	0.3338
2.8.3.12	4.77	2.25	0.0249	6.47	2.69	0.0148
4.1.99.17	1.59	0.67	0.0348	1.07	0.09	0.9407
1.5.1.36	39.64	5.31	0.0334	32.11	5.00	0.1246
6.1.1.11	0.78	-0.37	0.0314	0.85	-0.24	0.0783
1.3.7.11	0.56	-0.84	0.0144	1.28	0.36	0.9021
2.3.1.30	0.72	-0.47	0.0376	0.68	-0.55	0.0330
3.4.21.107	0.76	-0.40	0.0483	0.64	-0.65	0.0002
2.1.1.192	0.70	-0.51	0.0093	0.79	-0.35	0.0169
2.4.2.17	0.38	-1.40	0.0089	0.75	-0.41	0.2987
3.4.13.22	0.20	-2.29	0.0465	0.25	-2.03	0.0350
2.8.4.3	0.68	-0.56	0.0136	0.91	-0.13	0.2612
1.5.98.1	0.77	-0.39	0.0148	1.12	0.16	0.2677
6.2.1.3	0.65	-0.61	0.0164	0.71	-0.49	0.0301
2.4.1.5	6.11	2.61	0.0021	4.00	2.00	0.0077
1.8.1.8	0.71	-0.50	0.0422	0.40	-1.33	0.0001
3.2.1.80	4.15	2.05	0.0162	2.54	1.34	0.9552
4.3.1.3	0.51	-0.98	0.0160	0.77	-0.38	0.7561

Supplementary Table S9 (continued). Significantly differentially abundant ECs identified by HUMAnN2 in the T2D-Karlsson data set. Significance thresholds used: BH corrected Wilcoxon rank-sum test p-value < 0.05 and abs (log fold change) > 0.33. Here, igt = impaired glucose tolerance; ngt = normal glucose tolerance.

EC	t2d-ngt FC	t2d-ngt logFC	t2d-ngt adj-p	igt-ngt FC	igt-ng logFC	igt-ngt adj-p
2.7.1.6	0.73	-0.46	0.0309	1.05	0.08	0.9689
2.7.1.76	12.32	3.62	0.0372	12.04	3.59	0.0187
3.2.1.97	2.92	1.55	0.0063	3.28	1.71	0.4388
1.1.1.65	0.44	-1.17	0.0321	0.2	-2.33	0.003
2.4.1.7	1.85	0.89	0.0356	1.51	0.6	0.2126

Supplementary Table S10. Significantly differentially abundant ECs identified by Kraken2 in the T2D-Karlsson data set. Significance thresholds used: BH corrected Wilcoxon rank-sum test p-value < 0.05 and abs (log fold change) > 0.33. Here, igt = impaired glucose tolerance; ngt = normal glucose tolerance.

EC	t2d-ngt-FC	t2d-ngt-logFC	t2d-ngt-adj-p	igt-ngt-FC	igt-ngt-logFC	igt-ngt-adj-p
2.5.1.77	0.52	-0.94	0.0141	0.79	-0.35	0.1730
1.7.1.4	0.35	-1.52	0.0387	0.74	-0.44	0.1002
3.4.14.13	1.41	0.49	0.0463	1.26	0.34	0.3417
1.7.7.1	0.30	-1.72	0.0399	0.94	-0.10	0.4380
1.14.14.12	6.77	2.76	0.0040	6.37	2.67	0.0319
1.14.99.3	0.72	-0.48	0.0339	0.70	-0.51	0.0513
1.1.1.136	0.58	-0.79	0.0063	0.77	-0.37	0.0284
4.2.1.162	1.46	0.55	0.0020	1.17	0.22	0.0837
1.3.99.28	0.51	-0.98	0.0117	0.96	-0.06	0.0197
2.4.1.11	0.62	-0.69	0.0015	0.68	-0.57	0.0743
5.1.2.1	1.42	0.51	0.0147	1.47	0.56	0.1236
3.5.4.27	0.50	-1.00	0.0179	0.80	-0.31	0.2367
3.5.4.29	0.46	-1.12	0.0383	0.68	-0.56	0.2187
2.5.1.96	0.44	-1.19	0.0097	1.17	0.23	0.1779
2.1.1.63	0.64	-0.63	0.0272	0.48	-1.06	0.0005
4.1.1.65	0.60	-0.73	0.0169	0.36	-1.48	0.0139
4.2.1.36	1.40	0.49	0.0036	1.11	0.16	0.4157
2.4.2.48	0.74	-0.44	0.0299	1.02	0.03	0.7862
2.7.14.1	0.67	-0.57	0.0091	0.88	-0.19	0.2513
1.2.1.22	0.71	-0.50	0.0252	0.64	-0.64	0.0381
4.1.1.79	0.78	-0.36	0.0265	1.00	0.00	0.4826
6.3.4.21	0.70	-0.52	0.0384	0.62	-0.68	0.0058
2.4.2.4	0.67	-0.57	0.0030	0.80	-0.32	0.0634
1.3.4.1	0.55	-0.86	0.0453	0.82	-0.28	0.2245
1.2.1.38	1.33	0.42	0.0009	1.08	0.11	0.4543
3.5.1.5	1.41	0.50	0.0045	1.30	0.38	0.1005
6.3.4.12	2.20	1.14	0.0300	1.20	0.27	0.7834
3.6.4.9	0.55	-0.86	0.0213	0.95	-0.07	0.2528
3.2.1.91	1.75	0.81	0.0442	0.84	-0.25	0.6180
1.12.98.1	0.57	-0.80	0.0075	0.99	-0.02	0.7156
1.1.1.87	0.64	-0.65	0.0055	0.84	-0.26	0.3335
4.1.99.17	1.35	0.43	0.0009	1.04	0.06	0.8824
2.1.1.10	2.76	1.46	0.0064	1.82	0.87	0.2528
1.5.1.36	6.18	2.63	0.0066	5.67	2.50	0.1205
2.7.1.167	1.66	0.73	0.0098	1.17	0.22	0.6314
3.4.21.96	0.39	-1.34	0.0091	0.82	-0.28	0.0399
6.3.2.4	0.78	-0.35	0.0136	0.93	-0.11	0.2355

Supplementary Table S10 (continued). Significantly differentially abundant ECs identified by Kraken2 in the T2D-Karlsson data set. Significance thresholds used: BH corrected Wilcoxon rank-sum test p-value < 0.05 and abs (log fold change) > 0.33. Here, igt = impaired glucose tolerance; ngt = normal glucose tolerance.

EC	t2d-ngt FC	t2d-ngt logFC	t2d-ngt adj-p	igt-ngt FC	igt-ngt logFC	igt-ngt adj-p
1.12.7.2	0.71	-0.49	0.0133	0.86	-0.21	0.1157
3.1.3.70	0.52	-0.94	0.0370	0.33	-1.60	0.0112
2.7.1.175	2.64	1.40	0.0132	1.63	0.71	0.2943
1.16.3.1	0.53	-0.90	0.0385	0.24	-2.07	0.0057
1.1.1.333	0.11	-3.18	0.0128	0.04	-4.73	0.0019
2.7.4.26	0.49	-1.02	0.0028	0.73	-0.45	0.0740
2.1.1.198	0.67	-0.59	0.0106	0.73	-0.45	0.0214
3.4.13.22	0.52	-0.96	0.0492	0.60	-0.75	0.3439
2.8.4.1	0.62	-0.69	0.0045	0.92	-0.12	0.6181
1.2.99.7	1.52	0.60	0.0017	1.42	0.51	0.0055
2.4.1.9	1.88	0.91	0.0293	1.07	0.10	0.9686
3.4.11.10	0.28	-1.84	0.0105	0.54	-0.88	0.2528
4.1.2.17	0.50	-0.99	0.0131	0.41	-1.28	0.0331
2.7.7.1	0.47	-1.09	0.0189	0.75	-0.42	0.1802
2.7.7.47	0.62	-0.69	0.0433	0.08	-3.56	0.0066
1.5.98.1	0.49	-1.02	0.0077	0.83	-0.27	0.2805
3.8.1.7	0.50	-1.00	0.0032	0.89	-0.17	0.5086
4.2.3.153	0.54	-0.90	0.0078	0.92	-0.12	0.2880
6.2.1.3	0.72	-0.46	0.0398	0.67	-0.58	0.0046
3.1.1.48	3.69	1.88	0.0463	1.47	0.55	0.4962
1.14.13.154	4.89	2.29	0.0093	0.85	-0.23	0.6692
2.1.1.86	0.69	-0.54	0.0072	0.93	-0.11	0.5695
1.8.1.14	1.48	0.56	0.0030	1.29	0.36	0.0161
6.5.1.1	1.36	0.44	0.0060	1.66	0.73	0.0293
3.1.4.17	1.94	0.95	0.0386	1.40	0.49	0.2786
2.6.1.84	1.70	0.77	0.0054	1.52	0.61	0.3280
3.4.21.62	1.41	0.49	0.0215	1.15	0.20	0.4385
2.1.1.98	0.56	-0.84	0.0078	0.73	-0.45	0.1586
3.5.1.32	1.57	0.65	0.0126	1.11	0.15	0.7188
1.9.3.1	0.70	-0.52	0.0147	0.82	-0.28	0.2468
3.4.21.72	0.52	-0.94	0.0013	0.69	-0.53	0.0266
1.4.99.1	1.39	0.47	0.0125	0.80	-0.31	0.5855
1.4.99.5	3.41	1.77	0.0396	2.10	1.07	0.0861
2.5.1.113	1.30	0.38	0.0443	1.18	0.24	0.2233
1.13.11.5	3.00	1.58	0.0131	1.22	0.29	0.9321
3.5.1.110	0.40	-1.33	0.0142	0.32	-1.62	0.0030
3.4.11.1	1.54	0.63	0.0370	1.60	0.68	0.0731

Supplementary Table S10 (continued). Significantly differentially abundant ECs identified by Kraken2-translated search in the T2D-Karlsson data set. Significance thresholds used: BH corrected Wilcoxon rank-sum test p-value < 0.05 and abs (log fold change) > 0.33. Here, igt = impaired glucose tolerance; ngt = normal glucose tolerance.

EC	t2d-ngt-FC	t2d-ngt-logFC	t2d-ngt-adj-p	igt-ngt-FC	igt-ngt-logFC	igt-ngt-adj-p
4.2.1.148	3.35	1.74	0.0266	0.28	-1.84	0.8681
1.1.1.65	0.37	-1.45	0.0257	0.26	-1.93	0.0687
1.1.1.69	0.59	-0.77	0.0419	0.72	-0.48	0.0399
2.4.1.7	1.45	0.54	0.0224	1.30	0.37	0.0559
5.4.1.4	0.49	-1.02	0.0042	0.65	-0.62	0.0466
4.1.1.38	0.32	-1.65	0.0292	0.34	-1.57	0.0860

Supplementary Table S11. Pathways identified as significantly variable between T2D patients and healthy controls in the T2D-Qin data set using mi-faser-generated functional profiles. Significance thresholds used: BH corrected Wilcoxon rank-sum test p-value < 0.05 and abs (log fold change) > 0.11. Here, C = Carbohydrate Metabolism; L = Lipid Metabolism; E = Energy Metabolism; N = Nucleotide Metabolism; AA = Amino Acid Metabolism (includes metabolism of other amino acids as well); SM = Biosynthesis of Secondary Metabolites; G = Glycan Biosynthesis and Metabolism; V = Metabolism of Co-factors and Vitamins; X = Xenobiotics Biodegradation and Metabolism; GI = Genetic Information Processing; T = Metabolism of Terpenoids and Polyketides.

Category	ID	Name	fold-change	logFC	adj-pval
L	00061	Fatty acid biosynthesis	0.90	-0.15	0.000009
E	00195	Photosynthesis	0.86	-0.21	0.000456
SM	00254	Aflatoxin biosynthesis	0.85	-0.24	0.017628
AA	00472	D-Arginine and D-ornithine metabolism	1.16	0.21	0.003276
AA	00480	Glutathione metabolism	1.15	0.20	0.002753
G	00513	Various types of N-glycan biosynthesis	1.16	0.22	0.030179
T	00522	Biosynthesis of 12-, 14- and 16-membered macrolides	2.03	1.02	0.038230
G	00571	Lipoarabinomannan (LAM) biosynthesis	1.72	0.78	0.001068
L	00603	Glycosphingolipid biosynthesis - globo and isoglobo series	1.13	0.18	0.010605
X	00627	Aminobenzoate degradation	1.17	0.23	0.006568
V	00740	Riboflavin metabolism	0.91	-0.13	0.006686
V	00750	Vitamin B6 metabolism	0.86	-0.21	0.011154
V	00785	Lipoic acid metabolism	1.13	0.18	0.037381
T	00906	Carotenoid biosynthesis	1.54	0.62	0.000006
SM	00944	Flavone and flavonol biosynthesis	0.80	-0.33	0.043905
T	00981	Insect hormone biosynthesis	1.21	0.27	0.012885
SM	00999	Biosynthesis of secondary metabolites - unclassified	0.88	-0.19	0.001662
T	01051	Biosynthesis of ansamycins	0.84	-0.26	0.000619

Supplementary Table S12. Pathways identified as significantly variable between T2D patients and normal glucose tolerance (NGT) individuals in the T2D-Karlsson data set using mi-faser-generated functional profiles. Significance thresholds used: BH corrected Wilcoxon rank-sum test p-value < 0.05 and abs (log fold change) > 0.11. Here, C = Carbohydrate Metabolism; L = Lipid Metabolism; E = Energy Metabolism; N = Nucleotide Metabolism; AA = Amino Acid Metabolism (includes metabolism of other amino acids as well); SM = Biosynthesis of Secondary Metabolites; G = Glycan Biosynthesis and Metabolism; V = Metabolism of Co-factors and Vitamins; X = Xenobiotics Biodegradation and Metabolism; GI = Genetic Information Processing; T = Metabolism of Terpenoids and Polyketides.

Category	ID	Name	t2d-ngt FC	t2d-ngt logFC	t2d-ngt adj pval	igt-ngt FC	igt-ngt logFC	igt-ngt adj-pval
E	00190	Oxidative phosphorylation	1.09	0.12	0.0031	1.04	0.06	0.3296
E	00195	Photosynthesis	1.12	0.17	0.0215	1.09	0.12	0.3335
AA	00220	Arginine biosynthesis	1.09	0.12	0.0125	1.06	0.09	0.0756
SM	00261	Monobactam biosynthesis	1.08	0.11	0.0048	1.04	0.06	0.0756
AA	00290	Valine, leucine and isoleucine biosynthesis	1.08	0.11	0.0078	1.03	0.05	0.1817
AA	00400	Phenylalanine, tyrosine and tryptophan biosynthesis	1.10	0.14	0.0034	1.07	0.10	0.0501
AA	00460	Cyanoamino acid metabolism	1.12	0.16	0.0139	1.00	-0.01	0.6627
G	00511	Other glycan degradation	1.18	0.24	0.0082	1.11	0.16	0.5485
G	00531	Glycosaminoglycan degradation	1.14	0.19	0.0320	1.07	0.10	0.9689
L	00561	Glycerolipid metabolism	1.14	0.19	0.0002	1.08	0.11	0.0796
G	00572	Arabinogalactan biosynthesis - Mycobacterium	2.97	1.57	0.0069	1.77	0.82	0.1062
L	00600	Sphingolipid metabolism	1.16	0.22	0.0287	1.11	0.15	0.5909
L	00604	Glycosphingolipid biosynthesis - ganglio series	1.18	0.23	0.0101	1.11	0.15	0.5963
X	00791	Atrazine degradation	1.42	0.51	0.0048	1.29	0.37	0.0520

Supplementary Table S13. Pathways identified as significantly variable between T2D patients and healthy controls in the T2D-Qin data set using functional profiles generated by HUMAnN2. Significance thresholds used: BH corrected Wilcoxon rank-sum test p-value < 0.05 and abs (log fold change) > 0.11. Here, C = Carbohydrate Metabolism; L = Lipid Metabolism; E = Energy Metabolism; N = Nucleotide Metabolism; AA = Amino Acid Metabolism (includes metabolism of other amino acids as well); SM = Biosynthesis of Secondary Metabolites; G = Glycan Biosynthesis and Metabolism; V = Metabolism of Co-factors and Vitamins; X = Xenobiotics Biodegradation and Metabolism; GI = Genetic Information Processing; T = Metabolism of Terpenoids and Polyketides.

Category	ID	Name	fold-change	logFC	adj pval
C	00020	Citrate cycle (TCA cycle)	0.91	-0.13	0.0020
L	00072	Synthesis and degradation of ketone bodies	0.83	-0.28	0.0419
E	00190	Oxidative phosphorylation	0.88	-0.18	0.0145
E	00195	Photosynthesis	0.84	-0.25	0.0172
AA	00380	Tryptophan metabolism	1.11	0.15	0.0402
L	00561	Glycerolipid metabolism	1.08	0.12	0.0184
L	00603	Glycosphingolipid biosynthesis - globo and isoglobo series	1.11	0.15	0.0215
X	00623	Toluene degradation	3.32	1.73	0.0416
X	00633	Nitrotoluene degradation	0.91	-0.13	0.0312
V	00750	Vitamin B6 metabolism	0.81	-0.31	0.0039
T	00900	Terpenoid backbone biosynthesis	0.92	-0.12	0.0309
SM	00999	Biosynthesis of secondary metabolites - unclassified	0.80	-0.32	0.0386

Supplementary Table S14. Pathways identified as significantly variable between T2D patients and normal glucose tolerance (NGT) individuals in the T2D-Karlsson data set using functional profiles generated by HUMAnN2. Significance thresholds used: BH corrected Wilcoxon rank-sum test p-value < 0.05 and abs (log fold change) > 0.11. Here, C = Carbohydrate Metabolism; L = Lipid Metabolism; E = Energy Metabolism; N = Nucleotide Metabolism; AA = Amino Acid Metabolism (includes metabolism of other amino acids as well); SM = Biosynthesis of Secondary Metabolites; G = Glycan Biosynthesis and Metabolism; V = Metabolism of Co-factors and Vitamins; X = Xenobiotics Biodegradation and Metabolism; GI = Genetic Information Processing; T = Metabolism of Terpenoids and Polyketides.

Category	ID	Name	t2d-ngt FC	t2d-ngt logFC	t2d-ngt adj pval	igt-ngt FC	igt-ngt logFC	igt-ngt adj-pval
C	00051	Fructose and mannose metabolism	0.92	-0.12	0.0483	1.10	0.14	0.0180
L	00071	Fatty acid degradation	0.88	-0.18	0.0219	0.97	-0.05	0.6458
AA	00310	Lysine degradation	0.81	-0.30	0.0113	0.81	-0.31	0.0730
AA	00340	Histidine metabolism	0.66	-0.59	0.0019	0.79	-0.34	0.0348
AA	00460	Cyanoamino acid metabolism	1.24	0.32	0.0170	1.13	0.18	0.1089
V	00670	One carbon pool by folate	1.10	0.14	0.0371	1.07	0.09	0.5909
X	00791	Atrazine degradation	1.55	0.63	0.0122	1.56	0.65	0.0705

Supplementary Table S15. Pathways identified as significantly variable between T2D patients and healthy controls in the T2D-Qin data set using functional profiles generated by Kraken2. Significance thresholds used: BH corrected Wilcoxon rank-sum test p-value < 0.05 and abs (log fold change) > 0.11. Here, C = Carbohydrate Metabolism; L = Lipid Metabolism; E = Energy Metabolism; N = Nucleotide Metabolism; AA = Amino Acid Metabolism (includes metabolism of other amino acids as well); SM = Biosynthesis of Secondary Metabolites; G = Glycan Biosynthesis and Metabolism; V = Metabolism of Co-factors and Vitamins; X = Xenobiotics Biodegradation and Metabolism; GI = Genetic Information Processing; T = Metabolism of Terpenoids and Polyketides.

Category	ID	Name	fold-change	logFC	adj pval
L	00061	Fatty acid biosynthesis	1.14	0.19	0.0025
E	00195	Photosynthesis	0.87	-0.20	0.0026
SM	00254	Aflatoxin biosynthesis	0.83	-0.27	0.0088
AA	00380	Tryptophan metabolism	1.21	0.27	0.0021
SM	00401	Novobiocin biosynthesis	0.88	-0.19	0.0374
AA	00480	Glutathione metabolism	1.15	0.20	0.0034
G	00531	Glycosaminoglycan degradation	1.16	0.21	0.0232
L	00600	Sphingolipid metabolism	1.16	0.22	0.0086
L	00603	Glycosphingolipid biosynthesis - globo and isoglobo series	1.15	0.20	0.0243
V	00760	Nicotinate and nicotinamide metabolism	1.16	0.21	0.0051
L	01040	Biosynthesis of unsaturated fatty acids	0.83	-0.27	0.0017
T	01051	Biosynthesis of ansamycins	0.84	-0.25	0.0031

Supplementary Table S16. Pathways identified as significantly variable between T2D patients and normal glucose tolerance (NGT) individuals in the T2D-Karlsson data set using functional profiles generated by Kraken2. Significance thresholds used: BH corrected Wilcoxon rank-sum test p-value < 0.05 and abs (log fold change) > 0.11. Here, C = Carbohydrate Metabolism; L = Lipid Metabolism; E = Energy Metabolism; N = Nucleotide Metabolism; AA = Amino Acid Metabolism (includes metabolism of other amino acids as well); SM = Biosynthesis of Secondary Metabolites; G = Glycan Biosynthesis and Metabolism; V = Metabolism of Co-factors and Vitamins; X = Xenobiotics Biodegradation and Metabolism; GI = Genetic Information Processing; T = Metabolism of Terpenoids and Polyketides.

Category	ID	Name	t2d-ngt FC	t2d-ngt logFC	t2d-ngt adj pval	igt-ngt FC	igt-ngt logFC	igt-ngt adj-pval
C	00053	Ascorbate and aldarate metabolism	0.91	-0.13	0.0314	0.98	-0.03	0.5178
L	00072	Synthesis and degradation of ketone bodies	0.55	-0.86	0.0467	0.55	-0.86	0.0103
E	00190	Oxidative phosphorylation	1.11	0.15	0.0108	1.05	0.07	0.4175
E	00195	Photosynthesis	1.14	0.19	0.0180	1.06	0.09	0.5748
AA	00220	Arginine biosynthesis	1.09	0.12	0.0207	1.06	0.09	0.1038
SM	00254	Aflatoxin biosynthesis	1.13	0.18	0.0320	1.11	0.15	0.1355
SM	00261	Monobactam biosynthesis	1.08	0.11	0.0034	1.07	0.10	0.0612
X	00364	Fluorobenzoate degradation	0.67	-0.58	0.0236	1.18	0.24	0.6810
AA	00450	Selenocompound metabolism	1.13	0.18	0.0055	1.07	0.09	0.1107
G	00515	Mannose type O-glycan biosynthesis	0.83	-0.27	0.0357	1.11	0.15	0.5178
L	00561	Glycerolipid metabolism	1.11	0.15	0.0251	1.00	0.01	0.6402
X	00625	Chloroalkane and chloroalkene degradation	1.12	0.16	0.0042	1.11	0.14	0.0657
C	00660	C5-Branched dibasic acid metabolism	1.08	0.11	0.0125	1.06	0.08	0.0539
X	00791	Atrazine degradation	1.41	0.50	0.0043	1.30	0.37	0.1072
