# Supplementary material for: Carnelian uncovers hidden functional patterns across diverse study populations from whole metagenome sequencing reads
Source: Genome Biol. 2020 Feb 24;21:47. doi: 10.1186/s13059-020-1933-7 (PMC7038607; doi:10.1186/s13059-020-1933-7)
Supplement: Supplementary file 3 — Additional file 3 Results from Crohn’s Disease Cohorts. Contains Supplementary Tables S17–S32. [file 13059_2020_1933_MOESM3_ESM.pdf]

Carnelian uncovers hidden functional patterns across diverse study populations from whole metagenome sequencing reads

Sumaiya Nazeen[1], Yun William Yu[2], and Bonnie Berger[1,3*]

[1] Computer Science and Artificial Intelligence Laboratory (CSAIL), MIT, Cambridge, MA 02139, USA
[2] Department of Biomedical Informatics, HMS, Boston, MA 02115, USA
[3] Department of Mathematics, MIT, Cambridge, MA 02139, USA
* Corresponding Author: bab@mit.edu

Additional file 3 --- Results from Crohn's Disease Cohorts
Supplementary Tables S17-S32

Supplementary Table S17. Significantly differentially abundant ECs identified by Carnelian in the CD-HMP data set. Significance thresholds used: BH corrected Wilcoxon rank-sum test *p*-value < 0.05 and abs (log fold change) > 0.58.

EC	Fold Change	logFC	Adjusted p-value
2.4.1.292	0.63	-0.67	9.55E-05
1.10.3.10	0.66	-0.60	2.42E-03
2.7.7.39	1.73	0.79	2.42E-03
4.2.1.12	0.60	-0.75	2.61E-03
1.2.1.19	0.66	-0.60	4.31E-03
1.3.3.3	0.61	-0.71	6.07E-03
4.3.1.15	0.64	-0.64	6.07E-03
1.17.5.3	0.37	-1.44	8.42E-03
3.2.1.28	0.64	-0.65	8.98E-03
1.1.1.60	0.61	-0.72	1.23E-02
3.2.2.21	0.61	-0.71	1.23E-02
3.1.1.41	0.63	-0.67	1.65E-02
3.2.2.8	0.54	-0.88	1.65E-02
4.2.1.42	0.56	-0.84	1.75E-02
2.4.2.52	0.54	-0.89	1.86E-02
2.7.7.19	0.47	-1.08	2.08E-02
1.3.1.101	0.62	-0.69	2.20E-02
2.7.1.186	0.61	-0.72	2.20E-02
5.1.3.26	0.58	-0.79	2.20E-02
3.6.1.25	0.63	-0.68	2.33E-02
4.2.1.40	0.58	-0.79	2.60E-02
5.3.3.10	0.58	-0.78	2.65E-02
2.7.7.61	0.54	-0.89	3.05E-02
3.1.3.74	0.43	-1.22	3.05E-02
3.4.23.49	0.52	-0.93	3.05E-02
3.5.1.16	0.59	-0.76	3.39E-02
4.1.1.65	0.53	-0.93	3.57E-02
2.7.1.55	0.54	-0.88	3.96E-02
3.2.1.31	0.64	-0.65	3.96E-02
2.4.1.12	0.46	-1.13	4.17E-02
3.1.4.14	0.43	-1.20	4.60E-02
5.3.1.26	1.67	0.74	4.60E-02
1.17.1.9	0.54	-0.90	4.84E-02
1.8.5.5	0.59	-0.76	4.84E-02

Supplementary Table S18. Significantly differentially abundant ECs identified by Carnelian in the CD-Swedish data set. Significance thresholds used: BH corrected Wilcoxon rank-sum test p-value < 0.05 and abs (log fold change) > 0.58.

EC	Fold Change	logFC	Adjusted p-value	EC	Fold Change	logFC	Adjusted p-value
2.4.1.20	0.36	-1.46	1.00E-05	3.4.24.55	0.43	-1.23	5.46E-03
3.6.3.5	0.44	-1.19	1.23E-05	6.3.4.14	0.42	-1.25	5.84E-03
1.4.1.2	0.39	-1.35	3.53E-05	2.4.1.288	0.40	-1.32	6.12E-03
2.4.2.47	0.43	-1.20	3.87E-05	1.3.1.31	0.42	-1.26	6.20E-03
1.1.1.40	0.38	-1.41	4.64E-05	4.3.1.24	0.39	-1.35	6.21E-03
3.6.3.42	0.53	-0.93	4.64E-05	2.3.2.3	0.53	-0.92	6.26E-03
2.4.1.52	0.47	-1.08	5.08E-05	3.6.3.2	0.54	-0.88	6.99E-03
2.7.9.1	0.49	-1.04	5.55E-05	1.2.7.4	0.54	-0.90	7.38E-03
1.4.4.2	0.38	-1.39	6.62E-05	1.2.99.7	0.45	-1.14	7.56E-03
3.4.24.69	0.40	-1.31	7.22E-05	1.14.13.171	0.31	-1.71	7.57E-03
2.4.1.25	0.50	-1.00	1.81E-04	4.1.2.27	0.30	-1.74	8.32E-03
3.4.21.53	0.50	-1.00	1.81E-04	6.1.1.10	0.65	-0.61	8.67E-03
5.4.99.2	0.41	-1.27	1.81E-04	4.6.1.1	0.54	-0.89	1.02E-02
1.8.98.3	0.42	-1.26	2.29E-04	1.2.7.5	0.57	-0.80	1.07E-02
3.2.1.3	0.53	-0.91	2.34E-04	2.4.1.247	0.39	-1.35	1.12E-02
2.4.2.46	0.38	-1.40	2.48E-04	6.3.5.4	0.62	-0.69	1.19E-02
3.2.1.21	0.45	-1.16	2.48E-04	2.7.8.47	0.35	-1.50	1.36E-02
3.2.1.14	0.51	-0.97	4.21E-04	6.2.1.51	0.56	-0.84	1.36E-02
2.3.2.27	0.45	-1.16	5.25E-04	3.2.1.4	0.47	-1.10	1.38E-02
4.2.1.135	0.45	-1.15	5.41E-04	2.4.1.19	0.39	-1.35	1.56E-02
3.2.1.18	0.39	-1.37	6.51E-04	2.4.99.21	0.59	-0.77	1.60E-02
2.4.2.48	0.36	-1.47	9.38E-04	3.2.1.52	0.58	-0.80	1.60E-02
5.99.1.2	0.61	-0.72	9.88E-04	1.13.11.61	0.45	-1.15	1.94E-02
3.2.1.133	0.43	-1.23	1.53E-03	3.2.1.176	0.35	-1.51	1.97E-02
3.1.26.12	0.46	-1.13	1.79E-03	2.7.1.195	0.63	-0.66	2.51E-02
3.2.1.169	0.51	-0.98	1.95E-03	3.1.11.5	0.60	-0.73	2.57E-02
3.2.1.131	0.64	-0.64	2.11E-03	4.2.1.9	0.65	-0.62	2.57E-02
3.1.7.2	0.50	-1.01	2.18E-03	3.1.21.3	0.46	-1.11	2.80E-02
1.4.7.1	0.63	-0.67	2.45E-03	1.2.7.6	0.44	-1.19	2.97E-02
1.1.1.39	0.34	-1.54	2.50E-03	1.2.4.2	0.67	-0.59	3.05E-02
3.6.3.4	0.55	-0.86	2.77E-03	2.7.1.193	0.58	-0.79	3.05E-02
4.1.1.32	0.34	-1.55	2.80E-03	4.1.99.17	0.60	-0.74	3.21E-02
3.2.1.41	0.40	-1.31	3.05E-03	1.8.7.1	0.40	-1.31	3.47E-02
6.1.1.18	0.59	-0.76	3.13E-03	3.4.21.72	0.63	-0.68	3.53E-02
2.4.1.9	0.42	-1.26	3.15E-03	1.7.7.1	0.47	-1.10	3.58E-02
3.2.1.35	0.47	-1.08	3.48E-03	3.2.1.8	0.59	-0.77	3.82E-02
4.1.1.18	0.47	-1.10	3.68E-03	3.2.1.1	0.55	-0.87	3.84E-02
2.2.1.7	0.57	-0.81	3.97E-03	5.4.99.15	0.58	-0.78	3.92E-02
2.3.1.41	0.54	-0.89	3.97E-03	3.2.1.187	2.63	1.39	4.91E-02
3.2.1.177	0.55	-0.87	4.46E-03	4.2.1.82	0.52	-0.95	4.97E-02
1.8.5.5	0.46	-1.13	5.44E-03				

Supplementary Table S19. Pathways identified as significantly variable between CD patients and healthy controls in the CD-HMP data set using Carnelian-generated functional profiles. Significance thresholds used: BH corrected Wilcoxon rank-sum test p-value < 0.05 and absolute logFC >= 0.11. Here, C = Carbohydrate Metabolism; L = Lipid Metabolism; E = Energy Metabolism; N = Nucleotide Metabolism; AA = Amino Acid Metabolism (includes metabolism of other amino acids as well); SM = Biosynthesis of Secondary Metabolites; G = Glycan Biosynthesis and Metabolism; V = Metabolism of Co-factors and Vitamins; X = Xenobiotics Biodegradation and Metabolism; GI = Genetic Information Processing; T = Metabolism of Terpenoids and Polyketides.

Category	PathID	Name	Fold Change	logFC	Adjusted p-value
GI	00970	Aminoacyl-tRNA biosynthesis	0.93	-0.11	4.02E-03
AA	00300	Lysine biosynthesis	0.89	-0.17	7.90E-03
C	00620	Pyruvate metabolism	0.89	-0.17	1.23E-02
L	00561	Glycerolipid metabolism	1.17	0.23	1.30E-02
AA	00290	Valine, leucine and isoleucine biosynthesis	0.89	-0.18	2.33E-02
AA	00460	Cyanoamino acid metabolism	0.90	-0.15	2.60E-02
AA	00450	Selenocompound metabolism	0.88	-0.18	2.89E-02
C	00640	Propanoate metabolism	0.88	-0.18	3.05E-02
C	00500	Starch and sucrose metabolism	0.90	-0.15	3.39E-02
X	00930	Caprolactam degradation	1.19	0.25	1.56E-02
SM	00944	Flavone and flavonol biosynthesis	1.57	0.65	3.96E-02
T	00523	Polyketide sugar unit biosynthesis	0.81	-0.31	4.17E-02
SM	00521	Streptomycin biosynthesis	0.82	-0.28	4.17E-02
L	00062	Fatty acid elongation	1.19	0.25	4.17E-02
G	00513	Various types of N-glycan biosynthesis	1.18	0.24	4.38E-02
T	00281	Geraniol degradation	1.20	0.27	1.65E-02
T	01051	Biosynthesis of ansamycins	1.16	0.21	1.75E-02
T	01053	Biosynthesis of siderophore group nonribosomal peptides	1.25	0.33	3.57E-02
T	00981	Insect hormone biosynthesis	1.32	0.41	1.66E-03
X	00627	Aminobenzoate degradation	1.17	0.22	2.81E-03
T	00903	Limonene and pinene degradation	1.19	0.26	6.49E-03
E	00710	Carbon fixation in photosynthetic organisms	0.93	-0.11	1.08E-02
E	00195	Photosynthesis	0.89	-0.17	1.15E-02
GI	ko02030	Bacterial chemotaxis	1.11	0.15	1.38E-02
GI	ko02040	Flageller assembly	0.86	-0.21	7.40E-03

Supplementary Table S20. Pathways identified as significantly variable between CD patients and healthy controls in the CD-Swedish data set using Carnelian-generated functional profiles. Significance thresholds used: BH corrected Wilcoxon rank-sum test p-value < 0.05 and absolute logFC >= 0.11. Here, C = Carbohydrate Metabolism; L = Lipid Metabolism; E = Energy Metabolism; N = Nucleotide Metabolism; AA = Amino Acid Metabolism (includes metabolism of other amino acids as well); SM = Biosynthesis of Secondary Metabolites; G = Glycan Biosynthesis and Metabolism; V = Metabolism of Co-factors and Vitamins; X = Xenobiotics Biodegradation and Metabolism; GI = Genetic Information Processing; T = Metabolism of Terpenoids and Polyketides.

Category	PathID	Name	Fold_Change	logFC	Adjusted p-value
C	00010	Glycolysis / Gluconeogenesis	0.58	-0.79	2.45E-03
C	00620	Pyruvate metabolism	0.64	-0.65	2.95E-03
AA	00260	Glycine, serine and threonine metabolism	0.40	-1.33	4.24E-05
C	00500	Starch and sucrose metabolism	0.56	-0.83	9.04E-06
C	00520	Amino sugar and nucleotide sugar metabolism	0.72	-0.48	9.64E-03
V	00730	Thiamine metabolism	0.59	-0.76	1.45E-02
AA	00220	Arginine biosynthesis	0.39	-1.35	3.53E-05
AA	00280	Valine, leucine and isoleucine degradation	0.41	-1.27	1.81E-04
AA	00330	Arginine and proline metabolism	0.54	-0.88	1.81E-04
AA	00460	Cyanoamino acid metabolism	0.45	-1.16	2.48E-04
AA	00310	Lysine degradation	0.52	-0.94	2.89E-04
AA	00290	Valine, leucine and isoleucine biosynthesis	0.65	-0.62	2.57E-02
C	00640	Propanoate metabolism	0.68	-0.56	3.82E-02
AA	00450	Selenocompound metabolism	0.73	-0.46	3.98E-02
L	00600	Sphingolipid metabolism	0.79	-0.35	4.91E-02
L	00061	Fatty acid biosynthesis	0.48	-1.07	3.12E-04
C	00630	Glyoxylate and dicarboxylate metabolism	0.54	-0.89	3.63E-04
C	00040	Pentose and glucuronate interconversions	0.54	-0.88	1.57E-02
L	00603	Glycosphingolipid biosynthesis - globo and isoglobo series	0.58	-0.80	1.60E-02
G	00513	Various types of N-glycan biosynthesis	0.58	-0.80	1.60E-02
E	00920	Sulfur metabolism	0.51	-0.98	9.64E-03
E	00910	Nitrogen metabolism	0.57	-0.82	1.36E-05
AA	00360	Phenylalanine metabolism	0.51	-0.98	1.74E-06
C	00770	Pantothenate and CoA biosynthesis	0.65	-0.62	2.57E-02
SM	00960	Tropane, piperidine and pyridine alkaloid biosynthesis	0.50	-0.99	8.04E-04
X	00633	Nitrotoluene degradation	0.53	-0.91	2.03E-03
E	00710	Carbon fixation in photosynthetic organisms	0.54	-0.89	6.62E-05
G	00571	Lipoarabinomannan (LAM) biosynthesis	0.43	-1.20	3.87E-05
G	00572	Arabinogalactan biosynthesis - Mycobacterium	0.40	-1.31	4.51E-08
AA	00430	Taurine and hypotaurine metabolism	0.40	-1.32	2.76E-06
SM	00940	Phenylpropanoid biosynthesis	0.47	-1.09	4.79E-06
E	00720	Carbon fixation pathways in prokaryotes	0.57	-0.80	2.89E-04
V	00780	Biotin metabolism	0.54	-0.89	3.97E-03
T	00900	Terpenoid backbone biosynthesis	0.57	-0.81	3.97E-03
SM	00999	Biosynthesis of secondary metabolites - unclassified	0.31	-1.71	7.57E-03

Supplementary Table S21. Significantly differentially abundant ECs identified by mi-faser in the CD-HMP data set. Significance thresholds used: BH corrected Wilcoxon rank-sum test p-value < 0.05 and abs (log fold change) > 0.58.

EC	Fold Change	logFC	Adjusted p-value
2.3.1.n3	0.67	-0.58	1.21E-03
2.1.1.113	0.16	-2.64	1.02E-02
4.2.3.152	0.17	-2.52	1.23E-02
3.2.1.28	1.88	0.91	1.75E-02
4.2.1.51	0.05	-4.20	2.08E-02
3.4.19.5	2.00	1.00	2.08E-02
2.7.7.2	0.08	-3.62	2.33E-02
1.1.1.108	0.08	-3.59	2.33E-02
2.3.1.169	0.42	-1.25	2.37E-02
1.1.1.310	0.55	-0.85	2.65E-02
1.18.1.3	1.72	0.78	2.65E-02
3.2.1.156	0.57	-0.80	2.89E-02
6.3.1.13	0.17	-2.52	2.92E-02
3.9.1.2	0.64	-0.65	2.93E-02
3.2.1.70	0.59	-0.75	3.22E-02
2.3.1.41	1.50	0.59	3.22E-02
1.17.2.1	3.55	1.83	3.25E-02
1.2.7.4	0.58	-0.78	4.62E-02
4.2.2.2	0.24	-2.03	4.67E-02
2.7.7.39	0.57	-0.81	4.80E-02

Supplementary Table S22. Significantly differentially abundant ECs identified by mi-faser in the CD-Swedish data set. Significance thresholds used: BH corrected Wilcoxon rank-sum test p-value < 0.05 and abs (log fold change) > 0.58.

EC	Fold Change	logFC	Adjusted p-value	EC	Fold Change	logFC	Adjusted p-value
1.4.4.2	0.44	-1.17	3.53E-05	2.4.1.247	0.13	-2.91	1.12E-02
4.1.1.31	3.04	1.61	9.03E-05	3.2.1.135	2.81	1.49	1.24E-02
3.2.1.11	25.42	4.67	9.89E-05	1.17.4.2	217.55	7.77	1.39E-02
2.7.1.195	7.17	2.84	1.10E-04	3.1.7.2	1.87	0.90	1.46E-02
3.2.1.4	0.26	-1.93	1.21E-04	3.6.3.8	1.56	0.64	1.60E-02
1.17.4.1	2.41	1.27	1.42E-04	2.7.1.197	1.88	0.91	1.68E-02
2.4.1.20	0.48	-1.07	1.54E-04	2.1.1.13	0.54	-0.90	1.72E-02
3.2.1.18	9.24	3.21	1.83E-04	3.2.1.3	0.51	-0.98	1.82E-02
2.3.2.3	10.57	3.40	6.38E-04	4.1.1.38	10.11	3.34	1.92E-02
3.2.1.97	4.46	2.16	6.51E-04	2.4.1.279	0.12	-3.08	2.52E-02
1.1.98.6	2.45	1.29	8.04E-04	6.2.1.1	0.32	-1.64	2.67E-02
3.2.1.187	12.87	3.69	1.30E-03	2.7.1.207	4.54	2.18	2.68E-02
3.4.21.96	9.66	3.27	1.82E-03	3.2.1.41	0.63	-0.68	2.81E-02
5.4.99.2	0.53	-0.92	3.88E-03	4.2.1.162	2.05	1.03	3.18E-02
1.1.5.12	1.78	0.83	4.82E-03	3.4.24.70	0.51	-0.97	3.24E-02
3.4.14.12	0.47	-1.09	5.64E-03	1.97.1.2	0.12	-3.06	3.39E-02
3.2.1.68	3.47	1.80	5.82E-03	3.4.11.2	6.24	2.64	3.59E-02
3.2.1.8	0.39	-1.36	6.40E-03	3.6.3.4	1.90	0.93	3.60E-02
4.1.1.32	0.27	-1.89	7.20E-03	4.2.2.8	0.67	-0.58	3.99E-02
1.2.3.3	7.14	2.84	7.58E-03	4.2.1.135	0.63	-0.67	4.52E-02
1.2.4.1	3.37	1.75	7.76E-03	1.4.3.21	3.62	1.86	4.89E-02
6.2.1.36	6.44	2.69	1.09E-02	2.4.1.8	8.05	3.01	4.94E-02

Supplementary Table S23. Pathways identified as significantly variable between CD patients and healthy controls in the CD-HMP data set using mi-faser-generated functional profiles. Significance thresholds used: BH corrected Wilcoxon rank-sum test p-value < 0.05 and absolute logFC >= 0.11. Here, L = Lipid Metabolism; AA = Amino Acid Metabolism (includes metabolism of other amino acids as well); X = Xenobiotics Biodegradation and Metabolism; T = Metabolism of Terpenoids and Polyketides.

Category	PathId	Name	Fold Change	logFC	Adjusted p-value
AA	00471	D-Glutamine and D-glutamate metabolism	1.26	0.33	1.38E-02
L	00561	Glycerolipid metabolism	1.21	0.27	1.86E-02
T	00281	Geraniol degradation	1.35	0.43	2.89E-02
L	00062	Fatty acid elongation	1.35	0.43	3.05E-02
X	00930	Caprolactam degradation	1.42	0.51	3.39E-02

Supplementary Table S24. Pathways identified as significantly variable between CD patients and healthy controls in the CD-Swedish data set using mi-faser-generated functional profiles. Significance thresholds used: BH corrected Wilcoxon rank-sum test p-value < 0.05 and absolute logFC >= 0.11. Here, C = Carbohydrate Metabolism; L = Lipid Metabolism; E = Energy Metabolism; N = Nucleotide Metabolism; AA = Amino Acid Metabolism (includes metabolism of other amino acids as well); SM = Biosynthesis of Secondary Metabolites; G = Glycan Biosynthesis and Metabolism; V = Metabolism of Co-factors and Vitamins; X = Xenobiotics Biodegradation and Metabolism; GI = Genetic Information Processing; T = Metabolism of Terpenoids and Polyketides.

Category	PathId	Name	Fold Change	logFC	Adjusted p-value
AA	00260	Glycine, serine and threonine metabolism	0.46	-1.12	5.08E-05
AA	00480	Glutathione metabolism	2.35	1.23	7.22E-05
X	00983	Drug metabolism - other enzymes	1.93	0.95	3.12E-04
V	00770	Pantothenate and CoA biosynthesis	0.70	-0.52	2.16E-03
AA	00290	Valine, leucine and isoleucine biosynthesis	0.70	-0.52	2.16E-03
AA	00280	Valine, leucine and isoleucine degradation	0.53	-0.92	3.88E-03
V	00730	Thiamine metabolism	0.74	-0.43	5.92E-03
T	00900	Terpenoid backbone biosynthesis	0.74	-0.44	1.38E-02
C	00520	Amino sugar and nucleotide sugar metabolism	0.86	-0.23	1.94E-02
C	00052	Galactose metabolism	1.49	0.58	2.24E-02
	01051	Biosynthesis of ansamycins	1.35	0.43	2.45E-02
C	00020	Citrate cycle (TCA cycle)	1.43	0.51	3.35E-02
AA	00410	beta-Alanine metabolism	3.62	1.86	4.89E-02
AA	00350	Tyrosine metabolism	3.62	1.86	4.89E-02
SM	00950	Isoquinoline alkaloid biosynthesis	3.62	1.86	4.89E-02

Supplementary Table S25. Significantly differentially abundant ECs identified by HUMAnN2 in the CD-HMP data set. Significance thresholds used: BH corrected Wilcoxon rank-sum test p-value < 0.05 and abs (log fold change) > 0.58.

EC	Fold Change	logFC	Adjusted p-value
3.5.1.104	0.25	-2.00	3.33E-03
2.4.1.329	0.01	-7.09	3.93E-03
2.7.8.36	0.66	-0.61	4.91E-02
3.2.1.37	0.63	-0.66	4.95E-02
4.2.2.26	0.00	-9.15	2.08E-02
1.18.1.3	0.66	-0.59	2.39E-02
2.3.1.35	0.23	-2.11	4.31E-02
4.2.1.77	0.10	-3.34	3.19E-03
4.1.1.96	0.33	-1.60	1.42E-02
2.7.1.162	0.34	-1.58	2.24E-02
2.6.1.57	0.36	-1.46	8.29E-03
3.2.1.70	0.47	-1.10	2.69E-02
3.2.1.151	0.50	-1.01	1.77E-02
6.3.1.12	0.15	-2.76	4.87E-02
2.4.99.16	0.24	-2.07	4.49E-02
3.6.3.40	0.57	-0.80	4.05E-02
1.3.1.n3	0.63	-0.67	1.36E-02
2.4.1.342	0.20	-2.29	2.61E-02
2.3.1.89	0.30	-1.74	3.14E-02
1.1.1.3	0.40	-1.31	1.19E-02

Supplementary Table S26. Significantly differentially abundant ECs identified by HUMAnN2 in the CD-Swedish data set. Significance thresholds used: BH corrected Wilcoxon rank-sum test p-value < 0.05 and abs (log fold change) > 0.58.

EC	Fold Change	logFC	Adjusted p-value
1.17.4.1	2.29	1.19	7.92E-03
4.2.1.53	1.77	0.83	4.89E-02
2.4.1.12	2.08	1.06	2.18E-02
3.2.1.187	5.32	2.41	6.36E-03
3.2.1.185	4.21	2.07	1.01E-02
3.2.1.20	3.10	1.63	3.78E-03
3.2.1.21	0.37	-1.42	1.09E-02
3.2.1.28	0.44	-1.19	4.77E-02
2.2.1.9	10.82	3.44	4.59E-03
6.1.1.19	2.01	1.01	3.03E-02
6.1.1.5	2.38	1.25	2.86E-02
3.6.3.8	2.69	1.43	3.89E-02
1.8.4.13	2.65	1.41	1.98E-02
2.3.3.9	2.76	1.46	9.31E-03
6.2.1.1	0.55	-0.87	3.84E-02
2.4.1.8	24.52	4.62	2.23E-03
2.4.1.5	3.96	1.98	2.24E-02
1.4.1.2	0.33	-1.59	3.65E-04
1.2.5.1	10.10	3.34	6.86E-03
6.3.5.5	1.67	0.74	1.53E-02
1.2.4.2	2.48	1.31	4.35E-02
1.3.5.4	3.15	1.65	3.98E-02
2.4.1.211	2.59	1.38	3.13E-03
3.2.1.170	1.60	0.68	2.61E-02
4.1.1.31	4.35	2.12	9.31E-03

Supplementary Table S27. Pathways identified as significantly variable between CD patients and healthy controls in the CD-HMP data set using HUMAnN2-generated functional profiles. Significance thresholds used: BH corrected Wilcoxon rank-sum test p-value < 0.05 and absolute logFC >= 0.11. Here, L = Lipid Metabolism; V = Metabolism of Co-factors and Vitamins; X = Xenobiotics Biodegradation and Metabolism;

Category	Path ID	Name	Fold Change	logFC	Adjusted p-value
X	01055	Biosynthesis of vancomycin group antibiotics	0.72	-0.47	1.65E-02
X	00624	Polycyclic aromatic hydrocarbon degradation	0.69	-0.54	3.56E-02
V	00670	One carbon pool by folate	0.92	-0.13	3.76E-02
L	00561	Glycerolipid metabolism	1.27	0.34	4.84E-02

Supplementary Table S28. Pathways identified as significantly variable between CD patients and healthy controls in the CD-Swedish data set using HUMAnN2-generated functional profiles. Significance thresholds used: BH corrected Wilcoxon rank-sum test p-value < 0.05 and absolute logFC >= 0.11. Here, C = Carbohydrate Metabolism; L = Lipid Metabolism; E = Energy Metabolism; N = Nucleotide Metabolism; AA = Amino Acid Metabolism (includes metabolism of other amino acids as well); SM = Biosynthesis of Secondary Metabolites; G = Glycan Biosynthesis and Metabolism; V = Metabolism of Co-factors and Vitamins; X = Xenobiotics Biodegradation and Metabolism; GI = Genetic Information Processing; T = Metabolism of Terpenoids and Polyketides.

Category	Path ID	Name	Fold Change	logFC	Adjusted p-value
AA	00220	Arginine biosynthesis	0.33	-1.59	3.65E-04
AA	00430	Taurine and hypotaurine metabolism	0.33	-1.59	3.65E-04
AA	00330	Arginine and proline metabolism	0.41	-1.27	1.81E-03
AA	00480	Glutathione metabolism	2.16	1.11	4.42E-03
C	00500	Starch and sucrose metabolism	0.51	-0.98	4.55E-03
V	00130	Ubiquinone and other terpenoid-quinone biosynthesis	10.82	3.44	4.59E-03
GI	ko02020	Two-component system	0.71	-0.49	7.79E-03
AA	00460	Cyanoamino acid metabolism	0.37	-1.42	1.09E-02
C	00040	Pentose and glucuronate interconversions	1.95	0.96	1.56E-02
SM	00940	Phenylpropanoid biosynthesis	0.39	-1.36	2.43E-02
X	00983	Drug metabolism - other enzymes	1.77	0.82	3.44E-02
C	00630	Glyoxylate and dicarboxylate metabolism	1.20	0.26	3.46E-02
L	00061	Fatty acid biosynthesis	5.55	2.47	3.98E-02
E	00910	Nitrogen metabolism	0.56	-0.84	4.71E-02

Supplementary Table S29. Significantly differentially abundant ECs identified by Kraken2 in the CD-HMP data set. Significance thresholds used: BH corrected Wilcoxon rank-sum test p-value < 0.05 and abs (log fold change) > 0.58.

EC	Fold Change	logFC	Adjusted p-value
2.4.1.161	0.34	-1.54	2.76E-02
2.1.1.44	0.27	-1.89	1.89E-02
1.2.3.3	0.37	-1.43	4.77E-02
1.14.13.70	0.19	-2.36	4.49E-02
1.3.99.28	0.29	-1.77	4.21E-02
3.2.1.183	0.26	-1.97	1.24E-02
1.3.1.86	0.11	-3.18	4.23E-03
3.2.2.26	0.43	-1.21	1.43E-02
3.2.1.28	1.83	0.87	2.46E-02
1.11.2.4	0.45	-1.14	5.23E-03
2.7.4.6	1.55	0.64	2.42E-03
3.2.1.31	1.61	0.69	2.60E-02
3.2.1.37	0.62	-0.70	2.38E-02
3.2.1.35	0.32	-1.66	9.80E-03
1.5.1.43	0.38	-1.39	4.49E-02
2.4.1.109	0.07	-3.89	2.33E-02
5.5.1.25	11.28	3.50	4.44E-02
1.14.13.92	0.30	-1.74	1.02E-02
3.1.4.3	0.47	-1.09	2.10E-02
1.14.14.5	1.97	0.98	6.07E-03
6.3.1.20	1.72	0.79	1.65E-02
2.7.7.43	3.25	1.70	2.54E-02
3.5.4.9	0.41	-1.27	1.84E-02
2.8.1.15	0.23	-2.12	1.15E-02
2.4.1.15	1.50	0.59	2.42E-03
4.2.3.155	0.37	-1.43	7.85E-03
1.14.13.154	7.32	2.87	3.81E-02
6.3.5.11	0.48	-1.06	1.87E-02
3.2.1.156	0.47	-1.09	1.30E-02
3.1.1.74	0.59	-0.76	4.34E-02
1.1.1.374	0.36	-1.48	2.45E-02
2.7.7.62	0.50	-0.99	2.11E-02
1.3.5.4	1.64	0.71	1.75E-02
4.1.1.1	0.15	-2.75	2.25E-02

Supplementary Table S30. Significantly differentially abundant ECs identified by Kraken2 in the CD-Swedish data set. Significance thresholds used: BH corrected Wilcoxon rank-sum test p-value < 0.05 and abs (log fold change) > 0.58.

EC	Fold Change	logFC	Adjusted p-value	EC	Fold Change	logFC	Adjusted p-value
6.3.5.1	0.48	-1.05	2.01E-05	3.2.1.141	2.81	1.49	8.36E-03
4.1.1.31	2.65	1.41	3.32E-05	3.2.1.20	2.13	1.09	9.19E-03
3.2.1.97	5.73	2.52	4.43E-05	4.1.1.38	7.13	2.83	1.13E-02
2.4.1.20	0.39	-1.34	4.43E-05	3.2.1.4	0.67	-0.58	1.25E-02
2.4.1.279	0.03	-5.14	4.48E-05	4.2.2.1	5.38	2.43	1.34E-02
2.7.1.197	3.00	1.58	5.07E-04	4.2.2.24	0.45	-1.17	1.55E-02
6.3.5.3	3.23	1.69	8.29E-04	3.2.1.135	6.21	2.64	1.78E-02
3.2.1.185	5.89	2.56	1.22E-03	1.17.4.2	168.40	7.40	1.80E-02
3.2.1.187	12.51	3.64	1.46E-03	3.2.1.170	4.11	2.04	1.80E-02
1.1.1.40	0.56	-0.83	1.91E-03	3.2.1.177	0.61	-0.72	1.94E-02
2.2.1.7	0.66	-0.59	2.95E-03	3.2.1.1	0.56	-0.84	1.97E-02
2.6.1.97	6.48	2.70	3.02E-03	3.4.24.68	1.81	0.85	2.03E-02
3.2.1.11	2.05	1.04	3.14E-03	1.4.3.21	4.40	2.14	2.50E-02
1.1.5.12	0.22	-2.17	3.39E-03	3.2.1.131	0.65	-0.62	2.72E-02
1.2.3.3	5.20	2.38	4.32E-03	4.2.2.23	0.29	-1.77	2.83E-02
2.4.1.247	0.37	-1.42	4.76E-03	1.1.1.39	0.62	-0.70	2.98E-02
1.1.98.6	2.21	1.14	4.80E-03	3.2.1.21	0.66	-0.61	4.33E-02
1.8.7.1	4.58	2.19	4.82E-03	1.2.4.1	1.67	0.74	4.41E-02
3.6.3.8	1.71	0.77	5.29E-03	4.2.1.3	1.89	0.92	4.71E-02
2.2.1.1	1.66	0.73	6.26E-03	2.4.1.288	3.71	1.89	4.89E-02
4.3.1.24	3.55	1.83	6.40E-03	4.1.1.32	0.23	-2.10	4.90E-02
5.4.99.2	0.52	-0.93	6.54E-03				

Supplementary Table S31. Pathways identified as significantly variable between CD patients and healthy controls in the CD-HMP data set using Kraken2-generated functional profiles. Significance thresholds used: BH corrected Wilcoxon rank-sum test p-value < 0.05 and absolute logFC >= 0.11. Here, C = Carbohydrate Metabolism; L = Lipid Metabolism; E = Energy Metabolism; N = Nucleotide Metabolism; AA = Amino Acid Metabolism (includes metabolism of other amino acids as well); SM = Biosynthesis of Secondary Metabolites; G = Glycan Biosynthesis and Metabolism; V = Metabolism of Co-factors and Vitamins; X = Xenobiotics Biodegradation and Metabolism; GI = Genetic Information Processing; T = Metabolism of Terpenoids and Polyketides.

Category	Path ID	Name	Fold Change	logFC	Adjusted p-value
T	00522	Biosynthesis of 12-, 14- and 16-membered macrolides	4.75	2.25	1.13E-02
AA	00400	Phenylalanine, tyrosine and tryptophan biosynthesis	0.91	-0.13	1.15E-02
L	00565	Ether lipid metabolism	0.36	-1.46	1.25E-02
X	00361	Chlorocyclohexane and chlorobenzene degradation	2.53	1.34	2.20E-02
SM	00405	Phenazine biosynthesis	0.86	-0.22	2.20E-02
SM	00944	Flavone and flavonol biosynthesis	1.61	0.69	2.60E-02
AA	00410	beta-Alanine metabolism	0.92	-0.11	2.60E-02
GI	00970	Aminoacyl-tRNA biosynthesis	0.92	-0.11	3.39E-02

Supplementary Table S32. Pathways identified as significantly variable between CD patients and healthy controls in the CD-Swedish data set using Kraken2-generated functional profiles. Significance thresholds used: BH corrected Wilcoxon rank-sum test p-value < 0.05 and absolute logFC >= 0.11. Here, C = Carbohydrate Metabolism; L = Lipid Metabolism; E = Energy Metabolism; N = Nucleotide Metabolism; AA = Amino Acid Metabolism (includes metabolism of other amino acids as well); SM = Biosynthesis of Secondary Metabolites; G = Glycan Biosynthesis and Metabolism; V = Metabolism of Co-factors and Vitamins; X = Xenobiotics Biodegradation and Metabolism; GI = Genetic Information Processing; T = Metabolism of Terpenoids and Polyketides.

Category	Path ID	Name	Fold Change	logFC	Adjusted p-value
T	00900	Terpenoid backbone biosynthesis	0.66	-0.59	2.95E-03
T	01051	Biosynthesis of ansamycins	1.66	0.73	6.26E-03
AA	00280	Valine, leucine and isoleucine degradation	0.52	-0.93	6.54E-03
C	ko02060	Phosphotransferase system (PTS)	1.42	0.50	7.79E-03
C	00030	Pentose phosphate pathway	1.59	0.67	1.02E-02
C	00500	Starch and sucrose metabolism	0.83	-0.27	1.52E-02
C	00520	Amino sugar and nucleotide sugar metabolism	0.83	-0.27	1.60E-02
C	00770	Pantothenate and CoA biosynthesis	0.76	-0.40	2.45E-02
AA	00290	Valine, leucine and isoleucine biosynthesis	0.76	-0.40	2.45E-02
AA	00410	beta-Alanine metabolism	4.40	2.14	2.50E-02
AA	00350	Tyrosine metabolism	4.40	2.14	2.50E-02
SM	00950	Isoquinoline alkaloid biosynthesis	4.40	2.14	2.50E-02
AA	00260	Glycine, serine and threonine metabolism	4.40	2.14	2.50E-02
E	00710	Carbon fixation in photosynthetic organisms	0.85	-0.23	2.57E-02
V	00730	Thiamine metabolism	0.75	-0.41	2.94E-02
AA	00460	Cyanoamino acid metabolism	0.66	-0.61	4.33E-02
C	00620	Pyruvate metabolism	0.88	-0.19	4.33E-02
