# Supplementary material for: Carnelian uncovers hidden functional patterns across diverse study populations from whole metagenome sequencing reads
Source: Genome Biol. 2020 Feb 24;21:47. doi: 10.1186/s13059-020-1933-7 (PMC7038607; doi:10.1186/s13059-020-1933-7)
Supplement: Supplementary file 4 — Additional file 4 Supplementary Figures. Contains Supplementary Figures S1–S7. [file 13059_2020_1933_MOESM4_ESM.pdf]

Carnelian uncovers hidden functional patterns across diverse study populations from whole metagenome sequencing reads

Sumaiya Nazeen[1], Yun William Yu[2], and Bonnie Berger[1,3*]

[1] Computer Science and Artificial Intelligence Laboratory (CSAIL), MIT, Cambridge, MA 02139, USA
[2] Department of Biomedical Informatics, HMS, Boston, MA 02115, USA
[3] Department of Mathematics, MIT, Cambridge, MA 02139, USA
* Corresponding Author: bab@mit.edu

Additional file 4 --- Supplementary Figures S1 – S7

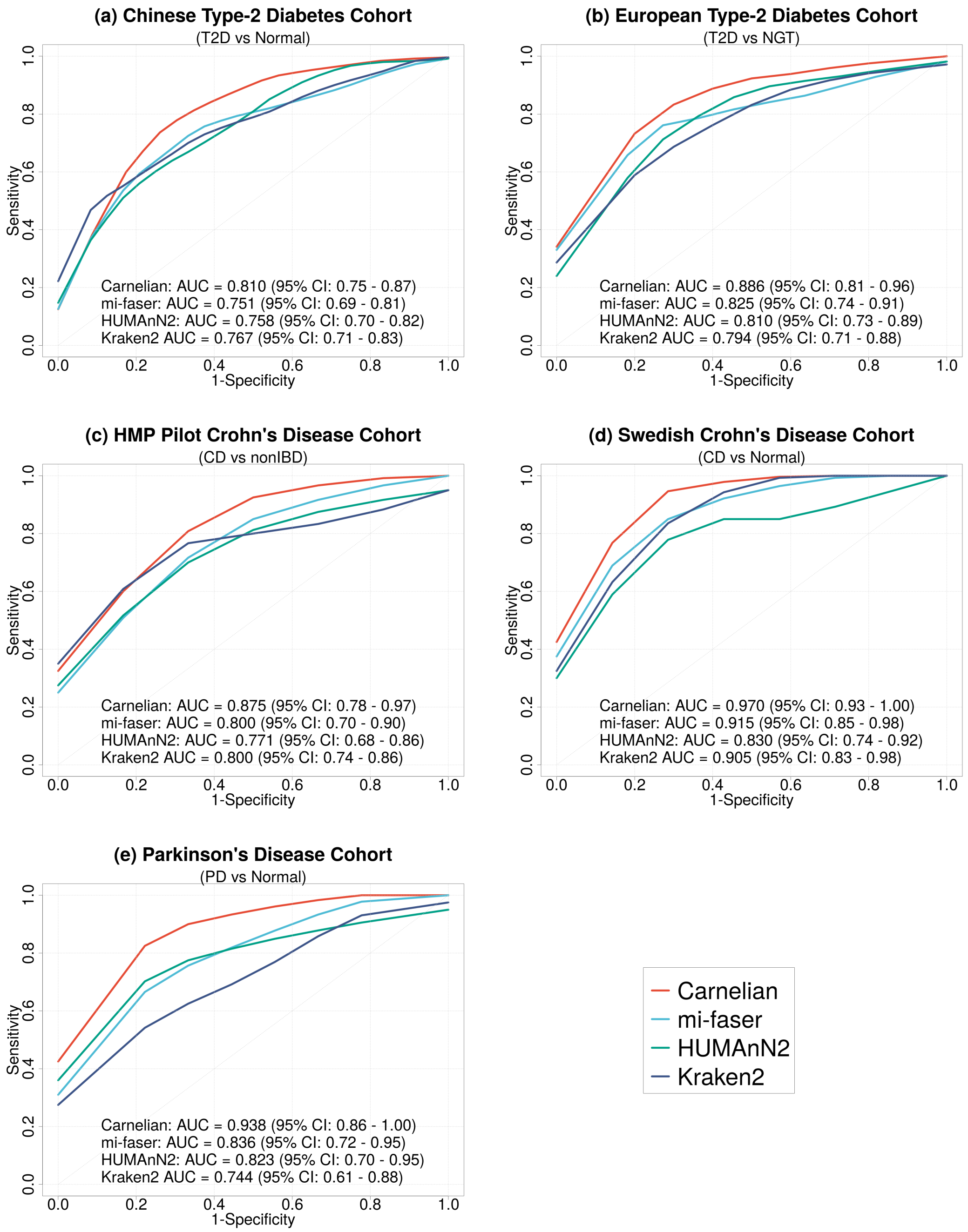

Supplementary Figure S1. Classification of patients vs. controls using Enzyme Commission (EC) markers (*N*-fold cross-validation experiments). (a) T2D vs controls in the T2D-Qin data set (Chinese cohort); **(b)** T2D vs. Normal Glucose Tolerance (NGT) individuals in the T2D-Karlsson data set (European cohort). **(c)** CD patients vs. controls in the CD-HMP data set (individuals from the US). **(d)** CD patients vs. healthy individuals in the CD-Swedish data set (Swedish twin studies). **(e)** PD vs controls in the PD-Bedarf data set. Differentially abundant ECs were selected from the entire data set as features input to a set of random forest classifiers. Average area under the curve (AUC) over all cross-validation trials is reported as a measure of accuracy. Carnelian-identified EC markers achieve a larger area under the curve (AUC) in all the cases compared to those identified by mi-faser, HUMAnN2, and Kraken2.

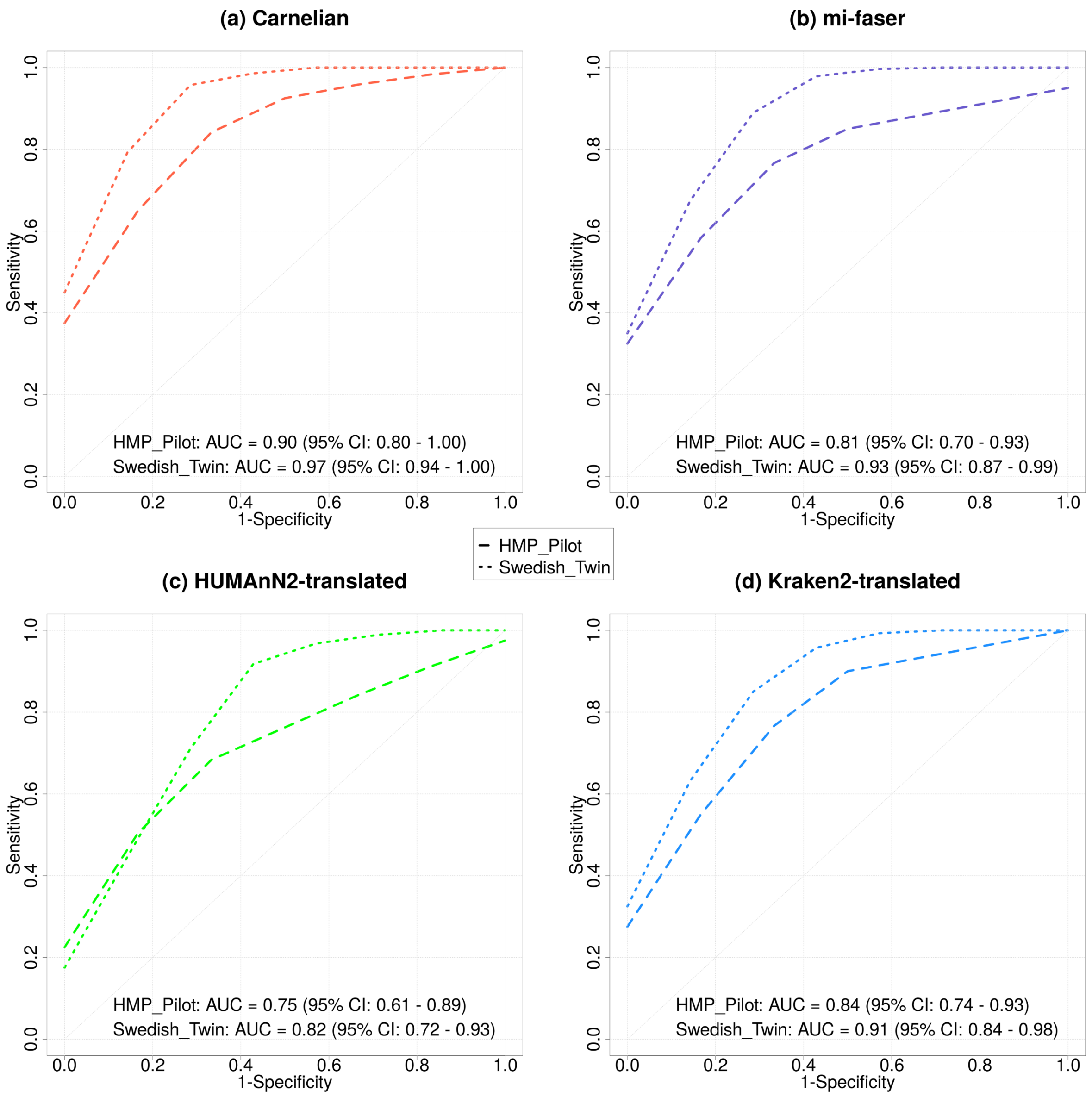

Supplementary Figure S2. Classification of patients vs. controls in Crohn's disease cohorts from the US and Sweden using the combined set of markers identified by Carnelian, mi-faser, HUMAnN2-translated, and Kraken2-translated. Using Carnelian-identified highly variable ECs, we can consistently achieve >0.90 area under the curve (AUC) on average on both CD-HMP (HMP Pilot phase data from IBDMDB) and CD-Swedish (Swedish Twin Cohort data from NCBI SRP002423) data sets which is higher than the other three tools.

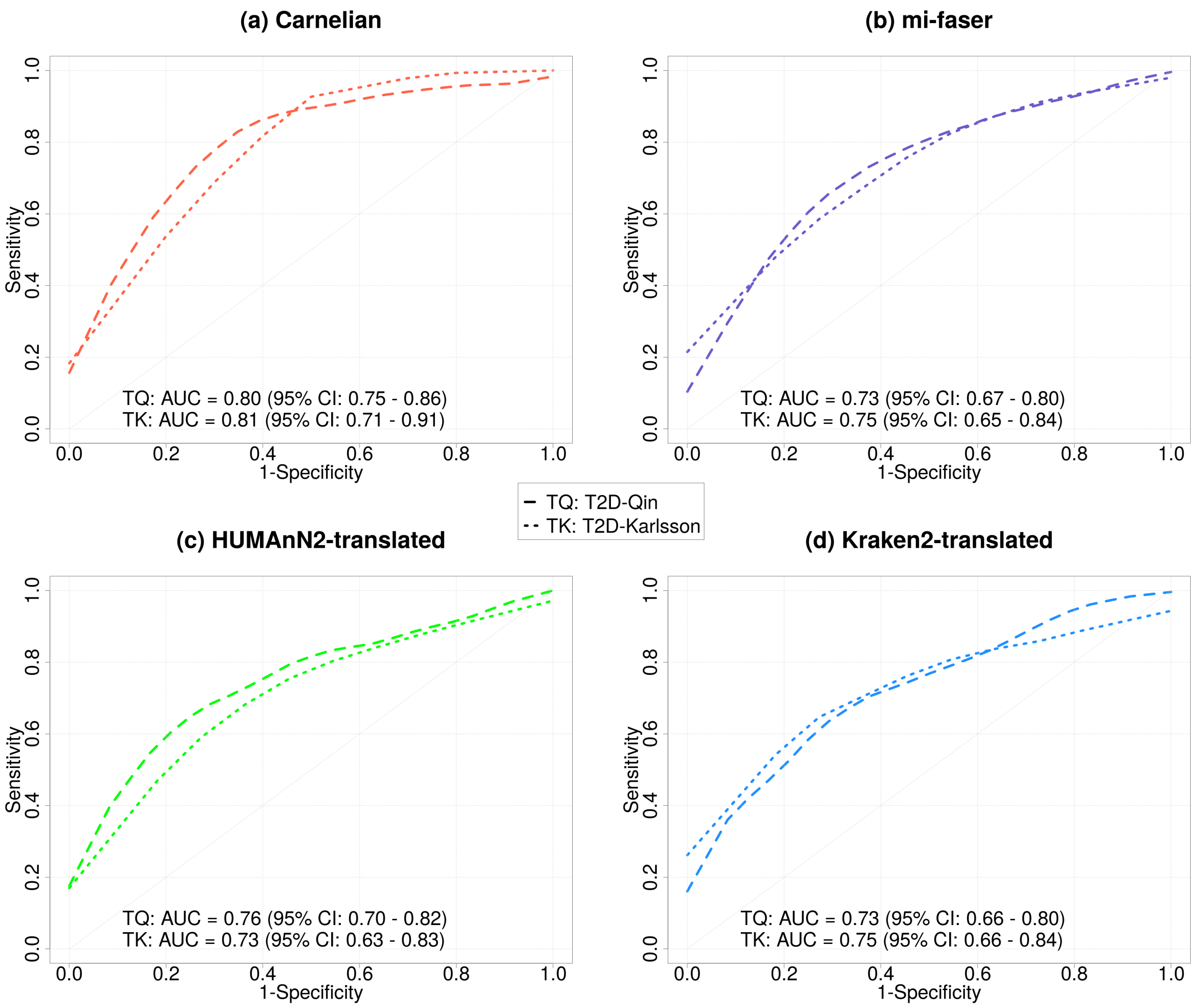

Supplementary Figure S3. Classification of patients vs. controls in Chinese and European T2D cohorts using the combined set of markers identified by Carnelian, mi-faser, HUMAnN2-translated, and Kraken2-translated. Using Carnelian-identified highly variable ECs, we can consistently achieve ~0.80 area under the curve (AUC) on average on both T2D-Qin and T2D-Karlsson data sets, whereas using the highly variable ECs identified by other methods, an average AUC of 0.73-0.76 can be achieved on both data sets.

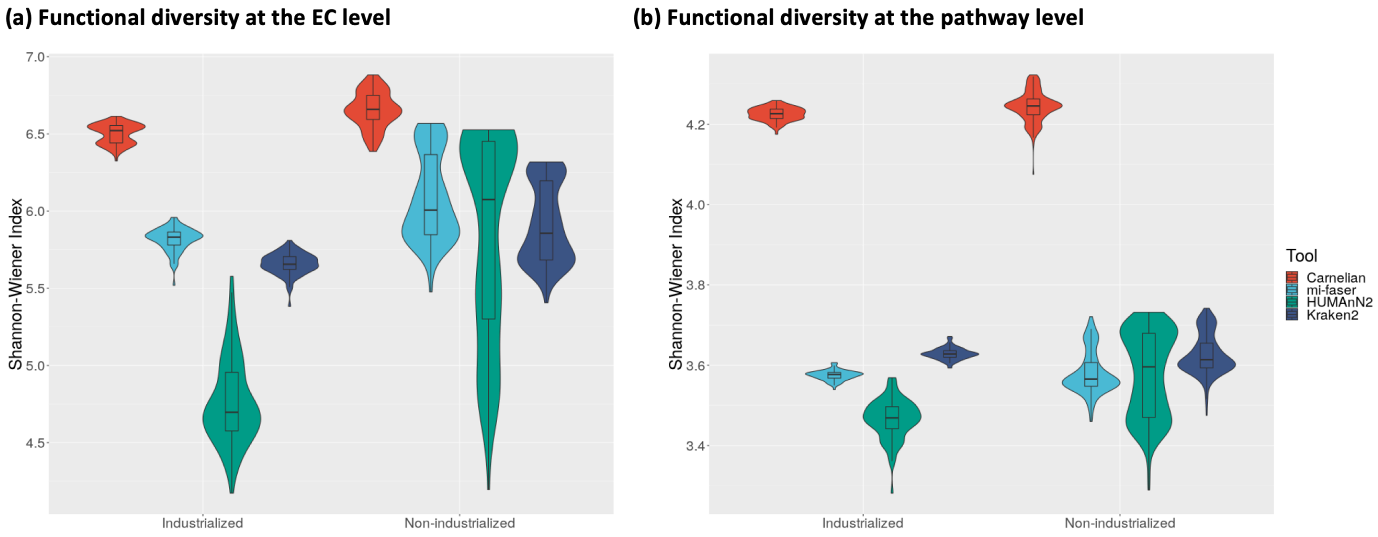

Supplementary Figure S4. Functional diversity observed in industrialized and non-industrialized microbiomes. (a) Diversity at the EC-level. **(b)** Diversity at the pathway level. Carnelian identifies more diversity at both the levels than other methods as indicated by Shanon-Wiener indices.

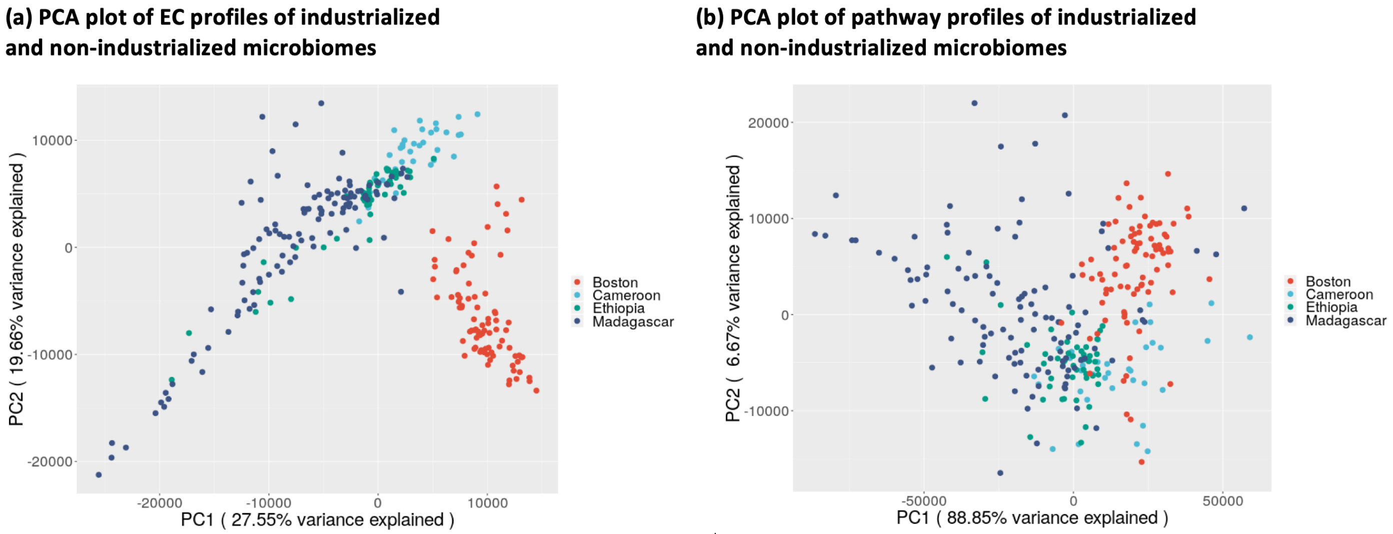

Supplementary Figure S5. Principal component analysis (PCA) plot depicting the Carnelian-derived functional profiles of industrialized and non-industrialized gut microbiomes. (a) PCA plot of EC profiles show separation of populations. **(b)** Pathway profiles of the microbiomes do not show much separation indicating a high degree of similarity between the microbial metabolic functionalities of industrialized and non-industrialized communities.

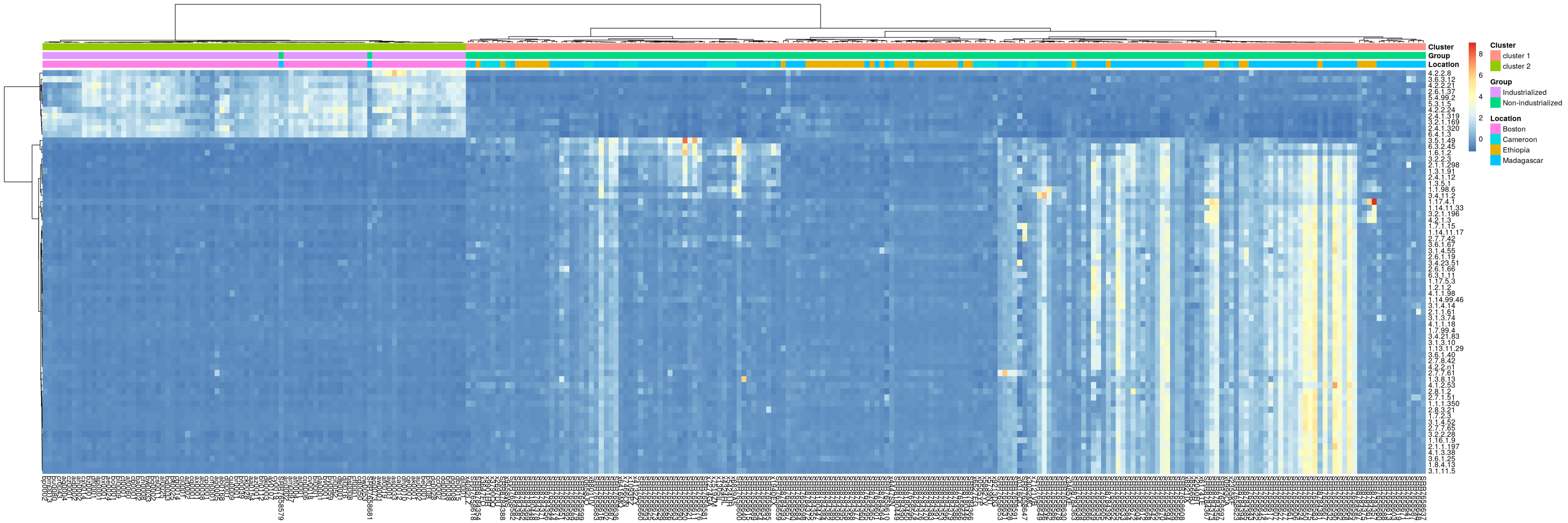

Supplementary Figure S6. Heatmap showing the z-scores of read abundances in ECs with high weights in the top principal components. Ward-linkage hierarchical clustering of the EC profiles (with highly weighted ECs in the PCA) of industrialized and non-industrialized microbiomes was performed using Pearson correlation. The two clusters found by hierarchical clustering capture the separation of non-industrialized and industrialized microbiomes perfectly except for the two outlier samples from Madagascar: SRR7658579 and SRR7658681.

k-mer	N	L	G	T	L	E	P	W	L	Hash
Hash Functions	1	1	1	0	0	0	0	0	0	NLG
	0	0	0	1	1	1	0	0	0	TLE
	0	0	0	0	0	0	1	1	1	PWL
	1	0	0	0	1	0	0	1	0	NLW
	0	1	0	0	0	1	1	0	0	LEP
	0	0	1	1	0	0	0	0	1	GTL
Coverage	2	2	2	2	2	2	2	2	2	

Supplementary Figure S7. Example of low-density even coverage hashing representation of an amino acid *k*-mer. A (*k, r*)-hash function can be thought of as a binary vector of length *k* with *r* 1's where each 1 indicates a marked position in the *k*-mer. Leveraging the hashing technique from Opal, Carnelian starts with a set of hash functions, represented as a hash matrix where the first row has 1's in first *r* positions, the second row has 1's in second *r* positions, and so on. *Carnelian* then permutes the columns of this matrix repeatedly to generate even coverage LSH functions. The rows then give the corresponding hashes of a *k*-mer.
