# Supplementary material for: Carnelian uncovers hidden functional patterns across diverse study populations from whole metagenome sequencing reads
Source: Genome Biol. 2020 Feb 24;21:47. doi: 10.1186/s13059-020-1933-7 (PMC7038607; doi:10.1186/s13059-020-1933-7)
Supplement: Supplementary file 5 — Additional file 5 Results from Case-Control Classification and Functional Relatedness of Healthy Microbiomes. Contains Supplementary Tables S33–S37 and S47. [file 13059_2020_1933_MOESM5_ESM.pdf]

# Carnelian uncovers hidden functional patterns across diverse study populations from whole metagenome sequencing reads

Sumaiya Nazeen[1], Yun William Yu[2], and Bonnie Berger[1,3*]

[1] Computer Science and Artificial Intelligence Laboratory (CSAIL), MIT, Cambridge, MA 02139, USA
[2] Department of Biomedical Informatics, HMS, Boston, MA 02115, USA
[3] Department of Mathematics, MIT, Cambridge, MA 02139, USA
* Corresponding Author: bab@mit.edu

## Additional file 5 --- Results from Case-Control Classification and Functional Relatedness of Healthy Microbiomes Supplementary Tables S33-S37 and S47

**Supplementary Table S33.** Combined EC markers identified by Carnelian that can classify T2D patients vs. controls in both Chinese and European population with ~80% area under the curve on average.

| | | | | |
|---|---|---|---|---|
| 5.4.99.62 | 1.17.7.4 | 4.2.1.147 | 3.1.3.12 | 2.8.4.1 |
| 3.6.1.23 | 1.17.7.3 | 2.4.1.7 | 1.12.98.1 | 1.2.99.7 |
| 2.4.2.2 | 1.8.2.3 | 5.4.3.2 | 6.2.1.44 | 2.4.1.15 |
| 2.4.2.6 | 1.1.1.28 | 3.1.3.85 | 2.3.2.21 | 6.2.1.3 |
| 1.1.1.100 | 3.2.1.52 | 2.4.1.329 | 5.4.99.20 | 4.2.1.119 |
| 2.7.8.35 | 2.4.1.1 | 4.1.1.101 | 2.1.1.10 | 3.4.13.9 |
| 3.7.1.8 | 1.4.1.24 | 4.2.99.20 | 5.3.1.22 | 3.1.4.16 |
| 2.7.2.4 | 1.4.1.4 | 3.1.3.8 | 1.5.1.36 | 3.1.4.12 |
| 1.13.11.27 | 2.6.1.84 | 2.6.1.113 | 4.2.1.42 | 2.1.1.90 |
| 4.1.1.33 | 2.7.7.61 | 3.2.2.23 | 1.1.1.251 | 3.2.1.80 |
| 4.2.1.20 | 4.2.1.120 | 4.2.2.n2 | 4.1.2.48 | 2.4.1.182 |
| 2.7.1.220 | 5.4.2.11 | 4.1.1.79 | 6.3.2.33 | 1.13.11.3 |
| 1.1.1.408 | 4.3.1.15 | 1.14.13.127 | 1.8.98.1 | 6.3.2.36 |
| 1.12.2.1 | 1.3.1.70 | 3.5.1.5 | 2.3.1.5 | 1.13.11.6 |
| 1.8.4.14 | | | | |

**Supplementary Table S34.** Combined EC markers identified by Carnelian that can classify CD patients vs. controls in both the US and Swedish population with ~94% area under the curve on average.

| | | | | |
|---|---|---|---|---|
| 2.4.1.292 | 3.1.3.74 | 5.4.99.2 | 3.2.1.35 | 3.2.1.4 |
| 1.10.3.10 | 3.4.23.49 | 1.8.98.3 | 4.1.1.18 | 2.4.1.19 |
| 2.7.7.39 | 3.5.1.16 | 3.2.1.3 | 2.2.1.7 | 2.4.99.21 |
| 4.2.1.12 | 4.1.1.65 | 2.4.2.46 | 2.3.1.41 | 3.2.1.52 |
| 1.2.1.19 | 2.7.1.55 | 3.2.1.21 | 3.2.1.177 | 1.13.11.61 |
| 1.3.3.3 | 3.2.1.31 | 3.2.1.14 | 3.4.24.55 | 3.2.1.176 |
| 4.3.1.15 | 2.4.1.12 | 2.3.2.27 | 6.3.4.14 | 2.7.1.195 |
| 1.17.5.3 | 3.1.4.14 | 4.2.1.135 | 2.4.1.288 | 3.1.11.5 |
| 3.2.1.28 | 5.3.1.26 | 3.2.1.18 | 1.3.1.31 | 4.2.1.9 |
| 1.1.1.60 | 1.17.1.9 | 2.4.2.48 | 4.3.1.24 | 3.1.21.3 |
| 3.2.2.21 | 1.8.5.5 | 5.99.1.2 | 2.3.2.3 | 1.2.7.6 |
| 3.1.1.41 | 2.4.1.20 | 3.2.1.133 | 3.6.3.2 | 1.2.4.2 |
| 3.2.2.8 | 3.6.3.5 | 3.1.26.12 | 1.2.7.4 | 2.7.1.193 |
| 4.2.1.42 | 1.4.1.2 | 3.2.1.169 | 1.2.99.7 | 4.1.99.17 |
| 2.4.2.52 | 2.4.2.47 | 3.2.1.131 | 1.14.13.171 | 1.8.7.1 |
| 2.7.7.19 | 1.1.1.40 | 3.1.7.2 | 4.1.2.27 | 3.4.21.72 |
| 1.3.1.101 | 3.6.3.42 | 1.4.7.1 | 6.1.1.10 | 1.7.7.1 |
| 2.7.1.186 | 2.4.1.52 | 1.1.1.39 | 4.6.1.1 | 3.2.1.8 |
| 5.1.3.26 | 2.7.9.1 | 3.6.3.4 | 1.2.7.5 | 3.2.1.1 |
| 3.6.1.25 | 1.4.4.2 | 4.1.1.32 | 2.4.1.247 | 5.4.99.15 |
| 4.2.1.40 | 3.4.24.69 | 3.2.1.41 | 6.3.5.4 | 3.2.1.187 |
| 5.3.3.10 | 2.4.1.25 | 6.1.1.18 | 2.7.8.47 | 4.2.1.82 |
| 2.7.7.61 | 3.4.21.53 | 2.4.1.9 | 6.2.1.51 | |

**Supplementary Table S35.** Performance of Carnelian, mi-faser, HUMAnN2, and Kraken2 on population data sets from Boston, Cameroon, Ethiopia, and Madagascar. Carnelian annotates significantly more reads and identifies more ECs compared to mi-faser, HUMAnN2, and Kraken2 in all four datasets.

| | | Boston 84 individuals | Cameroon 35 individuals | Ethiopia 50 individuals | Madagascar 112 individuals | Industrialized (B) | Non-industrialized (CEM) |
|---|---|---|---|---|---|---|---|
| Carnelian | # Annotated reads per sample | 1,430,026 | 269,720 | 2,182,173 | 2,157,741 | 1,430,026 | 1,828,507 |
| | # ECs per sample | 1981 | 2003 | 2002 | 2003 | 1981 | 2003 |
| mi-faser | # Annotated reads per sample | 743,877 | 268,684 | 1,181,031 | 1,527,111 | 743,877 | 1,215,695 |
| | # ECs per sample | 1230 | 1252 | 1309 | 1368 | 1230 | 1310 |
| HUMAnN2 | # Annotated reads per sample | 83,131 | 21,383 | 281,640 | 819,424 | 83,131 | 541,147 |
| | # ECs per sample | 791 | 827* | 919 | 1064 | 791 | 937 |
| Kraken2 | # Annotated reads per sample | 466,709 | 149,191 | 709,543 | 1,046,199 | 466,709 | 801,387 |
| | # ECs per sample | 1238 | 1219 | 1280 | 1233 | 1238 | 1244 |

* Running out-of-the-box HUMAnN2 (using ChocoPhlAn, Uniref, and MetaCyc databases) on the individuals of Cameroon another 125 ECs could be detected in addition to the 827, but did not change the overall results much.

**Supplementary Table S36.** Weights of top 52 original EC terms in the first nine principal components which cumulatively explains ~80% variance in the principal component analysis of the EC profiles of industrialized and non-industrialized gut microbiome. These ECs are mostly involved in microbial carbohydrate, amino acid, nucleotide, and energy metabolism pathways.

| EC | PC1 | PC2 | PC3 | PC4 | PC5 | PC6 | PC7 | PC8 | PC9 |
|---|---|---|---|---|---|---|---|---|---|
| 4.1.1.11 | 0.19 | 0.17 | 0.04 | 0.31 | -0.25 | 0.03 | -0.08 | 0.24 | -0.09 |
| 5.1.3.32 | -0.18 | -0.14 | -0.08 | 0.15 | 0.02 | -0.15 | 0.18 | 0.05 | -0.10 |
| 2.7.7.24 | 0.16 | 0.10 | -0.12 | 0.24 | 0.06 | -0.02 | 0.12 | -0.19 | 0.01 |
| 6.4.1.3 | 0.15 | -0.11 | 0.05 | 0.01 | 0.01 | 0.00 | -0.02 | -0.03 | -0.02 |
| 1.11.1.15 | 0.15 | -0.28 | 0.10 | -0.05 | -0.03 | -0.01 | -0.02 | -0.04 | -0.17 |
| 1.16.3.2 | 0.15 | -0.17 | 0.08 | -0.03 | -0.04 | -0.04 | -0.06 | 0.07 | -0.07 |
| 4.6.1.12 | 0.15 | 0.10 | -0.04 | -0.03 | -0.07 | -0.06 | 0.04 | 0.19 | 0.01 |
| 2.7.1.11 | 0.14 | -0.08 | 0.01 | -0.03 | 0.00 | -0.03 | -0.03 | 0.06 | 0.02 |
| 1.1.1.100 | 0.13 | 0.02 | -0.03 | -0.02 | 0.02 | -0.01 | -0.02 | 0.09 | 0.03 |
| 2.5.1.47 | 0.13 | 0.00 | -0.03 | -0.02 | -0.01 | -0.02 | 0.04 | 0.09 | 0.02 |
| 1.7.1.15 | -0.13 | -0.08 | -0.04 | 0.07 | -0.01 | -0.07 | 0.10 | 0.01 | -0.08 |
| 2.6.1.83 | 0.13 | 0.02 | 0.00 | 0.07 | 0.08 | -0.02 | 0.00 | 0.05 | 0.04 |
| 4.2.1.11 | 0.12 | 0.02 | -0.02 | -0.02 | -0.01 | -0.04 | 0.03 | -0.01 | -0.09 |
| 5.1.3.13 | 0.12 | 0.03 | -0.04 | 0.09 | 0.04 | 0.00 | 0.00 | -0.04 | 0.05 |
| 3.2.1.3 | 0.12 | -0.04 | 0.01 | 0.12 | 0.01 | 0.00 | 0.00 | -0.06 | 0.02 |
| 5.2.1.8 | -0.11 | -0.02 | -0.03 | 0.06 | -0.03 | -0.03 | -0.02 | 0.00 | 0.09 |
| 6.3.5.2 | 0.10 | 0.00 | -0.02 | -0.05 | -0.01 | -0.01 | 0.02 | 0.00 | 0.01 |
| 5.4.99.18 | 0.10 | 0.00 | -0.01 | 0.00 | 0.05 | 0.01 | 0.02 | -0.03 | 0.00 |
| 3.2.1.21 | 0.10 | -0.07 | 0.03 | 0.02 | 0.03 | 0.02 | -0.07 | -0.04 | 0.05 |
| 3.1.4.52 | -0.10 | -0.07 | -0.04 | 0.05 | 0.00 | -0.06 | 0.07 | 0.01 | -0.02 |
| 4.2.1.47 | 0.10 | -0.06 | 0.02 | 0.04 | 0.01 | -0.02 | -0.01 | 0.00 | 0.00 |
| 3.5.99.10 | 0.10 | 0.01 | -0.06 | 0.00 | 0.06 | -0.11 | 0.05 | 0.25 | -0.04 |
| 1.4.1.16 | 0.09 | -0.03 | 0.01 | 0.06 | -0.03 | 0.03 | -0.01 | 0.01 | 0.01 |
| 4.1.1.49 | 0.09 | 0.02 | -0.03 | 0.06 | -0.01 | 0.00 | 0.03 | 0.02 | 0.04 |
| 1.14.99.48 | -0.09 | 0.28 | 0.12 | 0.23 | 0.17 | 0.17 | -0.52 | 0.00 | -0.48 |
| 4.2.1.46 | 0.09 | 0.02 | -0.03 | 0.06 | 0.01 | 0.03 | 0.11 | -0.17 | -0.12 |
| 3.6.1.23 | -0.09 | 0.15 | 0.38 | 0.02 | -0.42 | -0.41 | -0.24 | -0.32 | 0.25 |
| 1.6.5.11 | -0.09 | -0.08 | -0.01 | 0.05 | 0.05 | -0.07 | 0.06 | -0.01 | 0.01 |
| 3.6.3.14 | 0.09 | 0.09 | -0.07 | -0.11 | 0.07 | 0.02 | 0.01 | 0.03 | 0.16 |
| 3.4.21.92 | 0.09 | 0.20 | 0.11 | -0.12 | -0.16 | -0.32 | 0.17 | -0.22 | -0.17 |
| 3.2.2.27 | 0.09 | 0.05 | 0.00 | 0.00 | -0.03 | -0.05 | 0.03 | -0.05 | -0.06 |
| 2.1.3.9 | 0.09 | -0.04 | 0.01 | 0.05 | 0.01 | 0.00 | 0.00 | -0.02 | 0.01 |
| 1.2.1.12 | 0.09 | 0.11 | -0.06 | -0.07 | 0.02 | -0.15 | 0.09 | 0.03 | -0.14 |
| 3.6.4.13 | 0.09 | -0.07 | -0.01 | 0.05 | -0.02 | 0.00 | 0.01 | -0.01 | 0.03 |
| 2.7.7.65 | -0.08 | -0.05 | -0.03 | 0.04 | 0.01 | -0.05 | 0.05 | 0.01 | -0.01 |
| 3.5.99.6 | 0.08 | -0.01 | -0.01 | 0.09 | 0.06 | -0.02 | 0.04 | -0.08 | 0.01 |
| 1.1.1.22 | 0.08 | -0.01 | -0.01 | 0.06 | 0.00 | -0.03 | 0.01 | -0.01 | -0.04 |

**Supplementary Table S36 (continued).** Weights of top 52 original EC terms in the first nine principal components which cumulatively explains ~80% variance in the principal component analysis of the EC profiles of industrialized and non-industrialized gut microbiome. These ECs are mostly involved in microbial carbohydrate, amino acid, nucleotide, and energy metabolism pathways.

| EC | PC1 | PC2 | PC3 | PC4 | PC5 | PC6 | PC7 | PC8 | PC9 |
|---|---|---|---|---|---|---|---|---|---|
| 2.4.1.281 | 0.08 | -0.02 | 0.00 | 0.05 | 0.02 | 0.00 | 0.00 | -0.02 | 0.03 |
| 5.3.1.5 | 0.08 | -0.08 | 0.04 | -0.01 | -0.02 | 0.00 | -0.02 | 0.01 | -0.02 |
| 2.7.1.90 | 0.08 | -0.02 | 0.01 | 0.04 | 0.02 | -0.02 | 0.00 | -0.01 | 0.02 |
| 2.1.1.45 | 0.07 | -0.07 | 0.02 | -0.06 | -0.12 | 0.07 | 0.06 | -0.03 | -0.12 |
| 2.7.4.22 | 0.07 | 0.04 | -0.03 | -0.05 | -0.02 | -0.03 | 0.04 | 0.02 | -0.06 |
| 5.4.2.11 | 0.07 | -0.12 | 0.05 | -0.06 | 0.02 | 0.02 | 0.00 | -0.11 | -0.13 |
| 6.1.1.20 | 0.07 | -0.06 | 0.01 | -0.01 | -0.01 | 0.01 | -0.02 | -0.02 | 0.01 |
| 1.11.1.1 | 0.07 | 0.01 | 0.02 | -0.02 | 0.02 | -0.07 | -0.04 | 0.04 | -0.01 |
| 2.7.7.6 | 0.07 | 0.12 | -0.09 | -0.19 | -0.07 | 0.00 | 0.07 | -0.08 | -0.01 |
| 1.1.1.205 | 0.07 | 0.02 | -0.02 | 0.02 | 0.01 | 0.01 | 0.03 | -0.06 | 0.00 |
| 2.3.1.31 | 0.07 | -0.02 | 0.03 | -0.01 | 0.04 | 0.03 | 0.01 | -0.05 | -0.02 |
| 6.3.1.2 | 0.07 | -0.04 | 0.01 | 0.01 | 0.00 | 0.09 | -0.01 | -0.08 | 0.04 |
| 6.3.2.1 | 0.07 | 0.00 | -0.01 | 0.07 | -0.04 | 0.03 | 0.03 | 0.00 | 0.03 |
| 1.15.1.2 | 0.06 | 0.32 | -0.13 | 0.35 | 0.07 | -0.06 | 0.17 | -0.04 | 0.07 |
| 3.5.1.5 | -0.06 | 0.04 | 0.03 | -0.06 | -0.19 | 0.26 | 0.07 | -0.06 | -0.03 |

**Supplementary Table S37.** Top 100 significant ECs identified by Carnelian in the industrialized (Boston) vs non-industrialized (CEM: Cameroon, Ethiopia, and Madagascar) communities as differentially abundant. Significance thresholds used: BH corrected Wilcoxon ranksum test p-value < 0.05 and abs (log fold change) > 1. Using this threshold Carnelian identifies 454 differentially abundant ECs, whereas mi-faser, HUMAnN2, and Kraken2 identify 1009, 976, and 785 differentially abundant ECs respectively which covers > 80% of the ECs they identified in the population datasets; this indicates the presence of false positive hits among the reported ECs by other methods. Out of the 454 differentially abundant ECs identified by Carnelian, 284 were also reported significant by mi-faser, HUMAnN2, and Kraken2.

| EC | Boston Mean | CEM Mean | logFC | adjusted *p*-value | EC | Boston Mean | CEM Mean | logFC | adjusted *p*-value |
|---|---|---|---|---|---|---|---|---|---|
| 4.2.2.21 | 518.03 | 47.79 | -3.44 | 6.98E-37 | 2.4.1.319 | 1056.01 | 213.88 | -2.30 | 4.92E-37 |
| 5.3.1.5 | 2099.82 | 195.45 | -3.43 | 4.72E-37 | 2.1.1.298 | 45.69 | 225.46 | 2.30 | 1.03E-31 |
| 2.6.1.37 | 822.74 | 89.71 | -3.20 | 8.40E-37 | 4.1.3.38 | 25.49 | 125.74 | 2.30 | 1.72E-30 |
| 1.7.1.15 | 147.75 | 1325.02 | 3.16 | 1.41E-26 | 3.1.3.74 | 31.37 | 153.69 | 2.29 | 1.94E-27 |
| 2.4.1.320 | 1721.17 | 243.16 | -2.82 | 2.61E-37 | 1.14.11.17 | 31.05 | 151.99 | 2.29 | 2.59E-31 |
| 3.1.4.55 | 21.91 | 133.56 | 2.61 | 1.04E-29 | 3.4.21.83 | 18.80 | 91.91 | 2.29 | 2.43E-19 |
| 5.4.99.2 | 1622.17 | 273.81 | -2.57 | 7.87E-37 | 1.7.99.4 | 89.06 | 428.04 | 2.26 | 9.29E-19 |
| 4.2.2.24 | 338.41 | 57.16 | -2.57 | 6.98E-37 | 6.3.1.11 | 19.69 | 93.52 | 2.25 | 1.60E-30 |
| 3.1.4.52 | 173.24 | 1012.23 | 2.55 | 3.56E-34 | 1.1.98.6 | 39.86 | 189.23 | 2.25 | 2.68E-33 |
| 2.8.3.21 | 22.19 | 128.59 | 2.53 | 2.14E-32 | 3.2.2.3 | 35.60 | 169.01 | 2.25 | 5.53E-29 |
| 3.4.23.51 | 49.07 | 278.33 | 2.50 | 1.80E-24 | 1.3.1.91 | 37.66 | 178.03 | 2.24 | 1.15E-28 |
| 3.1.3.10 | 43.58 | 244.36 | 2.49 | 8.73E-31 | 3.1.4.14 | 36.06 | 168.78 | 2.23 | 1.41E-23 |
| 4.2.2.n1 | 112.94 | 631.86 | 2.48 | 1.81E-31 | 4.1.2.53 | 25.84 | 120.53 | 2.22 | 2.05E-33 |
| 4.2.2.8 | 404.41 | 73.68 | -2.46 | 4.60E-34 | 3.4.11.2 | 34.91 | 161.93 | 2.21 | 6.99E-33 |
| 1.3.8.13 | 24.70 | 134.75 | 2.45 | 2.22E-26 | 2.7.8.42 | 32.33 | 148.75 | 2.20 | 7.91E-34 |
| 3.2.1.196 | 23.67 | 126.04 | 2.41 | 3.78E-30 | 2.7.7.65 | 202.16 | 927.37 | 2.20 | 7.95E-33 |
| 1.13.11.29 | 29.60 | 157.60 | 2.41 | 1.20E-29 | 2.6.1.66 | 30.72 | 140.67 | 2.20 | 4.66E-25 |
| 3.1.11.5 | 55.33 | 290.59 | 2.39 | 7.09E-36 | 3.6.1.40 | 28.42 | 130.11 | 2.19 | 2.40E-28 |
| 2.1.1.197 | 23.42 | 121.63 | 2.38 | 3.46E-25 | 3.6.1.67 | 63.75 | 288.94 | 2.18 | 3.24E-29 |
| 1.17.4.1 | 141.07 | 723.18 | 2.36 | 5.26E-32 | 2.7.1.51 | 26.25 | 118.10 | 2.17 | 1.72E-26 |
| 2.1.1.61 | 16.76 | 85.93 | 2.36 | 4.96E-33 | 3.6.1.25 | 46.51 | 207.90 | 2.16 | 3.21E-31 |
| 2.4.1.12 | 30.44 | 154.96 | 2.35 | 1.56E-33 | 4.1.1.18 | 54.99 | 245.39 | 2.16 | 3.50E-19 |
| 4.1.1.98 | 26.97 | 136.66 | 2.34 | 6.68E-28 | 1.7.2.3 | 39.89 | 176.88 | 2.15 | 1.37E-26 |
| 1.6.1.2 | 104.46 | 523.96 | 2.33 | 2.43E-35 | 1.8.4.13 | 21.44 | 94.61 | 2.14 | 5.57E-24 |
| 1.14.99.46 | 18.18 | 90.09 | 2.31 | 4.66E-33 | 3.5.1.49 | 65.08 | 286.36 | 2.14 | 3.30E-23 |
| 3.2.2.28 | 62.60 | 271.14 | 2.11 | 1.13E-30 | 3.1.1.5 | 79.91 | 305.20 | 1.93 | 1.70E-26 |
| 1.17.5.3 | 21.61 | 93.58 | 2.11 | 6.13E-21 | 1.1.1.346 | 62.52 | 235.48 | 1.91 | 4.24E-30 |
| 2.7.7.42 | 25.12 | 108.24 | 2.11 | 1.12E-33 | 1.5.1.42 | 48.55 | 182.76 | 1.91 | 2.46E-21 |
| 6.4.1.3 | 4171.01 | 973.57 | -2.10 | 2.61E-37 | 3.4.16.4 | 210.09 | 789.76 | 1.91 | 1.96E-34 |
| 1.2.1.2 | 42.47 | 181.50 | 2.10 | 4.65E-33 | 2.3.1.15 | 54.52 | 204.64 | 1.91 | 1.94E-35 |
| 1.1.1.350 | 31.31 | 133.41 | 2.09 | 2.25E-32 | 1.2.7.3 | 1736.56 | 463.30 | -1.91 | 4.92E-37 |
| 1.3.5.1 | 119.56 | 507.47 | 2.09 | 1.63E-35 | 1.3.5.3 | 58.42 | 218.83 | 1.91 | 1.72E-22 |
| 3.6.3.12 | 951.46 | 224.17 | -2.09 | 2.81E-36 | 3.1.21.1 | 35.36 | 131.75 | 1.90 | 3.41E-26 |

**Supplementary Table S37 (continued).** Top 100 significant ECs identified by Carnelian in the industrialized (Boston) vs non-industrialized (CEM: Cameroon, Ethiopia, and Madagascar) communities as differentially abundant. Significance thresholds used: BH corrected Wilcoxon ranksum test p-value < 0.05 and abs (log fold change) > 1. Using this threshold Carnelian identifies 454 differentially abundant ECs, whereas mi-faser, HUMAnN2, and Kraken2 identify 1009, 976, and 785 differentially abundant ECs respectively which covers > 80% of the ECs they identified in the population datasets; this indicates the presence of false positive hits among the reported ECs by other methods. Out of the 454 differentially abundant ECs identified by Carnelian, 284 were also reported significant by mi-faser, HUMAnN2, and Kraken2.

| EC | Boston Mean | CEM Mean | logFC | adjusted *p*-value | EC | Boston Mean | CEM Mean | logFC | adjusted *p*-value |
|---|---|---|---|---|---|---|---|---|---|
| 3.2.1.169 | 565.15 | 134.48 | -2.07 | 1.52E-36 | 2.1.1.265 | 62.33 | 231.52 | 1.89 | 2.29E-28 |
| 2.7.7.61 | 52.66 | 220.29 | 2.06 | 8.20E-29 | 3.1.3.23 | 140.20 | 519.40 | 1.89 | 9.08E-25 |
| 2.6.1.19 | 27.37 | 114.47 | 2.06 | 3.81E-34 | 2.7.1.73 | 31.64 | 115.44 | 1.87 | 1.54E-28 |
| 1.14.11.33 | 31.68 | 131.78 | 2.06 | 8.65E-32 | 1.1.1.373 | 46.23 | 167.83 | 1.86 | 8.54E-33 |
| 1.16.1.9 | 33.18 | 137.78 | 2.05 | 1.61E-27 | 4.2.1.80 | 41.61 | 151.06 | 1.86 | 1.79E-25 |
| 6.3.2.45 | 45.09 | 186.88 | 2.05 | 4.05E-29 | 1.14.12.19 | 55.77 | 202.17 | 1.86 | 3.83E-22 |
| 4.2.1.3 | 25.45 | 102.64 | 2.01 | 1.13E-26 | 1.2.1.10 | 42.69 | 154.19 | 1.85 | 1.30E-24 |
| 2.8.1.2 | 31.16 | 124.83 | 2.00 | 1.85E-28 | 1.16.3.2 | 5201.73 | 1441.45 | -1.85 | 4.95E-36 |
| 3.5.4.1 | 88.68 | 354.08 | 2.00 | 5.47E-31 | 2.6.1.62 | 993.82 | 276.25 | -1.85 | 1.86E-36 |
| 2.3.1.29 | 1316.60 | 331.12 | -1.99 | 6.98E-37 | 1.13.11.39 | 250.61 | 69.69 | -1.85 | 2.74E-32 |
| 3.6.1.63 | 25.13 | 99.14 | 1.98 | 8.76E-32 | 5.3.1.22 | 30.11 | 108.12 | 1.84 | 4.42E-21 |
| 2.7.4.23 | 51.05 | 201.22 | 1.98 | 2.86E-21 | 1.8.5.3 | 38.01 | 135.60 | 1.84 | 1.11E-16 |
| 2.7.7.12 | 71.67 | 279.96 | 1.97 | 1.94E-35 | 2.3.1.193 | 24.71 | 87.79 | 1.83 | 2.74E-32 |
| 6.3.2.2 | 78.23 | 301.32 | 1.95 | 6.66E-34 | 2.7.7.19 | 34.00 | 120.30 | 1.82 | 3.00E-27 |
| 1.14.11.47 | 36.82 | 141.80 | 1.95 | 1.13E-30 | 3.2.1.170 | 23.36 | 82.40 | 1.82 | 3.14E-25 |
| 2.3.1.242 | 45.58 | 175.07 | 1.94 | 3.92E-28 | 3.5.3.26 | 42.59 | 149.04 | 1.81 | 5.15E-19 |
| 6.2.1.30 | 840.52 | 219.76 | -1.94 | 2.18E-36 | 6.3.5.3 | 200.16 | 698.17 | 1.80 | 3.85E-33 |

**Supplementary Table S47.** Pathways identified as significantly variable between the microbiomes of the industrialized (Boston) vs non-industrialized (CEM: Cameroon, Ethiopia, and Madagascar) communities using Carnelian-generated functional profiles. Significance thresholds used: BH-corrected Wilcoxon ranksum test p-value < 0.05 and abs (log fold change) > 1. When we take EC coverage of pathways into account, only six pathways remain significant. Here, SM = Biosynthesis of Secondary Metabolites; V = Metabolism of Co-factors and Vitamins; X = Xenobiotics Biodegradation and Metabolism.

| Category | Pathway ID | Pathway Name | logFC | Adjusted *p*-value | Coverage |
|---|---|---|---|---|---|
| SM | 00940 | Phenylpropanoid biosynthesis | -1.49 | 8.69E-38 | 0.16 |
| X | 00982 | Drug metabolism - cytochrome P450 | 1.33 | 5.31E-36 | 0.44 |
| X | 00980 | Metabolism of xenobiotics by cytochrome P450 | 1.02 | 1.73E-29 | 0.45 |
| X | 01501 | Beta-Lactam resistance | 1.01 | 3.31E-22 | 1.00 |
| V | 00785 | Lipoic acid metabolism | -1.02 | 3.79E-31 | 0.75 |
| SM | 00232 | Caffeine metabolism | 1.02 | 9.95E-31 | 0.31 |
| X | 00622 | Xylene degradation | 1.26 | 1.04E-29 | 0.18 |
| X | 00791 | Atrazine degradation | 1.11 | 1.35E-21 | 0.17 |
| X | 00642 | Ethylbenzene degradation | 1.19 | 6.69E-35 | 0.67 |
| X | 00626 | Naphthalene degradation | 1.10 | 8.06E-32 | 0.25 |
