# Supplementary material for: Carnelian uncovers hidden functional patterns across diverse study populations from whole metagenome sequencing reads
Source: Genome Biol. 2020 Feb 24;21:47. doi: 10.1186/s13059-020-1933-7 (PMC7038607; doi:10.1186/s13059-020-1933-7)
Supplement: Supplementary file 6 — Additional file 6 Results from Parkinson’s Disease Cohort. Contains Supplementary Tables S38–S46. [file 13059_2020_1933_MOESM6_ESM.pdf]

Carnelian uncovers hidden functional patterns across diverse study populations from whole metagenome sequencing reads

Sumaiya Nazeen[1], Yun William Yu[2], and Bonnie Berger[1,3*]

[1] Computer Science and Artificial Intelligence Laboratory (CSAIL), MIT, Cambridge, MA 02139, USA
[2] Department of Biomedical Informatics, HMS, Boston, MA 02115, USA
[3] Department of Mathematics, MIT, Cambridge, MA 02139, USA
* Corresponding Author: bab@mit.edu

Additional file 6 --- Results from Parkinson's Disease Cohort
Supplementary Tables S38 – S46

Supplementary Table S38. Significantly differentially abundant ECs between Parkinson's disease (PD) patients and controls identified by Carnelian in the PD-Bedarf data set. Significance thresholds used: BH corrected Wilcoxon rank-sum test p-value < 0.05 and abs (log fold change) > 0.58.

EC	pd_mean	ctrl_mean	fold-change	logFC	adj pval
1.17.4.1	321.80	521.70	0.62	-0.70	0.0424
1.4.1.16	939.29	1562.26	0.60	-0.73	0.0042
3.4.14.12	150.14	300.76	0.50	-1.00	0.0006
2.1.1.219	49.44	87.39	0.57	-0.82	0.0482
1.14.15.1	114.93	75.44	1.52	0.61	0.0424
2.4.1.11	78.74	50.79	1.55	0.63	0.0283
2.4.1.321	39.21	25.86	1.52	0.60	0.0264
2.4.1.320	475.67	813.99	0.58	-0.78	0.0065
1.7.2.2	228.03	345.73	0.66	-0.60	0.0283
3.11.1.1	317.62	503.03	0.63	-0.66	0.0264
5.3.1.17	938.09	1536.31	0.61	-0.71	0.0020
3.5.4.32	167.94	93.02	1.81	0.85	0.0011
2.7.14.1	326.91	205.91	1.59	0.67	0.0013
3.2.1.22	472.56	708.08	0.67	-0.58	0.0116
2.1.1.72	655.48	1106.87	0.59	-0.76	0.0065
2.1.1.74	2190.93	1305.18	1.68	0.75	0.0024
1.1.1.271	623.51	1044.16	0.60	-0.74	0.0039
2.7.1.209	65.80	38.97	1.69	0.76	0.0136
4.2.1.47	1241.63	1997.26	0.62	-0.69	0.0126
6.3.2.2	183.99	602.36	0.31	-1.71	0.0482
1.12.1.2	243.17	149.88	1.62	0.70	0.0008
1.16.3.2	1573.42	2525.69	0.62	-0.68	0.0077
2.1.3.9	923.70	1743.45	0.53	-0.92	0.0007
2.1.3.6	450.81	299.46	1.51	0.59	0.0246
3.5.3.18	83.90	52.22	1.61	0.68	0.0002
3.7.1.3	72.31	46.73	1.55	0.63	0.0158
3.8.1.2	91.06	57.79	1.58	0.66	0.0032
1.11.1.22	288.51	476.07	0.61	-0.72	0.0229
3.2.1.3	761.81	1591.14	0.48	-1.06	0.0055
1.7.1.13	1007.98	1549.66	0.65	-0.62	0.0005
3.4.13.9	447.01	749.37	0.60	-0.75	0.0452
3.1.1.72	243.41	364.27	0.67	-0.58	0.0397
3.1.1.73	170.77	336.39	0.51	-0.98	0.0264
3.1.4.16	360.65	197.80	1.82	0.87	0.0001
1.1.1.376	63.91	38.55	1.66	0.73	0.0264
3.2.1.86	1140.94	1887.71	0.60	-0.73	0.0000
3.2.1.80	177.76	268.21	0.66	-0.59	0.0116

Supplementary Table S38 (continued). Significantly differentially abundant ECs between Parkinson's disease (PD) patients and controls identified by Carnelian in the PD-Bedarf data set. Significance thresholds used: BH corrected Wilcoxon rank-sum test *p*-value < 0.05 and abs (log fold change) > 0.58.

EC	pd_mean	ctrl_mean	fold-change	logFC	adj pval
3.2.1.177	385.72	581.86	0.66	-0.59	0.0042
4.2.1.70	520.02	331.42	1.57	0.65	0.0046
2.1.1.181	81.27	132.08	0.62	-0.70	0.0424
4.1.1.32	627.01	338.17	1.85	0.89	0.0015
3.2.1.169	258.37	454.19	0.57	-0.81	0.0229
3.1.3.85	28.15	18.83	1.50	0.58	0.0482

Supplementary Table S39. Significantly differentially abundant ECs identified by mi-faser in the PD-Bedarf data set. Significance thresholds used: BH corrected Wilcoxon rank-sum test p-value < 0.05 and abs (log fold change) > 0.58.

EC	fold-change	logFC	adj pval	EC	fold-change	logFC	adj pval
1.1.5.3	0.58	-0.79	0.0309	3.5.5.1	0.66	-0.59	0.0375
3.1.4.46	0.59	-0.77	0.0351	2.4.1.279	1.75	0.81	0.0238
4.4.1.24	2.15	1.10	0.0033	1.5.1.43	0.67	-0.58	0.0144
4.4.1.21	0.50	-1.01	0.0112	4.2.2.26	3.28	1.71	0.0186
2.7.1.59	0.50	-1.00	0.0403	3.1.3.16	0.67	-0.58	0.0242
1.7.1.7	0.54	-0.88	0.0448	3.1.1.11	0.22	-2.19	0.0395
3.4.14.12	0.56	-0.84	0.0025	2.7.1.207	0.51	-0.98	0.0272
1.5.8.2	1.74	0.80	0.0231	5.4.99.27	0.55	-0.85	0.0302
2.1.1.217	0.47	-1.08	0.0379	2.8.2.22	0.59	-0.75	0.0343
3.6.1.54	0.44	-1.18	0.0317	4.2.1.42	0.49	-1.04	0.0068
1.3.1.91	0.57	-0.81	0.0087	4.2.1.40	0.53	-0.91	0.0058
6.1.2.1	1.59	0.67	0.0182	2.4.1.54	0.49	-1.02	0.0068
2.5.1.86	1.68	0.75	0.0010	2.2.1.10	2.52	1.33	0.0233
3.6.1.22	0.63	-0.67	0.0017	2.7.1.144	0.45	-1.16	0.0336
1.6.99.3	0.65	-0.62	0.0209	2.8.3.5	1.53	0.61	0.0035
3.11.1.1	0.56	-0.83	0.0137	5.5.1.27	3.21	1.68	0.0167
1.1.1.103	1.55	0.63	0.0448	1.12.1.2	1.97	0.98	0.0017
2.5.1.90	0.58	-0.77	0.0038	3.4.24.70	0.48	-1.05	0.0001
2.3.1.n4	0.63	-0.67	0.0376	3.4.24.78	1.59	0.67	0.0098
2.3.1.101	2.29	1.19	0.0151	1.8.98.1	1.74	0.80	0.0080
4.2.1.39	0.64	-0.64	0.0068	3.4.21.102	0.64	-0.65	0.0218
3.5.4.39	3.42	1.77	0.0124	2.8.1.12	0.59	-0.76	0.0494
2.7.1.130	0.50	-0.99	0.0041	2.7.1.193	0.64	-0.64	0.0309
2.7.14.1	1.77	0.82	0.0098	3.5.3.23	0.45	-1.17	0.0307
3.4.15.5	0.52	-0.95	0.0001	2.4.1.282	3.60	1.85	0.0006
5.3.99.11	0.43	-1.23	0.0048	2.1.1.35	0.55	-0.86	0.0189
4.1.1.75	0.59	-0.77	0.0330	2.1.1.193	0.67	-0.58	0.0151
2.1.1.74	1.55	0.63	0.0030	5.1.3.3	0.52	-0.95	0.0412
2.1.1.242	0.46	-1.11	0.0273	1.12.2.1	2.25	1.17	0.0311
2.8.1.6	0.60	-0.73	0.0159	1.1.1.38	0.63	-0.67	0.0467
2.7.8.8	0.50	-0.99	0.0464	3.4.17.11	0.60	-0.74	0.0231
2.3.1.263	1.60	0.68	0.0388	1.2.1.92	0.46	-1.12	0.0483
3.1.3.27	0.52	-0.94	0.0408	5.4.2.8	0.63	-0.67	0.0351
3.1.3.25	0.61	-0.71	0.0199	5.1.3.20	0.63	-0.67	0.0032
1.3.4.1	2.29	1.20	0.0491	2.3.1.12	0.54	-0.89	0.0249
1.1.1.310	2.38	1.25	0.0209	3.3.1.1	1.50	0.58	0.0076

Supplementary Table S39 (continued). Significantly differentially abundant ECs identified by mi-faser in the PD-Bedarf data set. Significance thresholds used: BH corrected Wilcoxon rank-sum test p-value < 0.05 and abs (log fold change) > 0.58.

EC	fold-change	logFC	adj p	EC	fold-change	logFC	adj p
2.6.1.14	0.66	-0.61	0.0108	1.3.1.39	2.31	1.21	0.0058
1.1.1.308	1.97	0.97	0.0039	1.1.1.350	0.52	-0.95	0.0121
1.1.1.304	0.51	-0.98	0.0486	1.1.1.24	4.91	2.30	0.0416
2.7.1.219	0.21	-2.22	0.0466	3.5.4.3	0.64	-0.64	0.0024
3.2.1.122	0.55	-0.87	0.0006	3.1.4.16	1.91	0.93	0.0004
3.3.2.1	0.48	-1.07	0.0206	3.5.2.17	0.62	-0.70	0.0318
3.4.25.2	1.53	0.61	0.0414	3.2.1.85	0.51	-0.96	0.0131
4.2.1.82	0.60	-0.73	0.0284	3.6.1.67	0.55	-0.86	0.0496
1.11.1.22	0.58	-0.78	0.0044	1.1.1.383	2.06	1.04	0.0030
2.1.1.265	0.51	-0.98	0.0373	1.1.1.385	0.41	-1.28	0.0315
3.5.4.40	0.39	-1.37	0.0316	3.5.1.108	0.57	-0.81	0.0137
3.5.4.41	1.50	0.58	0.0249	5.3.2.8	0.47	-1.09	0.0217
3.1.3.45	0.47	-1.10	0.0336	3.1.13.5	0.59	-0.77	0.0449
1.1.98.6	0.58	-0.78	0.0431	2.7.1.5	0.63	-0.67	0.0034
1.1.1.298	0.49	-1.01	0.0001	1.9.3.1	0.23	-2.14	0.0228
2.5.1.30	1.66	0.73	0.0174	3.2.1.99	0.46	-1.13	0.0358
3.1.26.8	0.45	-1.17	0.0096	4.1.99.19	0.61	-0.72	0.0489
1.4.1.24	2.69	1.43	0.0249	4.1.3.4	2.60	1.38	0.0219
1.4.1.3	1.63	0.71	0.0414	4.1.3.3	0.51	-0.99	0.0280
3.4.11.7	0.50	-0.99	0.0314	3.4.21.72	0.27	-1.89	0.0196
1.1.1.69	0.64	-0.65	0.0012	1.14.13.2	0.12	-3.11	0.0355
5.4.3.5	1.53	0.62	0.0063	4.3.1.24	0.33	-1.59	0.0327
4.1.1.32	1.94	0.95	0.0014	3.4.11.9	0.56	-0.84	0.0343
3.2.1.165	0.08	-3.64	0.0096				

Supplementary Table S40. Significantly differentially abundant ECs identified by HUMAnN2 in the PD-Bedarf data set. Significance thresholds used: BH corrected Wilcoxon rank-sum test p-value < 0.05 and abs (log fold change) > 0.58.

EC	fold-change	logFC	adj p	EC	fold-change	logFC	adj p
1.1.5.2	0.51	-0.99	0.0327	2.7.8.14	50.29	5.65	0.0271
2.7.7.19	0.55	-0.85	0.0201	5.4.99.2	1.61	0.69	0.0174
4.4.1.24	9.95	3.31	0.0216	1.3.3.11	0.20	-2.32	0.0399
4.4.1.21	0.63	-0.67	0.0441	5.4.99.27	0.50	-1.00	0.0395
2.7.1.59	0.55	-0.86	0.0332	5.4.99.24	0.61	-0.71	0.0415
3.5.1.104	1.82	0.86	0.0323	5.3.1.28	0.45	-1.14	0.0100
1.1.1.58	0.65	-0.62	0.0166	3.6.3.4	1.79	0.84	0.0173
1.2.7.3	2.75	1.46	0.0197	3.4.21.116	3.15	1.66	0.0021
3.6.1.55	0.54	-0.90	0.0178	2.7.1.148	0.57	-0.82	0.0468
1.3.1.91	0.62	-0.70	0.0366	6.4.1.1	2.87	1.52	0.0005
2.5.1.86	6.01	2.59	0.0188	3.6.5.n1	1.58	0.66	0.0030
4.1.1.17	0.64	-0.64	0.0226	3.4.21.92	1.59	0.66	0.0047
2.4.2.3	0.54	-0.89	0.0382	6.3.2.2	0.50	-1.00	0.0445
2.4.2.2	1.62	0.69	0.0119	4.2.1.2	1.55	0.63	0.0358
3.5.4.27	6.91	2.79	0.0474	6.3.2.8	0.59	-0.75	0.0244
2.3.1.241	0.55	-0.85	0.0361	6.3.4.3	1.72	0.78	0.0021
2.3.1.247	2.29	1.20	0.0076	2.7.10.2	2.21	1.14	0.0146
2.7.7.39	2.41	1.27	0.0158	2.3.1.35	49.42	5.63	0.0253
1.2.1.11	0.55	-0.87	0.0373	1.10.3.14	0.64	-0.63	0.0337
2.5.1.90	0.50	-1.01	0.0463	2.7.1.193	0.46	-1.13	0.0345
2.3.1.n4	0.30	-1.75	0.0029	1.16.3.1	0.46	-1.12	0.0260
5.3.3.14	0.39	-1.37	0.0046	3.5.3.7	0.22	-2.17	0.0427
3.5.4.32	6.52	2.70	0.0361	2.7.1.25	0.55	-0.87	0.0090
2.1.2.9	0.58	-0.78	0.0384	2.1.1.193	0.48	-1.07	0.0284
1.2.7.1	1.77	0.82	0.0030	2.1.1.198	0.46	-1.12	0.0450
2.3.2.6	0.43	-1.23	0.0332	5.1.3.9	0.63	-0.66	0.0229
6.3.4.20	0.66	-0.59	0.0042	2.1.1.171	0.44	-1.17	0.0158
2.7.8.8	0.49	-1.04	0.0270	2.1.1.173	5.26	2.40	0.0316
2.3.1.263	3.98	1.99	0.0056	1.1.1.346	0.43	-1.22	0.0325
2.7.9.1	1.75	0.81	0.0024	1.1.1.38	0.57	-0.81	0.0331
3.1.3.27	0.56	-0.85	0.0297	2.3.3.13	0.61	-0.70	0.0490
3.1.3.25	0.60	-0.73	0.0345	2.1.1.189	0.50	-1.01	0.0342
5.1.1.13	3.56	1.83	0.0007	6.4.1.3	2.03	1.02	0.0125
2.7.6.1	0.63	-0.66	0.0491	2.3.1.180	0.65	-0.62	0.0324
2.6.1.11	1.79	0.84	0.0442	3.3.1.1	2.07	1.05	0.0045
4.2.2.24	4.13	2.05	0.0070	2.6.1.48	0.49	-1.02	0.0086
3.6.3.29	2.56	1.36	0.0311	4.1.2.14	0.51	-0.98	0.0204

Supplementary Table S40 (continued). Significantly differentially abundant ECs identified by HUMAnN2 in the PD-Bedarf data set. Significance thresholds used: BH corrected Wilcoxon rank-sum test p-value < 0.05 and abs (log fold change) > 0.58.

EC	fold-change	logFC	adj pval	EC	fold-change	logFC	adj pval
2.3.1.54	1.63	0.71	0.0182	2.7.7.56	0.67	-0.59	0.0403
4.4.1.8	0.57	-0.81	0.0366	4.2.1.82	0.23	-2.13	0.0167
4.4.1.5	0.44	-1.18	0.0485	1.1.1.385	6.61	2.72	0.0483
3.1.3.45	0.38	-1.38	0.0049	2.5.1.55	0.67	-0.58	0.0446
2.7.1.17	0.51	-0.98	0.0397	4.2.1.1	0.51	-0.97	0.0397
1.3.1.108	1.71	0.77	0.0372	2.7.7.75	0.51	-0.96	0.0191
5.1.1.7	0.64	-0.65	0.0188	3.6.3.42	5.23	2.39	0.0016
6.5.1.2	0.58	-0.79	0.0167	1.14.11.47	0.52	-0.93	0.0318
5.4.99.19	0.46	-1.13	0.0070	1.5.1.3	0.52	-0.94	0.0403
1.1.1.283	4.23	2.08	0.0446	1.1.1.60	0.59	-0.75	0.0190
3.5.2.17	0.53	-0.91	0.0236	2.7.7.6	1.53	0.62	0.0027
3.2.1.85	0.45	-1.14	0.0452	2.1.1.181	0.63	-0.67	0.0497
6.3.5.7	1.98	0.98	0.0032	5.4.3.5	3.76	1.91	0.0022
1.2.4.2	0.58	-0.79	0.0248	5.4.3.4	1.96	0.97	0.0023
4.1.2.25	0.35	-1.50	0.0291	5.4.3.3	1.89	0.92	0.0028
4.1.2.21	0.63	-0.66	0.0450	5.4.3.2	2.00	1.00	0.0158
4.3.1.3	1.70	0.76	0.0425	4.1.1.32	4.26	2.09	0.0022
1.1.1.383	1.66	0.73	0.0397	4.1.1.37	0.63	-0.67	0.0483
2.3.1.174	2.40	1.26	0.0086	6.3.4.18	0.64	-0.65	0.0417

Supplementary Table S41. Significantly differentially abundant ECs identified by Kraken2 in the PD-Bedarf data set. Significance thresholds used: BH corrected Wilcoxon rank-sum test p-value < 0.05 and abs (log fold change) > 0.58.

EC	fold-change	logFC	adj pval	EC	fold-change	logFC	adj pval
4.4.1.21	0.64	-0.65	0.0152	4.1.1.87	2.87	1.52	0.0068
2.5.1.72	0.64	-0.65	0.0052	4.1.1.81	1.56	0.64	0.0208
3.5.1.104	1.78	0.83	0.0448	5.4.99.17	2.53	1.34	0.0295
1.1.1.53	3.65	1.87	0.0007	6.3.2.5	0.65	-0.62	0.0249
1.2.7.8	1.55	0.63	0.0174	2.1.1.298	0.67	-0.59	0.0296
3.4.14.12	0.52	-0.95	0.0018	1.8.98.1	1.57	0.65	0.0467
1.13.11.75	0.33	-1.59	0.0296	3.1.3.78	4.54	2.18	0.0062
1.1.99.6	0.47	-1.08	0.0111	3.4.21.105	0.56	-0.84	0.0468
1.1.1.302	0.52	-0.95	0.0018	3.1.1.31	0.55	-0.87	0.0268
2.5.1.86	1.62	0.69	0.0006	3.5.3.23	0.52	-0.95	0.0194
4.2.2.6	0.11	-3.24	0.0148	2.1.1.289	0.20	-2.33	0.0007
2.4.1.11	2.73	1.45	0.0295	6.6.1.1	1.63	0.71	0.0043
2.4.1.12	0.52	-0.94	0.0096	2.7.4.29	0.56	-0.84	0.0463
6.3.2.44	1.59	0.67	0.0472	2.1.1.196	1.94	0.96	0.0068
4.1.1.104	0.51	-0.98	0.0115	2.3.1.228	2.19	1.13	0.0210
3.11.1.1	0.53	-0.92	0.0199	1.14.14.5	0.53	-0.91	0.0231
1.1.1.107	1.65	0.72	0.0310	4.2.3.170	9.48	3.25	0.0187
2.4.1.250	1.70	0.76	0.0079	1.1.1.35	1.58	0.66	0.0025
1.14.15.12	0.35	-1.51	0.0164	1.1.2.8	2.11	1.07	0.0082
2.7.14.1	1.77	0.83	0.0103	1.2.99.8	2.53	1.34	0.0145
2.1.1.74	1.68	0.75	0.0007	2.4.99.21	2.50	1.32	0.0108
5.5.1.16	12.33	3.62	0.0022	2.6.1.48	0.65	-0.63	0.0209
3.1.3.25	0.60	-0.73	0.0065	4.1.2.14	0.62	-0.68	0.0361
1.3.4.1	2.21	1.15	0.0485	2.7.7.1	4.12	2.04	0.0039
2.7.4.2	0.56	-0.83	0.0258	1.1.1.350	0.47	-1.10	0.0133
2.3.1.129	0.49	-1.03	0.0454	4.2.1.109	15.68	3.97	0.0253
2.4.1.279	1.91	0.93	0.0065	3.5.4.3	0.67	-0.58	0.0103
2.3.1.94	2.28	1.19	0.0303	2.7.1.219	0.65	-0.63	0.0454
3.6.4.9	2.77	1.47	0.0050	3.2.1.122	0.56	-0.83	0.0028
4.2.2.26	2.22	1.15	0.0039	3.8.1.7	0.33	-1.59	0.0436
3.2.2.8	0.53	-0.92	0.0241	3.4.23.42	1.98	0.99	0.0304
1.12.98.4	2.01	1.01	0.0079	4.2.3.154	0.51	-0.98	0.0459
1.12.98.2	2.01	1.01	0.0385	1.1.1.298	0.54	-0.88	0.0317
4.2.1.40	0.52	-0.93	0.0093	1.7.1.15	0.57	-0.82	0.0440
5.3.1.29	2.12	1.09	0.0355	1.1.1.14	1.56	0.64	0.0293
2.4.2.36	3.70	1.89	0.0413	5.4.99.19	0.61	-0.71	0.0358
2.4.2.31	0.50	-1.00	0.0211	1.1.1.286	1.86	0.89	0.0431

Supplementary Table S41 (continued). Significantly differentially abundant ECs identified by Kraken2 in the PD-Bedarf data set. Significance thresholds used: BH corrected Wilcoxon rank-sum test p-value < 0.05 and abs (log fold change) > 0.58.

EC	fold-change	logFC	adj pval
3.1.4.16	1.88	0.91	0.0024
3.2.1.85	0.46	-1.11	0.0058
2.1.1.315	1.75	0.80	0.0206
3.5.1.108	0.61	-0.72	0.0454
3.4.21.72	2.08	1.06	0.0012
3.5.1.25	0.64	-0.65	0.0058
1.3.5.1	0.66	-0.60	0.0166
2.5.1.113	0.67	-0.59	0.0166
1.14.13.2	0.19	-2.39	0.0216
4.3.1.23	2.84	1.51	0.0204
3.4.11.7	0.47	-1.07	0.0431
5.4.3.5	1.69	0.76	0.0013
4.1.1.32	1.91	0.94	0.0034

Supplementary Table S42. Pathways identified as significantly variable between PD patients and healthy controls in the PD-Bedarf data set using Carnelian-generated functional profiles. Significance thresholds used: BH corrected Wilcoxon rank-sum test p-value < 0.05 and abs (log fold change) > 0.11. Here, C = Carbohydrate Metabolism; L = Lipid Metabolism; E = Energy Metabolism; N = Nucleotide Metabolism; AA = Amino Acid Metabolism (includes metabolism of other amino acids as well); SM = Biosynthesis of Secondary Metabolites; G = Glycan Biosynthesis and Metabolism; V = Metabolism of Co-factors and Vitamins; X = Xenobiotics Biodegradation and Metabolism; GI = Genetic Information Processing; T = Metabolism of Terpenoids and Polyketides.

Category	ID	Name	Fold Change	logFC	adjusted p-value
C	00040	Pentose and glucuronate interconversions	0.83	-0.31	0.00089
C	00051	Fructose and mannose metabolism	0.90	-0.22	0.00386
C	00052	Galactose metabolism	0.92	-0.18	0.00134
C	00053	Ascorbate and aldarate metabolism	0.85	-0.28	0.01709
AA	00250	Alanine, aspartate and glutamate metabolism	0.92	-0.18	0.02132
X	00362	Benzoate degradation	1.11	0.15	0.00148
AA	00400	Phenylalanine, tyrosine and tryptophan biosynthesis	0.88	-0.19	0.01899
AA	00473	D-Alanine metabolism	1.19	0.25	0.00293
AA	00480	Glutathione metabolism	0.71	-0.49	0.00164
C	00500	Starch and sucrose metabolism	0.91	-0.19	0.00200
SM	00521	Streptomycin biosynthesis	0.87	-0.24	0.03968
T	00523	Polyketide sugar unit biosynthesis	0.84	-0.32	0.04518
G	00531	Glycosaminoglycan degradation	0.82	-0.32	0.03715
G	00550	Peptidoglycan biosynthesis	1.15	0.20	0.00220
G	00571	Lipoarabinomannan (LAM) biosynthesis	1.26	0.34	0.00601
L	00600	Sphingolipid metabolism	0.79	-0.35	0.01074
L	00603	Glycosphingolipid biosynthesis - globo and isoglobo series	0.74	-0.43	0.03248
L	00604	Glycosphingolipid biosynthesis - ganglio series	0.80	-0.37	0.03715
V	00790	Folate biosynthesis	0.91	-0.13	0.03034
V	00860	Porphyrin and chlorophyll metabolism	1.08	0.12	0.04816
T	00908	Zeatin biosynthesis	1.19	0.26	0.00991
X	00983	Drug metabolism - other enzymes	0.93	-0.19	0.00655
SM	00999	Biosynthesis of secondary metabolites - unclassified	1.10	0.15	0.02640
T	01055	Biosynthesis of vancomycin group antibiotics	0.82	-0.36	0.04236

Supplementary Table S43. Pathways identified as significantly variable between PD patients and healthy controls in the PD-Bedarf data set using mi-faser-generated functional profiles. Significance thresholds used: BH corrected Wilcoxon rank-sum test p-value < 0.05 and abs (log fold change) > 0.11. Here, C = Carbohydrate Metabolism; L = Lipid Metabolism; E = Energy Metabolism; N = Nucleotide Metabolism; AA = Amino Acid Metabolism (includes metabolism of other amino acids as well); SM = Biosynthesis of Secondary Metabolites; G = Glycan Biosynthesis and Metabolism; V = Metabolism of Co-factors and Vitamins; X = Xenobiotics Biodegradation and Metabolism; GI = Genetic Information Processing; T = Metabolism of Terpenoids and Polyketides.

Category	ID	Name	Fold Change	logFC	adj pval
C	00040	Pentose and glucuronate interconversions	0.81	-0.31	0.0022
C	00051	Fructose and mannose metabolism	0.86	-0.21	0.0099
C	00052	Galactose metabolism	0.89	-0.18	0.0018
C	00053	Ascorbate and aldarate metabolism	0.81	-0.31	0.0099
L	00062	Fatty acid elongation	0.73	-0.45	0.0482
T	00130	Ubiquinone and other terpenoid-quinone biosynthesis	0.66	-0.60	0.0184
N	00230	Purine metabolism	1.08	0.12	0.0071
N	00240	Pyrimidine metabolism	1.14	0.19	0.0010
SM	00405	Phenazine biosynthesis	0.84	-0.25	0.0283
AA	00440	Phosphonate and phosphinate metabolism	0.58	-0.79	0.0229
AA	00450	Selenocompound metabolism	0.92	-0.13	0.0184
AA	00472	D-Arginine and D-ornithine metabolism	1.61	0.69	0.0116
AA	00480	Glutathione metabolism	0.74	-0.43	0.0158
C	00500	Starch and sucrose metabolism	0.89	-0.16	0.0264
G	00511	Other glycan degradation	0.66	-0.60	0.0007
G	00531	Glycosaminoglycan degradation	0.66	-0.59	0.0005
C	00562	Inositol phosphate metabolism	1.20	0.26	0.0065
L	00600	Sphingolipid metabolism	0.69	-0.53	0.0016
L	00604	Glycosphingolipid biosynthesis - ganglio series	0.66	-0.60	0.0009
X	00633	Nitrotoluene degradation	1.13	0.18	0.0325
V	00670	One carbon pool by folate	1.09	0.12	0.0424
V	00780	Biotin metabolism	0.87	-0.20	0.0042
V	00790	Folate biosynthesis	0.81	-0.31	0.0013
T	00903	Limonene and pinene degradation	0.72	-0.48	0.0371
T	00908	Zeatin biosynthesis	1.35	0.44	0.0020
X	00930	Caprolactam degradation	0.69	-0.54	0.0424
GI	00970	Aminoacyl-tRNA biosynthesis	1.11	0.15	0.0055
X	00983	Drug metabolism - other enzymes	0.92	-0.12	0.0012
X	00984	Steroid degradation	0.67	-0.57	0.0303
SM	00999	Biosynthesis of secondary metabolites - unclassified	1.26	0.33	0.0032

Supplementary Table S44. Pathways identified as significantly variable between PD patients and healthy controls in the PD-Bedarf data set functional profiles generated by HUMAnN2. Significance thresholds used: BH corrected Wilcoxon rank-sum test p-value < 0.05 and abs (log fold change) > 0.11. Here, C = Carbohydrate Metabolism; L = Lipid Metabolism; E = Energy Metabolism; N = Nucleotide Metabolism; AA = Amino Acid Metabolism (includes metabolism of other amino acids as well); SM = Biosynthesis of Secondary Metabolites; G = Glycan Biosynthesis and Metabolism; V = Metabolism of Co-factors and Vitamins; X = Xenobiotics Biodegradation and Metabolism; GI = Genetic Information Processing; T = Metabolism of Terpenoids and Polyketides.

Category	ID	Name	Fold Change	logFC	Adjusted p-value
C	00020	Citrate cycle (TCA cycle)	1.21	0.28	0.005511
C	00030	Pentose phosphate pathway	0.82	-0.29	0.015847
C	00040	Pentose and glucuronate interconversions	0.76	-0.39	0.030336
C	00052	Galactose metabolism	0.88	-0.19	0.008417
L	00072	Synthesis and degradation of ketone bodies	1.33	0.41	0.026405
N	00230	Purine metabolism	1.33	0.41	0.002425
N	00240	Pyrimidine metabolism	1.43	0.52	0.007124
SM	00261	Monobactam biosynthesis	0.81	-0.31	0.032480
AA	00280	Valine, leucine and isoleucine degradation	1.37	0.45	0.012579
AA	00310	Lysine degradation	1.71	0.77	0.001641
AA	00410	beta-Alanine metabolism	0.62	-0.69	0.030336
AA	00472	D-Arginine and D-ornithine metabolism	4.55	2.18	0.001629
C	00500	Starch and sucrose metabolism	0.72	-0.47	0.028313
C	00620	Pyruvate metabolism	1.27	0.34	0.000714
X	00633	Nitrotoluene degradation	1.67	0.74	0.000325
C	00640	Propanoate metabolism	1.33	0.42	0.000032
C	00650	Butanoate metabolism	1.21	0.28	0.022911
V	00670	One carbon pool by folate	1.23	0.30	0.009910
E	00680	Methane metabolism	1.29	0.37	0.003526
E	00710	Carbon fixation in photosynthetic organisms	1.29	0.37	0.019818
E	00720	Carbon fixation pathways in prokaryotes	1.52	0.60	0.000021
C	00770	Pantothenate and CoA biosynthesis	0.71	-0.50	0.015847

Supplementary Table S45. Pathways identified as significantly variable between PD patients and healthy controls in the PD-Bedarf data set functional profiles generated by Kraken2. Significance thresholds used: BH corrected Wilcoxon rank-sum test p-value < 0.05 and abs (log fold change) > 0.11. Here, C = Carbohydrate Metabolism; L = Lipid Metabolism; E = Energy Metabolism; N = Nucleotide Metabolism; AA = Amino Acid Metabolism (includes metabolism of other amino acids as well); SM = Biosynthesis of Secondary Metabolites; G = Glycan Biosynthesis and Metabolism; V = Metabolism of Co-factors and Vitamins; X = Xenobiotics Biodegradation and Metabolism; GI = Genetic Information Processing; T = Metabolism of Terpenoids and Polyketides.

Category	ID	Name	Fold Change	logFC	Adjusted p-value
C	00040	Pentose and glucuronate interconversions	0.79	-0.35	0.0016
C	00051	Fructose and mannose metabolism	0.89	-0.17	0.0264
C	00052	Galactose metabolism	0.85	-0.23	0.0002
C	00053	Ascorbate and aldarate metabolism	0.80	-0.32	0.0084
L	00120	Primary bile acid biosynthesis	1.76	0.81	0.0013
L	00140	Steroid hormone biosynthesis	4.82	2.27	0.0014
N	00230	Purine metabolism	1.10	0.13	0.0126
N	00240	Pyrimidine metabolism	1.17	0.22	0.0006
AA	00250	Alanine, aspartate and glutamate metabolism	0.91	-0.14	0.0126
AA	00330	Arginine and proline metabolism	0.90	-0.15	0.0482
AA	00340	Histidine metabolism	0.88	-0.18	0.0171
AA	00400	Phenylalanine, tyrosine and tryptophan biosynthesis	0.90	-0.15	0.0325
AA	00450	Selenocompound metabolism	0.85	-0.23	0.0046
AA	00472	D-Arginine and D-ornithine metabolism	1.51	0.59	0.0091
AA	00480	Glutathione metabolism	0.75	-0.41	0.0147
C	00500	Starch and sucrose metabolism	0.91	-0.13	0.0371
G	00511	Other glycan degradation	0.75	-0.42	0.0099
C	00520	Amino sugar and nucleotide sugar metabolism	0.92	-0.13	0.0452
G	00531	Glycosaminoglycan degradation	0.75	-0.42	0.0055
G	00540	Lipopolysaccharide biosynthesis	0.72	-0.47	0.0397
G	00550	Peptidoglycan biosynthesis	1.13	0.18	0.0424
L	00604	Glycosphingolipid biosynthesis - ganglio series	0.75	-0.42	0.0099
X	00623	Toluene degradation	1.82	0.87	0.0006
V	00740	Riboflavin metabolism	0.71	-0.48	0.0032
V	00790	Folate biosynthesis	0.78	-0.37	0.0029
T	00908	Zeatin biosynthesis	1.30	0.38	0.0229
E	00910	Nitrogen metabolism	0.90	-0.15	0.0482
GI	00970	Aminoacyl-tRNA biosynthesis	1.15	0.20	0.0032
SM	00999	Biosynthesis of secondary metabolites - unclassified	1.25	0.32	0.0136

Supplementary Table S46. Pathways identified as significantly variable between PD patients and healthy controls in the PD-Bedarf data set functional profiles generated by out-of-the-box HUMAnN2 using ChocoPhlAn, Uniref, and MetaCyc databases. Significance thresholds used: BH corrected Wilcoxon rank-sum test p-value < 0.05, abs (log fold change) > 0.11 and coverage > 0.10. Here, L = Lipid Metabolism; N = Nucleotide Metabolism; AA = Amino Acid Metabolism (includes metabolism of other amino acids as well); V = Metabolism of Co-factors and Vitamins; T = Metabolism of Terpenoids and Polyketides.

Category	MetaCyc ID	Name	FC	logFC	adj p-value	coverage
V	1CMET2-PWY	N^{10}-formyl-tetrahydrofolate biosynthesis	0.66	-0.60	0.03	0.55
AA	ASPASN-PWY	Superpathway of L-aspartate and L-asparagine biosynthesis	0.59	-0.76	0.01	0.17
T	NONMEVIPP-PWY	methylerythritol phosphate pathway I	0.66	-0.59	0.04	0.95
V	PANTO-PWY	phosphopantothenate biosynthesis I	0.66	-0.59	0.03	0.86
AA	PWY-2942	L-lysine biosynthesis III	0.66	-0.59	0.03	0.95
V	PWY-3841	folate transformations II	0.68	-0.55	0.04	0.64
AA	PWY-5097	L-lysine biosynthesis VI	0.68	-0.55	0.02	0.94
L	PWY-5667	CDP-diacylglycerol biosynthesis I	0.64	-0.65	0.01	0.88
N	PWY-5686	UMP biosynthesis I	0.69	-0.53	0.04	1.00
N	PWY-5695	inosine 5'-phosphate degradation	0.60	-0.74	0.02	0.77
L	PWY-5973	*cis*-vaccenate biosynthesis	0.62	-0.70	0.01	0.70
N	PWY-6126	superpathway of adenosine nucleotides *de novo* biosynthesis II	0.62	-0.69	0.04	0.18
AA	PWY-6151	*S*-adenosyl-L-methionine cycle I	0.57	-0.82	0.01	0.95
N	PWY-6609	adenine and adenosine salvage III	0.71	-0.49	0.02	0.15
N	PWY-6700	queuosine biosynthesis	0.61	-0.72	0.03	0.80
V	PWY-6897	thiamine salvage II	0.65	-0.63	0.04	0.19
N	PWY-7219	adenosine ribonucleotides *de novo* biosynthesis	0.67	-0.58	0.03	1.00
N	PWY-7221	guanosine ribonucleotides *de novo* biosynthesis	0.67	-0.58	0.04	1.00
N	PWY-7229	superpathway of adenosine nucleotides *de novo* biosynthesis I	0.63	-0.66	0.03	0.19
N	PWY0-1296	purine ribonucleosides degradation	0.62	-0.70	0.04	0.27
L	PWY0-1319	CDP-diacylglycerol biosynthesis II	0.64	-0.65	0.01	0.88
L	PWY4FS-7	phosphatidylglycerol biosynthesis I (plastidic)	0.50	-0.99	0.05	0.12
L	PWY4FS-8	phosphatidylglycerol biosynthesis II (non-plastidic)	0.50	-0.99	0.05	0.12
