# Supplementary material for: Carnelian uncovers hidden functional patterns across diverse study populations from whole metagenome sequencing reads
Source: Genome Biol. 2020 Feb 24;21:47. doi: 10.1186/s13059-020-1933-7 (PMC7038607; doi:10.1186/s13059-020-1933-7)
Supplement: Supplementary file 7 — Additional file 7 Metadata of Study Cohorts. Contains Supplementary Tables S48–S56. [file 13059_2020_1933_MOESM7_ESM.pdf]

Carnelian uncovers hidden functional patterns across diverse study populations from whole metagenome sequencing reads

Sumaiya Nazeen[1], Yun William Yu[2], and Bonnie Berger[1,3*]

[1] Computer Science and Artificial Intelligence Laboratory (CSAIL), MIT, Cambridge, MA 02139, USA
[2] Department of Biomedical Informatics, HMS, Boston, MA 02115, USA
[3] Department of Mathematics, MIT, Cambridge, MA 02139, USA
* Corresponding Author: bab@mit.edu

Additional file 7 --- Metadata of Study Cohorts
Supplementary Tables S48 – S56

Supplementary Table S48. Metadata of 241 samples from Qin et al. 2012 study which survived quality control steps (T2D-Qin data set).

Accession No.	Group	Paired-Len (AA)	Gender	Accession No.	Group	Paired-Len (AA)	Gender
SRR1778451	T2D	101	male	SRR341686	T2D	101	male
SRR1778452	T2D	101	female	SRR341687	T2D	101	male
SRR1778453	T2D	101	female	SRR341688	T2D	101	male
SRR1778454	T2D	101	male	SRR341689	T2D	101	male
SRR1778455	T2D	101	male	SRR341690	Normal	101	female
SRR1778456	T2D	101	male	SRR341691	Normal	101	female
SRR341654	T2D	101	female	SRR341692	Normal	101	female
SRR341655	T2D	101	female	SRR341693	Normal	101	female
SRR341656	T2D	101	female	SRR341694	Normal	101	male
SRR341657	T2D	101	female	SRR341695	Normal	101	male
SRR341658	T2D	101	male	SRR341696	Normal	101	male
SRR341659	T2D	101	male	SRR341697	Normal	101	male
SRR341660	T2D	101	male	SRR341698	Normal	101	male
SRR341661	T2D	101	male	SRR341699	Normal	101	male
SRR341662	T2D	101	male	SRR341700	Normal	101	male
SRR341663	T2D	101	male	SRR341701	Normal	101	male
SRR341664	T2D	101	male	SRR341702	Normal	101	male
SRR341665	T2D	101	male	SRR341703	Normal	101	male
SRR341666	T2D	101	male	SRR341704	Normal	101	male
SRR341667	T2D	101	male	SRR341705	Normal	101	male
SRR341668	T2D	101	male	SRR341706	Normal	101	male
SRR341669	T2D	101	male	SRR341707	Normal	101	male
SRR341670	T2D	101	male	SRR341708	Normal	101	female
SRR341671	T2D	101	male	SRR341709	Normal	101	female
SRR341672	T2D	101	male	SRR341710	Normal	101	female
SRR341673	T2D	101	male	SRR341711	Normal	101	female
SRR341674	T2D	101	male	SRR341712	Normal	101	male
SRR341675	T2D	101	female	SRR341713	Normal	101	male
SRR341676	T2D	101	female	SRR341714	Normal	101	male
SRR341677	T2D	101	female	SRR341715	Normal	101	male
SRR341678	T2D	101	female	SRR341716	Normal	101	male
SRR341679	T2D	101	male	SRR341717	Normal	101	male
SRR341680	T2D	101	male	SRR341718	Normal	101	male
SRR341681	T2D	101	male	SRR341719	Normal	101	male
SRR341682	T2D	101	male	SRR341720	Normal	101	male
SRR341683	T2D	101	male	SRR341721	Normal	101	male
SRR341684	T2D	101	male	SRR341722	Normal	101	male
SRR341685	T2D	101	male	SRR341723	Normal	101	male

Supplementary Table S48 (continued). Metadata of 241 samples from Qin et al. 2012 study which survived quality control steps (T2D-Qin data set).

Accession No.	Group	Paired-Len (AA)	Gender	Accession No.	Group	Paired-Len (AA)	Gender
SRR341724	Normal	101	male	SRR413619	Normal	101	male
SRR341725	Normal	101	male	SRR413620	Normal	101	male
SRR413556	Normal	101	male	SRR413621	Normal	101	male
SRR413557	Normal	101	female	SRR413622	Normal	101	male
SRR413558	Normal	101	female	SRR413623	Normal	101	male
SRR413559	Normal	101	female	SRR413624	Normal	101	female
SRR413561	T2D	101	female	SRR413625	Normal	101	male
SRR413564	T2D	101	female	SRR413626	Normal	101	male
SRR413565	T2D	101	female	SRR413627	Normal	101	female
SRR413566	T2D	101	male	SRR413628	Normal	101	male
SRR413567	T2D	101	male	SRR413629	Normal	101	female
SRR413568	T2D	101	male	SRR413630	Normal	101	female
SRR413569	T2D	101	male	SRR413631	Normal	101	male
SRR413570	T2D	101	male	SRR413632	Normal	101	male
SRR413571	T2D	101	male	SRR413633	Normal	101	male
SRR413572	T2D	101	male	SRR413634	Normal	101	male
SRR413573	T2D	101	male	SRR413635	Normal	101	female
SRR413574	T2D	101	male	SRR413636	Normal	101	female
SRR413575	Normal	101	male	SRR413637	Normal	101	male
SRR413583	Normal	101	female	SRR413638	Normal	101	female
SRR413586	Normal	101	female	SRR413639	Normal	101	female
SRR413594	Normal	101	male	SRR413640	Normal	101	female
SRR413602	Normal	101	male	SRR413641	Normal	101	female
SRR413603	Normal	101	female	SRR413642	Normal	101	male
SRR413604	Normal	101	male	SRR413643	Normal	101	female
SRR413605	Normal	101	female	SRR413644	Normal	101	female
SRR413606	Normal	101	female	SRR413645	Normal	101	female
SRR413607	Normal	101	male	SRR413646	Normal	101	female
SRR413608	Normal	101	female	SRR413647	Normal	101	female
SRR413609	Normal	101	female	SRR413648	Normal	101	female
SRR413610	Normal	101	female	SRR413649	Normal	101	female
SRR413611	Normal	101	male	SRR413650	Normal	101	female
SRR413612	Normal	101	female	SRR413651	Normal	101	male
SRR413613	Normal	101	female	SRR413652	Normal	101	female
SRR413614	Normal	101	female	SRR413653	Normal	101	male
SRR413615	Normal	101	male	SRR413654	Normal	101	female
SRR413616	Normal	101	male	SRR413655	Normal	101	male
SRR413617	Normal	101	male	SRR413656	Normal	101	female

Supplementary Table S48 (continued). Metadata of 241 samples from Qin et al. 2012 study which survived quality control steps (T2D-Qin data set).

Accession No.	Group	Paired-Len (AA)	Gender	Accession No.	Group	Paired-Len (AA)	Gender
SRR413618	Normal	101	female	SRR413657	Normal	101	male
SRR413658	Normal	101	female	SRR413693	T2D	101	male
SRR413659	Normal	101	female	SRR413695	T2D	101	female
SRR413660	Normal	101	male	SRR413696	T2D	101	male
SRR413661	Normal	101	male	SRR413697	T2D	101	male
SRR413662	Normal	101	female	SRR413698	T2D	101	female
SRR413663	Normal	101	female	SRR413700	T2D	101	male
SRR413664	Normal	101	female	SRR413708	T2D	101	female
SRR413665	Normal	101	female	SRR413714	T2D	101	male
SRR413666	Normal	101	female	SRR413715	T2D	101	male
SRR413667	Normal	101	female	SRR413716	T2D	101	female
SRR413668	Normal	101	male	SRR413721	T2D	101	male
SRR413669	Normal	101	male	SRR413722	T2D	101	male
SRR413670	Normal	101	male	SRR413724	T2D	101	male
SRR413671	Normal	101	male	SRR413725	T2D	101	male
SRR413672	Normal	101	male	SRR413726	T2D	101	female
SRR413673	T2D	101	female	SRR413727	T2D	101	male
SRR413674	T2D	101	female	SRR413728	T2D	101	male
SRR413675	T2D	101	female	SRR413730	T2D	101	male
SRR413677	T2D	101	male	SRR413731	T2D	101	male
SRR413678	T2D	101	male	SRR413732	T2D	101	male
SRR413679	T2D	101	female	SRR413733	T2D	101	male
SRR413680	T2D	101	male	SRR413734	T2D	101	male
SRR413682	T2D	101	male	SRR413735	T2D	101	female
SRR413683	T2D	101	female	SRR413736	T2D	101	female
SRR413686	T2D	101	male	SRR413737	T2D	101	male
SRR413688	T2D	101	female	SRR413738	T2D	101	male
SRR413689	T2D	101	female	SRR413739	T2D	101	female
SRR413690	T2D	101	male	SRR413740	T2D	101	male
SRR413692	T2D	101	female	SRR413741	T2D	101	female
SRR413742	T2D	101	male	SRR413743	T2D	101	female
SRR413744	T2D	101	male	SRR413745	T2D	101	male
SRR413746	T2D	101	female	SRR413747	T2D	101	male
SRR413748	T2D	101	female	SRR413749	T2D	101	male
SRR413750	T2D	101	female	SRR413754	T2D	101	male
SRR413751	T2D	101	male	SRR413757	T2D	101	female
SRR413752	T2D	101	female	SRR413759	T2D	101	male
SRR413753	T2D	101	male	SRR413760	T2D	101	male

Supplementary Table S48 (continued). Metadata of 241 samples from Qin et al. 2012 study which survived quality control steps (T2D-Qin data set).

Accession No.	Group	Paired-Len (AA)	Gender	Accession No.	Group	Paired-Len (AA)	Gender
SRR413761	T2D	101	male	SRR413768	T2D	101	male
SRR413762	T2D	101	male	SRR413769	T2D	101	female
SRR413764	T2D	101	male	SRR413770	T2D	101	male
SRR413765	T2D	101	female	SRR413771	T2D	101	female
SRR413766	T2D	101	female	SRR413772	T2D	101	female
SRR413767	T2D	101	male	SRR413773	T2D	101	male

Supplementary Table S49. Metadata of 145 samples from Karlsson et al. 2013 study (T2D-Karlsson data set).

Run accession	Group	Age (years)	Paired nominal length (bp)
ERR260132	IGT	70.21	150
ERR260133	IGT	70.56	150
ERR260134	IGT	70.46	150
ERR260135	IGT	70.57	150
ERR260136	IGT	70.15	150
ERR260137	IGT	70.16	150
ERR260138	IGT	71.12	150
ERR260139	T2D	70.25	150
ERR260140	T2D	70.15	150
ERR260141	IGT	71.10	150
ERR260142	IGT	70.84	150
ERR260143	IGT	70.35	150
ERR260144	T2D	69.58	150
ERR260145	IGT	70.19	150
ERR260146	IGT	70.45	150
ERR260147	NGT	71.39	150
ERR260148	IGT	70.91	150
ERR260149	T2D	70.14	150
ERR260150	IGT	71.58	150
ERR260151	T2D	71.57	150
ERR260152	T2D	71.24	150
ERR260153	NGT	70.42	150
ERR260154	IGT	71.04	150
ERR260155	T2D	69.08	150
ERR260156	IGT	70.59	150
ERR260157	IGT	70.96	150
ERR260158	IGT	71.31	150
ERR260159	T2D	70.55	150
ERR260160	IGT	69.02	150
ERR260161	T2D	70.39	150
ERR260162	T2D	71.01	150
ERR260163	NGT	70.14	150
ERR260164	IGT	71.01	150
ERR260165	T2D	69.74	150
ERR260166	T2D	71.25	150
ERR260167	T2D	70.97	150

Supplementary Table S49 (Continued). Metadata of 145 samples from Karlsson et al. 2013 study (T2D-Karlsson data set).

Run accession	Group	Age (years)	Paired nominal length (bp)
ERR260168	IGT	70.42	150
ERR260169	T2D	68.96	150
ERR260170	NGT	71.11	150
ERR260171	NGT	70.55	150
ERR260172	IGT	71.01	150
ERR260173	T2D	70.11	150
ERR260174	T2D	70.78	150
ERR260175	NGT	71.02	150
ERR260176	T2D	70.72	150
ERR260177	IGT	70.20	150
ERR260178	T2D	71.12	150
ERR260179	T2D	70.16	150
ERR260180	NGT	71.38	150
ERR260181	T2D	71.36	150
ERR260182	T2D	71.36	150
ERR260183	IGT	71.21	150
ERR260184	IGT	70.12	150
ERR260185	T2D	69.47	150
ERR260186	T2D	70.22	150
ERR260187	IGT	69.06	150
ERR260188	T2D	71.16	150
ERR260189	T2D	70.90	150
ERR260190	T2D	71.39	150
ERR260191	T2D	69.98	150
ERR260192	T2D	70.08	150
ERR260193	NGT	70.25	150
ERR260194	T2D	70.78	150
ERR260195	IGT	69.88	150
ERR260196	T2D	71.47	150
ERR260197	IGT	70.68	150
ERR260198	T2D	71.16	150
ERR260199	T2D	70.52	150
ERR260200	IGT	71.03	150
ERR260201	T2D	71.29	150
ERR260202	T2D	69.97	150
ERR260203	T2D	70.86	150
ERR260204	NGT	70.23	150

Supplementary Table S49 (Continued). Metadata of 145 samples from Karlsson et al. 2013 study (T2D-Karlsson data set).

Run accession	Group	Age (years)	Paired nominal length (bp)
ERR260205	NGT	70.63	150
ERR260206	T2D	70.71	150
ERR260207	T2D	71.49	150
ERR260208	T2D	71.71	150
ERR260209	NGT	69.41	150
ERR260210	T2D	71.24	150
ERR260211	IGT	71.28	150
ERR260214	T2D	71.63	150
ERR260215	NGT	71.26	150
ERR260216	NGT	71.84	150
ERR260217	NGT	71.53	150
ERR260218	NGT	71.44	150
ERR260219	IGT	71.17	150
ERR260220	IGT	71.25	150
ERR260221	NGT	70.47	150
ERR260222	IGT	71.40	150
ERR260223	NGT	71.45	150
ERR260224	NGT	69.67	150
ERR260225	NGT	70.33	150
ERR260226	NGT	69.70	150
ERR260227	NGT	69.74	150
ERR260228	IGT	69.43	150
ERR260229	T2D	70.02	150
ERR260230	NGT	69.87	150
ERR260231	NGT	70.21	150
ERR260232	IGT	69.70	150
ERR260233	IGT	69.36	150
ERR260234	NGT	70.43	150
ERR260235	T2D	69.32	150
ERR260236	IGT	71.00	150
ERR260237	IGT	70.03	150
ERR260238	T2D	69.49	150
ERR260239	T2D	69.98	150
ERR260240	T2D	69.18	150
ERR260241	T2D	69.91	150

Supplementary Table S49 (Continued). Metadata of 145 samples from Karlsson et al. 2013 study (T2D-Karlsson data set).

Run accession	Group	Age (years)	Paired nominal length (bp)
ERR260242	NGT	69.78	150
ERR260243	NGT	69.89	150
ERR260244	NGT	70.07	150
ERR260245	IGT	70.27	150
ERR260246	NGT	70.24	150
ERR260247	IGT	70.96	150
ERR260248	IGT	70.67	150
ERR260249	IGT	69.92	150
ERR260250	NGT	70.02	150
ERR260251	NGT	69.27	150
ERR260252	NGT	69.48	150
ERR260253	NGT	70.01	150
ERR260254	IGT	69.89	150
ERR260255	NGT	69.42	150
ERR260256	NGT	69.49	150
ERR260257	IGT	69.79	150
ERR260258	NGT	69.53	150
ERR260259	NGT	70.24	150
ERR260260	NGT	70.34	150
ERR260261	T2D	68.98	150
ERR260262	IGT	70.70	150
ERR260263	NGT	70.06	150
ERR260264	NGT	70.21	150
ERR260265	NGT	70.85	150
ERR260266	NGT	71.10	150
ERR260267	NGT	69.03	150
ERR260268	NGT	70.05	150
ERR260269	IGT	70.31	150
ERR260270	T2D	69.54	150
ERR260271	T2D	69.32	150
ERR260272	IGT	70.91	150
ERR260273	T2D	70.85	150
ERR260274	T2D	70.52	150
ERR260275	IGT	69.97	150
ERR260276	T2D	70.03	150
ERR275251	IGT	69.15	150
ERR275252	T2D	71.65	150

Supplementary Table S50. Metadata of 53 samples from IBDMDB's HMP Pilot phase Crohn's disease study (CD-HMP data set).

Sample Id	Group	Paired Nominal Length	Sample Id	Group	Paired Nominal Length
CSM5FZ3N_P	CD	101	HSM6XRQB_P	CD	101
CSM5FZ3T_P	CD	101	HSM6XRQC_P	nonIBD	101
CSM5MCVB_P	CD	101	HSM67VDX_P	nonIBD	101
CSM5MCV1_P	CD	101	HSM67VDR_P	nonIBD	101
CSM5MCU4_P	CD	101	HSM67VDT_P	nonIBD	101
CSM5MCWK_P	CD	101	HSM7CYWS_P	CD	101
CSM5MCXF_P	CD	101	HSM7CZ2V_P	CD	101
CSM67U9V_P	CD	101	MSM5LLHR_P	CD	101
CSM67U9X_P	CD	101	MSM5LLIC_P	CD	101
CSM67UAI_P	CD	101	MSM5LLIS_P	CD	101
CSM79HG7_P	CD	101	MSM5LLFG_P	CD	101
CSM79HHW_P	CD	101	CSM6J2H9_P	nonIBD	101
CSM79HJI_P	CD	101	MSM6J2JF_P	nonIBD	101
CSM79HNY_P	CD	101	MSM6J2JH_P	nonIBD	101
ESM5MEDZ_P	CD	101	MSM6J2RG_P	CD	101
ESM5GEYY_P	CD	101	MSM79HBX_P	CD	101
ESM5MEB9_P	CD	101	MSM79H94_P	nonIBD	101
ESM7F5AE_P	CD	101	MSM9VZFJ_P	nonIBD	101
HSM5MD7Z_P	CD	101	MSM9VZLX_P	CD	101
HSM5MD8A_P	nonIBD	101	MSM9VZM4_P	CD	101
HSM5MD82_P	nonIBD	101	PSM6XBQM_P	CD	101
HSM5MD8J_P	CD	101	PSM6XBRK_P	CD	101
HSM5MD8F_P	CD	101	PSM6XBRM_P	CD	101
HSM5MD8H_P	nonIBD	101	PSM6XBVY_P	CD	101
HSM5MD3L_P	CD	101	PSM6XBW1_P	nonIBD	101
HSM5MD5X_P	CD	101	PSM7J1B3_P	CD	101
HSM5MD5Z_P	nonIBD	101			

Supplementary Table S51. Metadata of 62 samples from Swedish twin study of Crohn's disease (CD-Swedish Data set).

Sample Accession	Group	Read length / Nominal Length	Sequencing Technology	Sample Accession	Group	Read length / Nominal Length	Sequencing Technology
SRR053011	control	557	454	SRR090273	case	495	454
SRR053012	control	506	454	SRR090275	case	494	454
SRR053013	control	512	454	SRR495448	case	514	454
SRR053014	case	477	454	SRR497643	case	520	454
SRR053015	case	493	454	SRR497645	case	518	454
SRR053016	case	512	454	SRR497646	case	521	454
SRR053017	case	499	454	SRR497648	case	509	454
SRR053018	case	495	454	SRR497650	case	526	454
SRR053019	case	486	454	SRR497652	case	512	454
SRR053020	control	501	454	SRR497654	case	511	454
SRR053021	control	483	454	SRR497657	case	523	454
SRR053022	control	490	454	SRR497943	case	509	454
SRR053023	control	508	454	SRR497946	case	567	454
SRR053024	case	520	454	SRR497948	case	516	454
SRR053025	control	430	454	SRR497949	case	516	454
SRR053026	control	531	454	SRR497952	case	520	454
SRR053027	control	481	454	SRR504939	case	523	454
SRR053028	control	521	454	SRR495449	case	101	Illumina PE
SRR053029	control	506	454	SRR497642	case	101	Illumina PE
SRR053030	control	528	454	SRR497644	case	101	Illumina PE
SRR053031	control	543	454	SRR497647	case	101	Illumina PE
SRR053032	case	546	454	SRR497649	case	101	Illumina PE
SRR053033	case	440	454	SRR497653	case	101	Illumina PE
SRR053034	case	534	454	SRR497656	case	101	Illumina PE
SRR053035	case	496	454	SRR497944	case	101	Illumina PE
SRR053036	case	509	454	SRR497945	case	101	Illumina PE
SRR054211	case	453	454	SRR497947	case	101	Illumina PE
SRR054212	case	516	454	SRR497950	case	101	Illumina PE
SRR090269	case	517	454	SRR497951	case	101	Illumina PE
SRR090271	case	510	454	SRR504938	case	101	Illumina PE
SRR090272	case	507	454	SRR513399	case	101	Illumina PE

Supplementary Table S52. Metadata of 41 individuals from Bedarf et al. 2017 study (PD-Bedarf Data set).

SRA Sample	Run accession	Group	Paired nominal length
ERS1647316	ERR1912947	control	129
ERS1647331	ERR1912950	parkinson	90
ERS1647293	ERR1912955	parkinson	90
ERS1647327	ERR1912957	control	120
ERS1647282	ERR1912959	parkinson	129
ERS1647305	ERR1912962	control	129
ERS1647298	ERR1912965	parkinson	129
ERS1647289	ERR1912969	parkinson	90
ERS1647329	ERR1912971	control	120
ERS1647302	ERR1912972	parkinson	90
ERS1647284	ERR1912977	parkinson	126
ERS1647318	ERR1912979	control	90
ERS1647299	ERR1912984	parkinson	129
ERS1647281	ERR1912987	parkinson	129
ERS1647330	ERR1912989	control	120
ERS1647323	ERR1912990	control	123
ERS1647304	ERR1912992	control	129
ERS1647277	ERR1912995	parkinson	129
ERS1647335	ERR1912997	parkinson	126
ERS1647278	ERR1913001	parkinson	90
ERS1647319	ERR1913002	control	90
ERS1647324	ERR1913006	control	120
ERS1647317	ERR1913008	control	126
ERS1647292	ERR1913012	parkinson	90
ERS1647313	ERR1913015	control	123
ERS1647285	ERR1913017	parkinson	90
ERS1647296	ERR1913022	parkinson	90
ERS1647312	ERR1913025	control	90
ERS1647321	ERR1913030	control	129
ERS1647288	ERR1913033	parkinson	90
ERS1647326	ERR1913036	control	120
ERS1647291	ERR1913038	parkinson	129
ERS1647309	ERR1913040	control	90
ERS1647311	ERR1913046	control	126
ERS1647286	ERR1913048	parkinson	129
ERS1647300	ERR1913050	parkinson	90
ERS1647315	ERR1913055	control	123
ERS1647297	ERR1913058	parkinson	126
ERS1647303	ERR1913061	control	123
ERS1647325	ERR1913063	control	123
ERS1647295	ERR1913066	parkinson	90

Supplementary Table S53. Metadata of 84 gut microbial samples from Bostonian individuals (Data from Eric Alm's lab: SRP200548).

Biosample Accession	Sample Id	Group	Paired Nominal Length	Biosample Accession	Sample Id	Group	Paired Nominal Length
SAMN11950000	aa0154	Industrialized	101	SAMN11950464	bo0001	Industrialized	101
SAMN11950003	ab0168	Industrialized	101	SAMN11950466	bp0002	Industrialized	101
SAMN11950005	ac0038	Industrialized	101	SAMN11950468	bq0002	Industrialized	101
SAMN11950006	ad0002	Industrialized	101	SAMN11950470	br0001	Industrialized	101
SAMN11950026	ae0024	Industrialized	101	SAMN11950473	bs0008	Industrialized	101
SAMN11950068	af0060	Industrialized	101	SAMN11950475	bt0039	Industrialized	101
SAMN11950070	ag0005	Industrialized	101	SAMN11950477	bu0080	Industrialized	101
SAMN11950071	ah0002	Industrialized	101	SAMN11950478	bv0001	Industrialized	101
SAMN11950074	ai0019	Industrialized	101	SAMN11950481	bw0033	Industrialized	101
SAMN11950075	aj0001	Industrialized	101	SAMN11950482	bx0001	Industrialized	101
SAMN11950077	ak0001	Industrialized	101	SAMN11950485	by0059	Industrialized	101
SAMN11950080	al0025	Industrialized	101	SAMN11950486	bz0001	Industrialized	101
SAMN11950159	am0097	Industrialized	101	SAMN11950489	ca0012	Industrialized	101
SAMN11950288	an0002	Industrialized	101	SAMN11950490	cb0001	Industrialized	101
SAMN11950406	ao0073	Industrialized	101	SAMN11950492	cc0002	Industrialized	101
SAMN11950424	ap0001	Industrialized	101	SAMN11950495	cd0050	Industrialized	101
SAMN11950426	aq0004	Industrialized	101	SAMN11950497	ce0007	Industrialized	101
SAMN11950428	ar0002	Industrialized	101	SAMN11950498	cf0001	Industrialized	101
SAMN11950431	as0033	Industrialized	101	SAMN11950501	cg0014	Industrialized	101
SAMN11950432	at0004	Industrialized	101	SAMN11950503	ch0008	Industrialized	101
SAMN11950434	au0002	Industrialized	101	SAMN11950505	ci0052	Industrialized	101
SAMN11950437	av0107	Industrialized	101	SAMN11950506	cj0001	Industrialized	101
SAMN11950438	aw0001	Industrialized	101	SAMN11950509	ck0028	Industrialized	101
SAMN11950440	ax0001	Industrialized	101	SAMN11950510	cm0001	Industrialized	101
SAMN11950442	ay0001	Industrialized	101	SAMN11950513	cn0006	Industrialized	101
SAMN11950444	az0001	Industrialized	101	SAMN11950514	cp0001	Industrialized	101
SAMN11950447	ba0002	Industrialized	101	SAMN11950516	cq0001	Industrialized	101
SAMN11950448	bd0033	Industrialized	101	SAMN11950519	cr0043	Industrialized	101
SAMN11950449	be0001	Industrialized	101	SAMN11950521	cs0011	Industrialized	101
SAMN11950452	bf0108	Industrialized	101	SAMN11950523	ct0001	Industrialized	101
SAMN11950453	bh0112	Industrialized	101	SAMN11950526	cu0009	Industrialized	101
SAMN11950454	bi0001	Industrialized	101	SAMN11950528	cv0018	Industrialized	101
SAMN11950457	bk0025	Industrialized	101	SAMN11950531	cw0053	Industrialized	101
SAMN11950459	bl0009	Industrialized	101	SAMN11950533	cx0014	Industrialized	101
SAMN11950461	bm0013	Industrialized	101	SAMN11950535	cy0019	Industrialized	101
SAMN11950463	bn0038	Industrialized	101	SAMN11950537	cz0001	Industrialized	101

Supplementary Table S53 (continued). Metadata of 84 gut microbial samples from Bostonian individuals (Data from Eric Alm's lab: SRP200548).

Biosample Accession	Sample Id	Group	Paired Nominal Length	Biosample Accession	Sample Id	Group	Paired Nominal Length
SAMN11950539	da0001	Industrialized	101	SAMN11950552	dg0008	Industrialized	101
SAMN11950542	db0015	Industrialized	101	SAMN11950554	dh0010	Industrialized	101
SAMN11950544	dc0028	Industrialized	101	SAMN11950555	di0001	Industrialized	101
SAMN11950545	dd0001	Industrialized	101	SAMN11950558	dj0016	Industrialized	101
SAMN11950547	de0001	Industrialized	101	SAMN11950560	dk0003	Industrialized	101
SAMN11950549	df0001	Industrialized	101	SAMN11950561	dl0001	Industrialized	101

Supplementary Table S54. Metadata of 35 gut microbial samples from Baka individuals of Cameroon (unpublished data from Eric Alm's lab).

Sample ID	Group	Paired Nominal Length
x0316QC	Non-industrialized	101
x0505YP	Non-industrialized	101
x0658JO	Non-industrialized	101
x0993CA	Non-industrialized	101
x1093PZ	Non-industrialized	101
x1305IL	Non-industrialized	101
x1940EX	Non-industrialized	101
x2040RT	Non-industrialized	101
x2355KF	Non-industrialized	101
x2697DU	Non-industrialized	101
x3032MC	Non-industrialized	101
x3485XE	Non-industrialized	101
x3647FO	Non-industrialized	101
x3790QQ	Non-industrialized	101
x4082LZ	Non-industrialized	101
x4132ZY	Non-industrialized	101
x4324HL	Non-industrialized	101
x4447SN	Non-industrialized	101
x5424UH	Non-industrialized	101
x5739NT	Non-industrialized	101
x5882YV	Non-industrialized	101
x6174TE	Non-industrialized	101
x6416MQ	Non-industrialized	101
x6527EG	Non-industrialized	101
x6839AS	Non-industrialized	101
x7121VB	Non-industrialized	101
x7274GA	Non-industrialized	101
x7466ON	Non-industrialized	101
x7519ZM	Non-industrialized	101
x8566BJ	Non-industrialized	101
x8801UV	Non-industrialized	101
x8921IX	Non-industrialized	101
x9213DG	Non-industrialized	101
x9558WS	Non-industrialized	101
x9901HR	Non-industrialized	101

Supplementary Table S55. Metadata of 50 gut microbial samples from Ethiopian individuals of Gimbichu region (Pasolli et al. 2019).

Sample Accession	Group	Paired Nominal Length	Sample Accession	Group	Paired Nominal Length
SRR8180450	Non-industrialized	101	SRR8784377	Non-industrialized	101
SRR8180449	Non-industrialized	101	SRR8784376	Non-industrialized	101
SRR8180448	Non-industrialized	101	SRR8784375	Non-industrialized	101
SRR8180447	Non-industrialized	101	SRR8784374	Non-industrialized	101
SRR8180446	Non-industrialized	101	SRR8784373	Non-industrialized	101
SRR8784397	Non-industrialized	101	SRR8784372	Non-industrialized	101
SRR8784396	Non-industrialized	101	SRR8784371	Non-industrialized	101
SRR8784395	Non-industrialized	101	SRR8784370	Non-industrialized	101
SRR8784394	Non-industrialized	101	SRR8784369	Non-industrialized	101
SRR8784393	Non-industrialized	101	SRR8784368	Non-industrialized	101
SRR8784392	Non-industrialized	101	SRR8784367	Non-industrialized	101
SRR8784391	Non-industrialized	101	SRR8784366	Non-industrialized	101
SRR8784390	Non-industrialized	101	SRR8784365	Non-industrialized	101
SRR8784389	Non-industrialized	101	SRR8784364	Non-industrialized	101
SRR8784388	Non-industrialized	101	SRR8784363	Non-industrialized	101
SRR8784387	Non-industrialized	101	SRR8784362	Non-industrialized	101
SRR8784386	Non-industrialized	101	SRR8784361	Non-industrialized	101
SRR8784385	Non-industrialized	101	SRR8784360	Non-industrialized	101
SRR8784384	Non-industrialized	101	SRR8784359	Non-industrialized	101
SRR8784383	Non-industrialized	101	SRR8784358	Non-industrialized	101
SRR8784382	Non-industrialized	101	SRR8784357	Non-industrialized	101
SRR8784381	Non-industrialized	101	SRR8784356	Non-industrialized	101
SRR8784380	Non-industrialized	101	SRR8784355	Non-industrialized	101
SRR8784379	Non-industrialized	101	SRR8784354	Non-industrialized	101
SRR8784378	Non-industrialized	101	SRR8784353	Non-industrialized	101

Supplementary Table S56. Metadata of 112 samples from Madagascar (Pasolli et al. 2019).

Sample Accession	Group	Paired Nominal Length	Sample Accession	Group	Paired Nominal Length	Sample Accession	Group	Paired Nominal Length
SRR7658688	Non-industrialized	101	SRR7658646	Non-industrialized	101	SRR7658612	Non-industrialized	101
SRR7658687	Non-industrialized	101	SRR7658645	Non-industrialized	101	SRR7658611	Non-industrialized	101
SRR7658690	Non-industrialized	101	SRR7658644	Non-industrialized	101	SRR7658610	Non-industrialized	101
SRR7658689	Non-industrialized	101	SRR7658643	Non-industrialized	101	SRR7658609	Non-industrialized	101
SRR7658685	Non-industrialized	101	SRR7658642	Non-industrialized	101	SRR7658607	Non-industrialized	101
SRR7658684	Non-industrialized	101	SRR7658640	Non-industrialized	101	SRR7658608	Non-industrialized	101
SRR7658682	Non-industrialized	101	SRR7658641	Non-industrialized	101	SRR7658606	Non-industrialized	101
SRR7658683	Non-industrialized	101	SRR7658638	Non-industrialized	101	SRR7658668	Non-industrialized	101
SRR7658686	Non-industrialized	101	SRR7658639	Non-industrialized	101	SRR7658605	Non-industrialized	101
SRR7658681	Non-industrialized	101	SRR7658637	Non-industrialized	101	SRR7658604	Non-industrialized	101
SRR7658679	Non-industrialized	101	SRR7658635	Non-industrialized	101	SRR7658603	Non-industrialized	101
SRR7658678	Non-industrialized	101	SRR7658634	Non-industrialized	101	SRR7658601	Non-industrialized	101
SRR7658676	Non-industrialized	101	SRR7658636	Non-industrialized	101	SRR7658600	Non-industrialized	101
SRR7658677	Non-industrialized	101	SRR7658633	Non-industrialized	101	SRR7658602	Non-industrialized	101
SRR7658675	Non-industrialized	101	SRR7658631	Non-industrialized	101	SRR7658598	Non-industrialized	101
SRR7658673	Non-industrialized	101	SRR7658632	Non-industrialized	101	SRR7658599	Non-industrialized	101
SRR7658672	Non-industrialized	101	SRR7658630	Non-industrialized	101	SRR7658597	Non-industrialized	101
SRR7658670	Non-industrialized	101	SRR7658629	Non-industrialized	101	SRR7658596	Non-industrialized	101
SRR7658669	Non-industrialized	101	SRR7658628	Non-industrialized	101	SRR7658662	Non-industrialized	101
SRR7658667	Non-industrialized	101	SRR7658627	Non-industrialized	101	SRR7658595	Non-industrialized	101
SRR7658666	Non-industrialized	101	SRR7658626	Non-industrialized	101	SRR7658593	Non-industrialized	101
SRR7658664	Non-industrialized	101	SRR7658625	Non-industrialized	101	SRR7658594	Non-industrialized	101
SRR7658665	Non-industrialized	101	SRR7658624	Non-industrialized	101	SRR7658590	Non-industrialized	101
SRR7658663	Non-industrialized	101	SRR7658680	Non-industrialized	101	SRR7658592	Non-industrialized	101
SRR7658660	Non-industrialized	101	SRR7658623	Non-industrialized	101	SRR7658591	Non-industrialized	101

Supplementary Table S56 (continued). Metadata of 112 samples from Madagascar (Pasolli et al. 2019).

Sample Accession	Group	Paired Nominal Length	Sample Accession	Group	Paired Nominal Length	Sample Accession	Group	Paired Nominal Length
SRR7658661	Non-industrialized	101	SRR7658622	Non-industrialized	101	SRR7658588	Non-industrialized	101
SRR7658659	Non-industrialized	101	SRR7658621	Non-industrialized	101	SRR7658587	Non-industrialized	101
SRR7658656	Non-industrialized	101	SRR7658619	Non-industrialized	101	SRR7658586	Non-industrialized	101
SRR7658658	Non-industrialized	101	SRR7658620	Non-industrialized	101	SRR7658589	Non-industrialized	101
SRR7658657	Non-industrialized	101	SRR7658674	Non-industrialized	101	SRR7658585	Non-industrialized	101
SRR7658654	Non-industrialized	101	SRR7658618	Non-industrialized	101	SRR7658583	Non-industrialized	101
SRR7658655	Non-industrialized	101	SRR7658617	Non-industrialized	101	SRR7658584	Non-industrialized	101
SRR7658653	Non-industrialized	101	SRR7658616	Non-industrialized	101	SRR7658582	Non-industrialized	101
SRR7658651	Non-industrialized	101	SRR7658671	Non-industrialized	101	SRR7658581	Non-industrialized	101
SRR7658650	Non-industrialized	101	SRR7658614	Non-industrialized	101	SRR7658580	Non-industrialized	101
SRR7658649	Non-industrialized	101	SRR7658615	Non-industrialized	101	SRR7658579	Non-industrialized	101
SRR7658648	Non-industrialized	101	SRR7658613	Non-industrialized	101	SRR7658652	Non-industrialized	101
SRR7658647	Non-industrialized	101						
